# SERHL regulates invasion and metastasis through KLF16-PLCB1/PRKCA signaling in gastric cancer cells

**DOI:** 10.1016/j.gendis.2025.101884

**Published:** 2025-10-20

**Authors:** Linjie Hong, Siyang Peng, Yidong Chen, Yanci Xie, Yuting Lei, Jieke Wu, Xiaodong Huang, Xiangyang Wei, Ping Yang, Jieming Zhang, Qiong Yang, Weimei Tang, Xiaosheng Wu, Ying Peng, Zhizhao Lin, Aimin Li, Side Liu, Jiaying Li, Weiyu Dai, Jide Wang

**Affiliations:** a Guangdong Provincial Key Laboratory of Gastroenterology, Department of Gastroenterology, Nanfang Hospital, Southern Medical University, Guangzhou, Guangdong 510515, China; b Department of Gastroenterology, The Second Affiliated Hospital of Fujian Medical University, Quanzhou, Fujian 362000, China; c Department of Gastroenterology, The Key Laboratory of Advanced Interdisciplinary Studies Center, The First Affiliated Hospital of Guangzhou Medical University, Guangzhou, Guangdong 510120, China; d Department of Gastroenterology and Hepatology, Guangdong Provincial People's Hospital, Guangdong Academy of Medical Sciences, Southern Medical University, Guangzhou, Guangdong 510080, China

**Keywords:** Gastric cancer, Invasion, KLF16, Metastasis, Pseudogene, SERHL

## Abstract

Human SERHL, a pseudogene-derived lncRNA, plays a key role in cancer progression. However, the mechanism by which SERHL modulates gastric cancer (GC) is mostly unknown. This study found that SERHL was down-regulated in GC and inversely related to its malignancy. Overexpression of SERHL inhibited cell growth and metastasis in GC cells. Mechanistically, SERHL interacts with KLF16, promoting the degradation of KLF16 via ubiquitination in GC cells. The transcription factor KLF16 binds to the promoters of PLCB1 and PRKCA genes, increasing their expression and enhancing GC invasion and metastasis. SERHL reduces motility by inhibiting the KLF16-PLCB1/PRKCA signaling pathway in GC cells. These results highlight the function and mechanism of SERHL in GC progression, indicating that SERHL has the potential to be a diagnostic marker and therapeutic target in GC.

## Introduction

The 2022 global cancer statistics stated that gastric cancer (GC) was the fifth most prevalent cancer (4.9%) and the fifth leading cause of cancer mortality (6.8%).^1^ Despite several efforts to enhance GC treatment, the 5-year overall survival rate for GC patients remains around 25%, primarily because most patients are diagnosed at an advanced stage.^2^ Consequently, the identification of novel biomarkers and the underlying molecular mechanisms in GC warrants further investigation.^3^^,^^4^

The advancement of whole-genome sequencing technology has revealed that protein-coding genes represent merely 2% of the human genome, with the remainder consisting of non-coding elements: microRNA, long non-coding RNA (lncRNA), and pseudogenes.^5^ Typically, pseudogenes possess comparable DNA sequences to functional genes, which lose their protein-coding ability because of molecular events that include point or frameshift mutations.^6^^,^^7^ Some pseudogenes are transcribed lncRNAs and participate in cancer development and progression.^8^^,^^9^ For example, the pseudogene-derived lncRNA small ubiquitin-like modifier (SUMO1) pseudogene 3 (SUMO1P3) is overexpressed in GC, indicating a poor prognosis.^10^ Keratin 19 pseudogene 3 (KRT19P3) impedes proliferation and metastasis via constitutive photomorphogenic homolog subunit 7A (COPS7A)-modulated nuclear factor kappa B (NF-κB) signaling in GC.^11^ Moreover, double homeobox A pseudogene 10 (DUXAP10) enhances non-small cell lung cancer proliferation and invasion by interacting with lysine-specific demethylase 1 (LSD1) and hampering large tumor suppressor kinase 2 (LATS2) and Ras-related associated with diabetes (RRAD).^12^ Accordingly, pseudogene-expressed lncRNAs have been highlighted as key factors in cancer studies.

Human serine hydrolase-like (SERHL) was identified as a pseudogene-derived lncRNA according to the HGNC database and located at 22q13.2. Usually, pseudogenes are divided into three categories^13^: duplicated pseudogenes,^14^ processed pseudogenes,^15^ and unitary pseudogenes.^16^ Human SERHL is a unitary pseudogene that occurs when protein-coding genes accumulate mutations and lose their coding potential in humans, whereas Serhl remains a coding gene in most animal species (*e.g.*, mice, *Bos taurus*, and *Rattus norvegicus*).^17^ Reports have underscored that SERHL is significantly overexpressed in hepatocellular carcinoma samples in comparison to non-tumor samples during hepatocellular carcinoma recurrence.^18^ In addition, SERHL was significantly associated with the overall survival of hepatocellular carcinoma patients, and the increased SERHL was related to a higher all-cause mortality risk. Nonetheless, the implications and mechanisms of SERHL in the control of GC tumorigenesis and progression remain unknown.

The current study examined SERHL expression and its association with the malignant biological behavior of GC. Mechanistically, SERHL is directly bound to Kruppel-like factor 16 (KLF16) to increase its degradation through ubiquitination; furthermore, KLF16 interacts with the phospholipase C beta 1 (PLCB1) or protein kinase C alpha (PRKCA) promoter to enhance its transcription and expression. Accordingly, we revealed the significance of SERHL-KLF16-PLCB1/PRKCA signaling in GC progression.

## Materials and methods

### Cell lines, cell culture, and reagents

GC cell lines, including AGS, HGC-27, NCI-N87, SNU-216, SNU-1, SNU-719, MKN-45, MKN-74, and GES-1 human normal gastric epithelial cells, were obtained from the Chinese Academy of Medical Science (Shanghai, China). Cells were cultured in base Roswell Park Memorial Institute medium (RPMI-1640, Thermo Fisher Scientific, USA) or Dulbecco's Modified Eagle Medium (DMEM, Thermo Fisher Scientific, USA), supplemented with 10% fetal bovine serum (Thermo Fisher Scientific, USA) and 100 U/mL penicillin/streptomycin (Sigma–Aldrich, USA) under 5% CO_2_ at 37 °C.

### Quantitative PCR

The extraction of total RNA from GC cells and tissues was performed utilizing TRIzol reagent (Invitrogen, USA) as per the manufacturer's guidelines. The RNAs were reverse-transcribed using a PrimeScript 1st Strand cDNA Synthesis Kit (Takara, Japan) into single-stranded cDNAs. The expression levels of mRNA and lncRNA from the generated cDNAs were quantified utilizing a SYBR Green PCR Kit (Takara, Japan) and a Roche LightCycler 480 system. The 2^−ΔΔCt^ method was used for comparative quantification, utilizing human tubulin beta (TUBB), glyceraldehyde 3-phosphate dehydrogenase (GAPDH), or beta-2 microglobulin (B2M) as internal controls. The primer sequences are provided in Table S1.

### Clinical specimens

Eighty-two formalin-fixed and paraffin-embedded GC specimens and relative neighboring healthy tissue specimens were collected from GC patients at Nanfang Hospital (Guangzhou, China) between June 2017 and April 2020. Normal tissues were collected 5 cm away from the tumor edge and immediately stored in liquid nitrogen for subsequent experimentation. The diagnosis of primary GC was validated by two pathologists for patients who had not received chemotherapy, radiotherapy, or molecular-targeted therapy before surgery. The study received ethical approval from Nanfang Hospital, Southern Medical University (NFEC-2017-062).

### Data of survival analysis

The survival analysis of the GSE15459 data was performed and plotted using the Kaplan–Meier plot website (https://kmplot.com/analysis/).

### Subcellular fractionation

Cytoplasmic and nuclear RNA were isolated from human GC cells using a purification kit (Norgenbiotek, Canada) according to the manufacturer's instructions. The cells were lysed, and the nuclear and cytosolic RNAs were isolated from the gelatinous precipitate and supernatant, respectively. The isolated RNA was then reverse-transcribed into cDNA, and quantitative PCR was performed to assess its subcellular distribution. Quality control of the cytoplasmic RNA samples was carried out using GAPDH, while quality control of the nuclear RNA samples was performed using nuclear paraspeckle assembly transcript 1 (NEAT1). The primer sequences used in the quantitative PCR analysis are listed in Table S1.

### *In situ* hybridization analyses

*In situ* hybridization was performed using three SERHL sequence-specific oligonucleotide probes that were synthesized by BOSTER (Wuhan, China): probe 1: 5′-GAAGCAGGGCCTGGAACTGCAGGATACACTCCCC-3′; probe 2: 5′-GACTACTTCGGATACAATCACAGCAACCCTGGCCTCGGCC-3′; probe 3: 5′-CAGCTGGCCTAGACCCCTGGGAGGCCTCCAAGTCCCTAAG-3′. In brief, the 5-mm paraffin sections were deparaffinized, rehydrated, washed, and incubated with pepsin prepared in 3% sodium citrate at 37 °C for 10 min, then subjected to fixation in 4% paraformaldehyde for 10 min, and incubated with nucleotide probes at 42 °C overnight. Post-hybridization washes included rinsing in 2 × SSC, 0.5 × SSC, and 0.2 × SSC buffer at 37 °C for 15 min each. Anti-digoxigenin antibody (1:100; Roche, Germany) was subsequently subjected to incubation at 37 °C for 4 h. The hybridized signals were detected by nitro blue tetrazolium (NBT) and 5-bromo-4-chloro-3-indole phosphate (BCIP) staining, followed by counterstaining with nuclear fast red for 1 min and fixed in an aqueous solution.

### Plasmids, siRNAs, dCas9-sgRNAs, and cell transfection

SERHL, Flag-KLF16, His-PLCB1, HA-PRKCA, ubiquitin, wild-type or mutated promoter region of PLCB1/PRKCA, and truncated SERHL and truncated KLF16 plasmids were provided by Kidan Biosciences (Guangzhou, China). siRNAs targeting KLF16, PLCB1, PRKCA, SMAD-specific E3 ubiquitin protein ligase 1 (SMURF1), and ring finger protein 180 (RNF180) were chemically synthesized by Geneyuan Company (Guangzhou, China) and Genecreate Company (Wuhan, China). sgRNAs targeting SERHL and lentivirus containing dCas9 were provided by Genechem Company (Shanghai, China). A CRISPR/dCas9 experiment was employed to knock down the expression of SERHL, as the interference effect of siRNAs on SERHL proved to be insufficient. Table S2 illustrates the siRNA and sgRNA sequences. Lipofectamine 8000 (Beyotime, China) was utilized to transiently transfect GC cells with these siRNAs.

### 5-Ethynyl-2′-deoxyuridine (EdU) incorporation assay

GC cells were subjected to inoculation in 96-well plates at a density of 1.5 × 10^4^ cells/well and subsequently underwent incubation for 24 h. Following a 2-h incubation with 50 μM EdU-488 (RiboBio, China) at 37 °C, cells were fixed in 4% paraformaldehyde for 30 min and permeabilized with 0.5% Triton X-100 at room temperature for 10 min. Nuclei were stained with Hoechst 33342. The EdU incorporation assay results were observed using an inverted fluorescence microscope (Olympus IX73, Japan) to capture five random fields. The EdU incorporation rate was calculated as the ratio of EdU-positive cells (green) to total Hoechst 33342-positive cells (blue).

### Colony formation assay

Cells (5 × 10^2^ cells/well) were subjected to inoculation in 12-well plates and incubated for 14 days. Colonies were visualized by staining the plates with 0.5% crystal violet following fixation in 4% paraformaldehyde. The colony formation rate was calculated using a microscope.

### *In vitro* Transwell migration and invasion assay

In this study, migration and invasion assays were conducted *in vitro* using 8-μm-pore Transwell migration chambers (BD Biosciences, USA) placed in 24-well plates containing 500 μL medium with 10% fetal bovine serum. For invasion assays, additional Matrigel (BD Biosciences, USA) was added to the Transwell chambers and incubated at 37 °C for 1 h to solidify. GC cells (4 × 10^4^) were resuspended in 200 μL serum-free medium and inoculated into the upper chamber. After an incubation period lasting between 48 h and 72 h, non-migratory cells were removed, chambers were hydrated with phosphate-buffered saline, fixed in 4% paraformaldehyde for 10 min, and stained with a 0.5% crystal violet solution. Cell enumeration was conducted in five randomly selected fields using a microscope. Migration and invasion rates for each group were calculated by normalizing control samples to 100% migration/invasion.

### Wound healing assay

GC cells transfected with different constructs were cultured in 6-well plates until they reached confluence. A wound injury was created using the sterile tip of a micropipette, and subsequent removal of the detached cells was achieved through washing with phosphate-buffered saline. Cells were then subjected to incubation and permitted to migrate for 48 h. Photographs were captured, and the migration index was computed with the following equation: migration index = (original wound width–wound width after healing)/original wound width × 100%. All experiments were performed in triplicate.

### Western blotting

Protein samples were extracted from whole-cell lysates or GC tissues using RIPA buffer and a 100 × protease inhibitor (Thermo Fisher Scientific, USA). Tissue protein extraction involved additional grinding steps. The proteins were separated utilizing sodium dodecyl sulfate-polyacrylamide gel electrophoresis (SDS-PAGE) before being transferred to PVDF membranes (Merck Millipore, USA). The membranes were blocked with 5% skim milk at room temperature for 1 h, with subsequent overnight incubation using primary antibodies at a temperature of 4 °C. Following subsequent incubation of the membranes with secondary antibodies, protein levels were analyzed using ECL substrates (Beyotime, China). GAPDH was used as a reference gene. ImageJ 1.53 was utilized for quantifying the results. Table S3 presents the antibodies utilized within the current investigation.

### Lentiviral infection assay

The lentiviral constructs were purchased from Geneyuan Company (SERHL/KLF16, Guangzhou, China), Genechem Company (dCas9-SERHL, Shanghai, China), Kidan Biosciences (PLCB1/PRKCA, Guangzhou, China), and GenePharma Company (KLF16/PLCB1/PRKCA-shRNA, Jiangsu, China). The detailed constructs are as follows: Control: CMV-MCS-EF1α-mCherry-T2A-Puro; SERHL: CMV-SERHL-EF1α-mCherry-T2A-Puro; Vector: CMV-MCS-3Flag-EF1α-ZsGreen-T2A-Blast; KLF16: CMV-KLF16-3Flag-EF1α-ZsGreen-T2A-Blast; dCas9-Control sgRNA: U6-sgRNA-SV40-Cherry; dCas9-SERHL sgRNA: SERHL-sgRNA-SV40-Cherry; Vector: CMV-MCS-6∗his/HA-EF1-copGFP-Puro; PLCB1: CMV-PLCB1-6∗his-copGFP-Puro; PRKCA: CMV-PRKCA-HA-copGFP-Puro; NC-shRNA: LV-U6-GFP-NC (5′-TTCTCCGAACGTGTCACGT-3′); KLF16-shRNA: LV-U6-GFP-KLF16-shRNA (5′-CAAGCGCUUCACCCGCAGUGA TT-3′); PLCB1-shRNA: LV-U6-GFP-PLCB1-shRNA (5′-AGAAGATAACAGAAGCTAA-3′); PRKCA-shRNA: LV-U6-GFP-PRKCA-shRNA (5′-GCTGTACTTCGTCATGGAATA-3′).

GC cell lines were transduced with lentiviruses in the presence of 5 μg/mL polybrene. After transduction, gene expression in isolated cells was verified by Western blot analysis or quantitative PCR following selection with puromycin (5 μg/mL) or blasticidin (5 μg/mL) for 2 weeks. Confirmed expression cell pools were used for future research.

### Animal studies

Three-to-four-week-old female BALB/c nude mice (nu/nu) were sourced from the Guangdong Medical Laboratory Animal Center (China) and maintained in specific pathogen-free conditions at Southern Medical University (China). The animal experimental instructions utilized throughout the current investigation have been granted approval from the Animal Ethics Committee of Nanfang Hospital (IACUC-LAC-20231008-005). Mice were randomly assigned to different groups (*n* = 5), with the allocation being determined by a sequence of random numbers that were computer-generated.

A xenograft tumor model in nude mice was created to assess GC cell proliferation. AGS cells, stably transfected, were suspended in phosphate-buffered saline at 1 × 10^7^ cells per 0.1 mL and injected into the right flank of nude mice. Following 5 days of resolution of any inflammatory response, tumor size on the mice's back and their body weight were monitored every 3 days, with growth and weight change curves plotted accordingly. After 20 days, the mice were euthanized, and the final tumor size and weight were documented. The tumor volume was determined using the formula *a* × *b*^2^/2, where *a* represents the length and *b* denotes the width. According to the ethical guidelines, the tumor size must not exceed 1500 mm^3^. In this study, the maximum tumor size remained within this specified limit. Paraffin-embedded tumor sections were analyzed immunohistochemically with the Ki-67 antibody to evaluate the *in vivo* proliferative capacity of GC cells. This assessment was performed in three randomly selected fields for each experimental group.

In the lung metastasis model, nude mice were randomly allocated into distinct groups, with each group comprising five mice. A suspension of stably transfected AGS cells (5 × 10^6^ cells in 0.1 mL phosphate-buffered saline per mouse) was administered via injection into the tail veins of the mice. Subsequently, the mice were euthanized and dissected on the 28th–35th day post-injection. The fusion of metastatic lesions poses challenges in accurately quantifying their number. To address this, the area of metastatic loci within each lung tissue sample was quantified using fluorescence imaging by ImageJ software. The calculation is expressed as follows: the percentage of metastatic loci area = (fluorescence area/all lung area) × 100%. Additionally, lung tissues were fixed in 10% formalin. Paraffin-embedded sections, measuring 3 mm, were then prepared for hematoxylin-eosin staining and MMP2 immunohistochemical analysis.

### Immunohistochemistry analyses

Within the context of GC specimens, immunohistochemistry has been utilized for detecting KLF16, PRKCA, and PLCB1 expression. The extracted tissues underwent fixation in 10% formalin, dehydrated using ethanol, then subjected to immersion in paraffin, and sectioned. The sections were subjected to a heat treatment at a temperature of 65 °C for 2 h. Subsequently, they were dewaxed utilizing xylene and rehydrated utilizing a gradient of ethanol. A treatment with 3% hydrogen peroxide was then performed. The sections were then heated in a sodium citrate buffer (10 mM sodium citrate, 0.05% Tween-20, pH 6.0) for 15–20 min. Following this, the sections were inhibited by employing fetal bovine serum for 20 min. The sections were then incubated with a primary antibody at room temperature for 2 h and subsequently probed with 50 μL of a secondary antibody for 1 h. Subsequently, they were reacted with DAB (ZSGB-BIO, China) for 1–5 min, counterstained with hematoxylin for 2 min, and differentiated in hydrochloric acid (0.1 M). After a 10-min rinse, the sections were dried, cleared, and sealed for microscopic examination. For the results of immunohistochemical experiments, a semi-quantitative analysis was conducted using the immunoreactive score (IRS) method. Table S3 presents the antibodies used.

### RNA pulldown and mass spectrometry

Biotin-labeled RNA probes were synthesized using the RNAmax-T7 *in vitro* transcription kit (RiboBio, China) and incubated at 37 °C for 4 h. Template DNA was subsequently eliminated through DNase I treatment. After purification, RNA probes were captured using magnetic beads (Bersin Bio, China), incubated with cell lysates, and eluted with an elution buffer. Proteins eluted and separated by SDS-PAGE were analyzed using mass spectrometry or Western blotting. Liquid chromatography-tandem mass spectrometry has been conducted via FitGene Bio Company (Guangzhou, China). Table S4 lists all mass spectrometry results.

### RNA immunoprecipitation assay

The Magna RIP Kit (Merck Millipore, USA) was employed for the RNA immunoprecipitation assay. Magnetic beads were conjugated with 5 μg specific primary antibodies at room temperature for 30 min before immunoprecipitation. Subsequently, the bead–antibody complexes were incubated with GC cell lysates at 4 °C for 12 h. RNA was isolated, converted to cDNA, and quantified using quantitative PCR. AGS cells were transfected with full-length or truncated Flag-KLF16 plasmids. Cell lysates were prepared for anti-Flag antibody RNA immunoprecipitation following the aforementioned protocol. Table S3 presents the antibodies used, and Table S1 displays the primer sequences.

### Protein stability and degradation assay

GC cells were cultured in 12-well plates and transfected with SERHL or pcDNA3.1 control plasmids for 48 h. To inhibit protein synthesis, 100 μM cycloheximide (Selleck, China) was administered at various time intervals (0, 0.25, 0.5, 1, 2, or 4 h). Cellular proteins were extracted and quantified using a bicinchoninic acid assay (Thermo Fisher Scientific, USA). The expression level of endogenous KLF16 was determined through Western blot analysis using ImageJ 1.53 software. To suppress protein degradation, a 10 μM concentration of carbobenzoxy-L-leucyl-L-leucyl-L-leucinal (MG132; Selleck, China), a 26 s proteasome inhibitor, was applied for 6 h before cell lysis. The degradation of KLF16 was assessed through Western blotting. The levels of KLF16 expression in the degradation experiments were validated through Western blot analysis.

### Protein ubiquitination assay

GC cells, co-transfected with ubiquitin, SERHL, or control plasmids, or stably transduced with dCas9-SERHL and dCas9-Control, were treated for subsequent use. Cells were treated with 10 μM MG132 for 6 h, harvested, and lysed to extract total proteins. These proteins were immunoprecipitated with the anti-KLF16 antibody on protein A/G beads (Selleck, China) at 4 °C overnight. Proteins were subjected to incubation and separated in a 10% SDS-PAGE gel. Ubiquitinated KLF16 was detected using an antibody against ubiquitination, and grayscale analysis was employed for quantification of the levels of ubiquitinated protein.

### RNA sequencing analysis

MKN-74 cells were transduced with either KLF16-shRNA or Scr-shRNA and were then prepared at a concentration of 2 × 10^7^ cells per sample. Each experimental group consisted of three independent replicate samples. Total RNA was extracted using the TRIzol-chloroform-isopropanol-alcohol method, followed by quality control assessment. Subsequently, RNA sequencing analysis was conducted using the Novaseq 6000 system (Illumina, USA) by Huayin Health Medical Group Co., Ltd. (Guangzhou, China). The fragments per kilobase million (FPKM) was calculated to quantify transcript expression levels. Fold change (FC) was defined as the ratio of the KLF16-shRNA group level to the Scr-shRNA group level. KEGG analysis for differential genes was performed using the WebGestalt website (https://www.webgestalt.org/).

### Promoter analysis

Promoters were operationally defined as the 2.2 kb (−2000 to +200 bp) sequence upstream of the PLCB1/PRKCA gene. The KLF16 binding sites within the PLCB1/PRKCA promoter region, specifically the PLCB1 site1 sequence (−65 to −54: 5′-CCCCCGCCCCC-3′), PRKCA site1 sequence (+173 to +183: 5′-CCCCCGCCCAC-3′), and PRKCA site2 sequence (+90 to +100: 5′-GCCGCGCCCCC-3′), were analyzed for potential binding using the JASPAR database. Subsequent point mutations were deliberately introduced within the KLF16 binding site region.

### Chromatin immunoprecipitation assay

In brief, GC cells (2 × 10^7^) were subjected to treatment via 1% formaldehyde at room temperature for 10 min to activate DNA-protein cross-linking. The chromatin was subjected to sonication, resulting in the generation of fragmented DNA ranging in size from 200 to 800 bp based on the guidelines outlined by the SimpleChIP Enzymatic Chromatin IP Kit (Cell Signaling, USA). Chromosome fragments were captured with bead-antibody complexes, washed, eluted, and purified. Finally, PCR was used to detect the fragments of PLCB1/PRKCA promoter containing the predicted KLF16 binding site. Table S3 presents the antibodies used, and Table S1 displays the primer sequences.

### DNA pulldown assay

GC cell genomic DNA was isolated using a SteadyPure Universal Genomic DNA Extraction Kit (Accurate Biotechnology, China). Biotin-labeled DNA probes were generated by PCR using GC cell genomic DNA and the primers containing a 5′ biotin tag. Following nucleic acid electrophoresis confirmation, DNA probes were combined with streptavidin magnetic beads (Bersin Bio, China), incubated with cell lysates, and eluted using an elution buffer. Protein products were analyzed using SDS-PAGE followed by Western blot detection.

### Luciferase reporter assay

GC cells were inoculated in 24-well plates and transfected with different plasmids. The levels of firefly and Renilla luciferase activities were quantified using the Duo-Lite Luciferase Assay System (Vazyme, China), with firefly luciferase activity being standardized to Renilla luciferase activity. Each experiment was repeated at least three times.

### Co-immunoprecipitation assay

In summary, protein A/G magnetic beads (Selleck, China) were utilized to incubate with antibodies at 4 °C for 2 h. Following multiple washes, GC cell lysates were added for immunoprecipitation at 4 °C for 12 h. Subsequent to centrifugation, protein A/G magnetic beads were eluted and denatured by 1 × loading buffer. Western blot analysis was employed to detect the target proteins. Table S3 presents the antibodies used.

### Statistical analysis

SPSS version 23.0 software (SPSS Inc., USA) and GraphPad Prism 8 (GraphPad Software, USA) were utilized to conduct statistical analyses. All investigations were performed in triplicate to determine statistical significance. Quantitative data were presented as mean ± standard deviation. For normally distributed continuous variables, Student's *t*-test or one-way analysis of variance (ANOVA) was used where appropriate, whereas for other comparisons between qualitative variables, the Mann–Whitney *U* test was applied. The Pearson correlation coefficient was employed to evaluate the correlation. Statistical significance was identified as a two-tailed *P* < 0.05.

## Results

### SERHL down-regulation in GC tissues and cells

**Figure 1:**
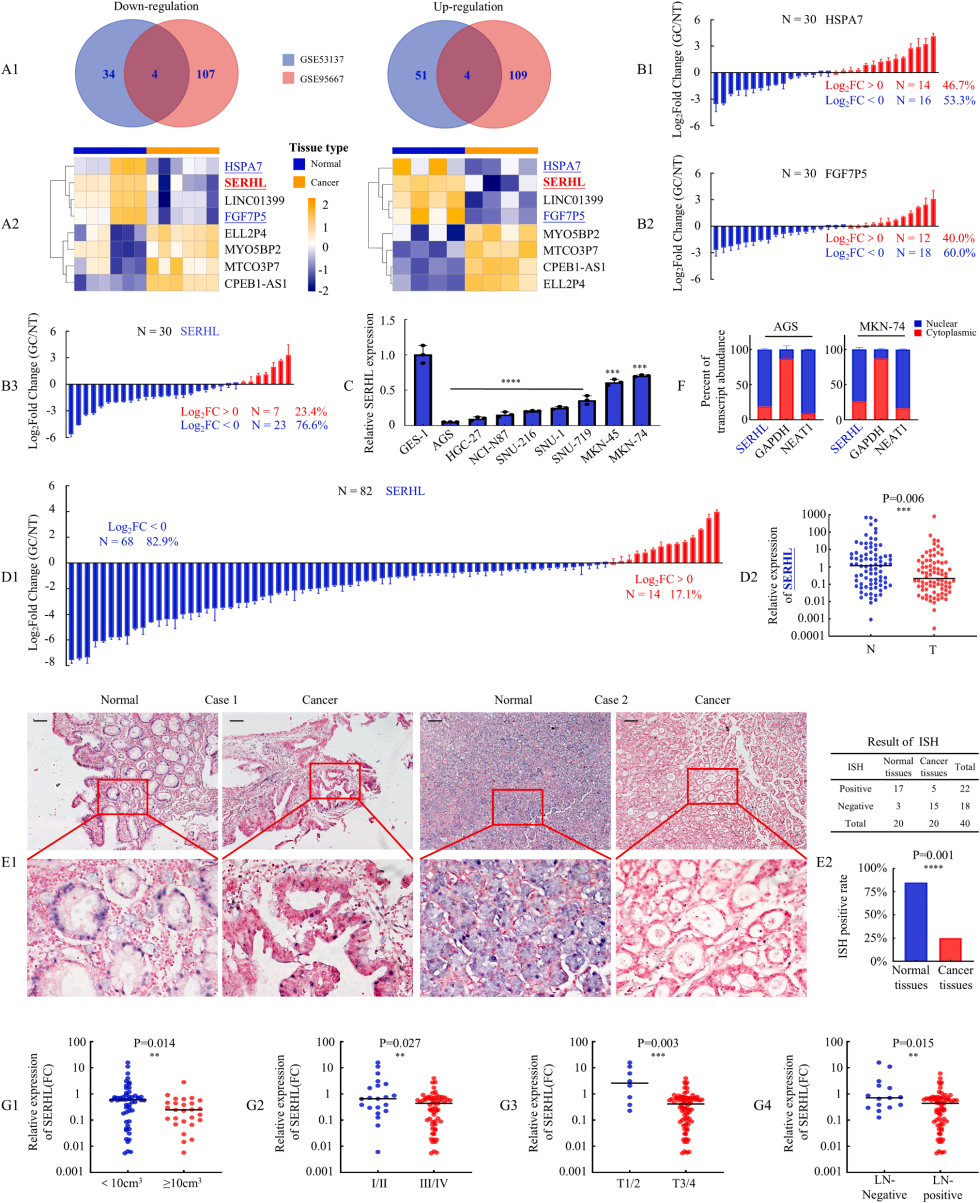
SERHL is down-regulated in gastric cancer (GC). **(A)** Datasets GSE53137 and GSE95667 helped identify differentially expressed lncRNAs between GC and adjacent normal gastric tissues, with intersections shown in Venn diagrams. A heatmap reveals low expression of three pseudogene-derived lncRNAs-SERHL, FGF7P5, and HSPA7 in GC tissues. **(B1–3)** The waterfall plot of expression levels of three pseudogene-derived lncRNAs in GC versus normal tissues, as measured by quantitative PCR. **(C)** SERHL expression in human GC-derived cells and the normal GES-1 cell line was analyzed through quantitative PCR. ∗∗∗*P* < 0.01 and ∗∗∗∗*P* < 0.001 for GC cell lines versus GES-1 cell line. Error bars indicate mean ± standard deviation from three independent trials. **(D1/2)** In 82 pairs of gastric cancer and normal tissues, SERHL expression was measured by quantitative PCR and depicted in a waterfall plot (D1) or a scatter plot (D2), with B2M used as the internal control. ∗∗∗*P* < 0.01 for Normal (N) versus Tumor (T). **(E1/2)** Typical *in situ* hybridization images of SERHL in GC tissues and normal gastric mucosa from 20 cases (E1). Comparison of the *in situ* hybridization positive detection rate between different groups (E2). ∗∗∗∗*P* < 0.001. Scale bars, 100 μm. **(F)** SERHL localization in AGS and MKN-74 cells was identified through subcellular fractionation, setting GAPDH and NEAT1 as cytoplasmic and nuclear controls. **(G)** The relationship between SERHL expression and tumor size (G1), clinical stages (G2), local invasion (G3), or lymph node-metastasis (G4). ∗∗*P* < 0.05 and ∗∗∗*P* < 0.01.

To explore the implications of lncRNAs in GC, we carried out an integrative analysis of GC microarray profiles encompassing GSE53137 and GSE95667 from GEO datasets expressed over the threshold (3-FC) between GC tissues and normal gastric epithelium. Result showed that 38 lncRNAs were down-regulated and 55 were up-regulated in GSE53137, while 111 lncRNAs were down-regulated and 113 were up-regulated in GSE95667 (Fig. 1A1). Intersecting both datasets indicated that four annotated lncRNAs, including three pseudogenes, were down-regulated, and four lncRNAs, including three pseudogenes, were up-regulated (Fig. 1A2). We subsequently focused on the 3 down-regulated pseudogenes because of their enhanced potential for use as early diagnostic markers. The relative HSPA7, FGF7P5, and SERHL expression in 30 pairs of matched normal (N) and cancerous (T) gastric tissues were measured via quantitative reverse transcription PCR. Compared with normal gastric tissues, the low HSPA7, FGF7P5, and SERHL expression rates in GC tissues were 53.3% (16/30), 60.0% (18/30), and 76.6% (23/30), respectively (Fig. 1B1–3), with SERHL showing the most significantly down-regulated (Fig. 1B3). Therefore, SERHL was selected for subsequent studies.

SERHL is a unitary pseudogene that occurs when protein-coding genes accumulate mutations in humans, whereas Serhl is the coding gene in mice (Fig. S1).^17^ Bioinformatics analysis revealed that SERHL was relatively overexpressed in normal human gastric tissue but significantly down-regulated in GC tissue, as demonstrated using the GEPIA and Firebrowse databases (Fig. S2). Hence, SERHL may be a candidate tumor suppressor gene.

First, we deployed the quantitative PCR assay to determine SERHL expression in 8 human GC cell lines and the immortalized gastric mucosal epithelium line GES-1, manifesting relatively lower SERHL expression in all GC cells compared with normal cell lines (Fig. 1C). Second, we utilized quantitative PCR to confirm SERHL expression in 82 pairs of GC tissues and matched non-tumor tissues, finding that SERHL was significantly down-regulated in GC (Fig. 1D). Additionally, the *in situ* hybridization assay revealed lower SERHL expression in GC tissues than in normal tissues. Moreover, SERHL displayed a main nuclear localization in GC cells, with partial cytoplasmic localization (Fig. 1E). Similar to the *in situ* hybridization assay results, the subcellular fractionation experiment elucidated that nuclear SERHL distribution was significantly higher than the cytoplasm (Fig. 1F).

To ascertain whether SERHL expression was associated with GC progression, the interconnection between SERHL and clinicopathologic status was analyzed in 82 GC patients. Figure 1G1–4 and Table S5 reveal strong correlations between SERHL expression and tumor size (< 10 cm^3^ *vs*. ≥ 10 cm^3^; Fig. 1G1; *P* = 0.014), TNM stage (I/II *vs*. III/IV; Fig. 1G2; *P* = 0.027), local invasion (T1/2 *vs*. T3/4; Fig. 1G3; *P* = 0.003), and lymph-node metastasis (lymph-node-negative *vs*. lymph-node-positive; Fig. 1G4; *P* = 0.015). Nonetheless, the findings displayed no significant relationships between SERHL expression and other clinicopathological attributes: gender (male *vs*. female; *P* = 0.860), age (< 60 *vs*. ≥ 60 years; *P* = 0.941) and differentiation (well/moderate *vs*. poor; *P* = 0.847; Table S5). Altogether, SERHL expression was impaired in GC and negatively related to GC metastasis.

### Ectopic expression of SERHL attenuates GC malignant behavior

**Figure 2:**
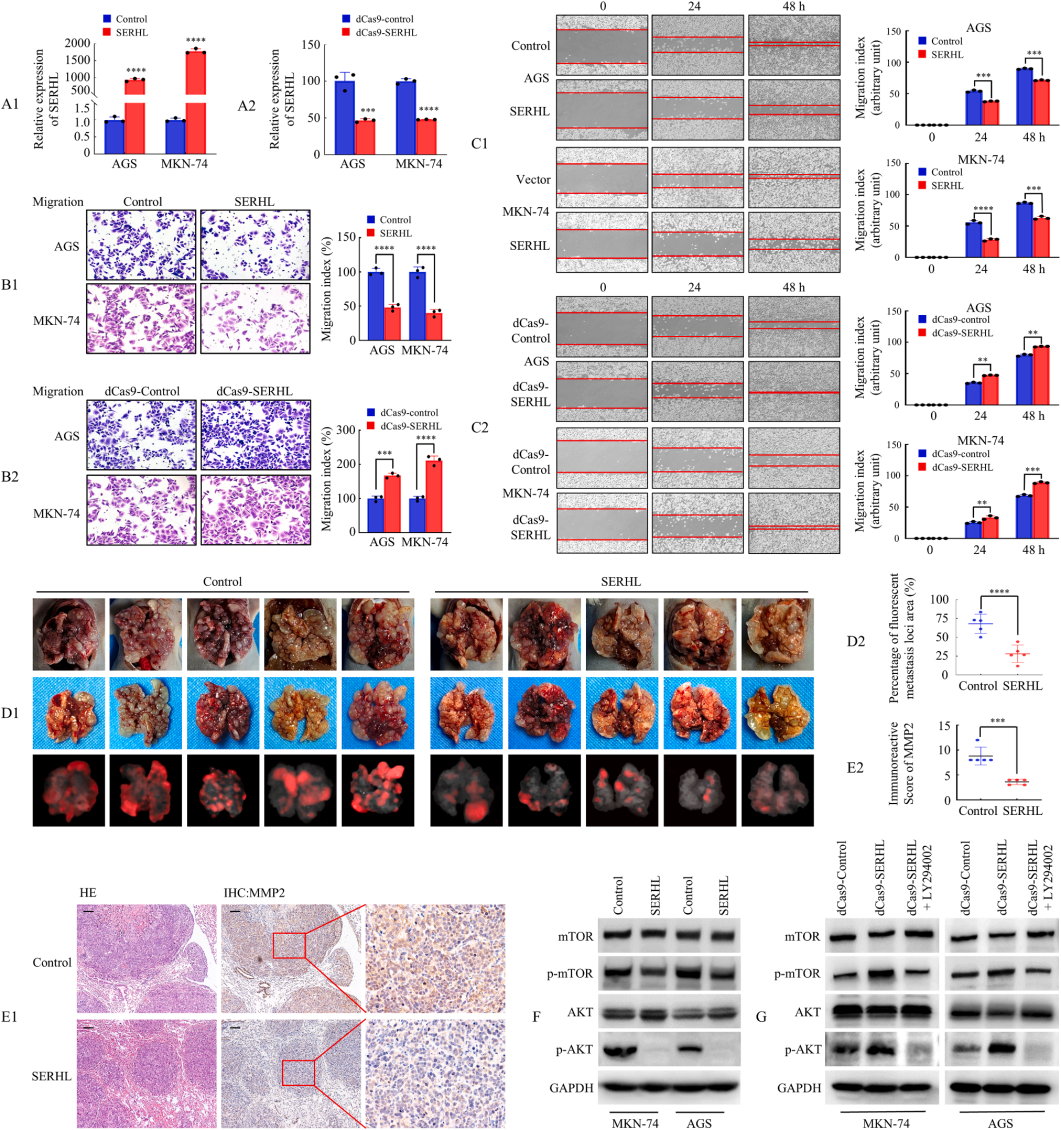
Ectopic SERHL expression repressed gastric cancer cell invasion and metastasis. **(A)** Overexpression (A1) or knockdown (A2) of SERHL in transfected AGS and MKN-74 cell lines were analyzed by quantitative PCR. ∗∗∗∗*P* < 0.001 for Control *vs*. SERHL; ∗∗∗*P* < 0.01 and ∗∗∗∗*P* < 0.001 for dCas9-control *vs*. dCas9-SERHL. **(B1/2)** Transwell assays revealed the effects of SERHL on the migration of AGS and MKN-74 cells. ∗∗∗∗*P* < 0.001 for Control *vs*. SERHL; ∗∗∗*P* < 0.01 and ∗∗∗∗*P* < 0.001 for dCas9-control *vs*. dCas9-SERHL. Data represent mean ± standard deviation. **(C)** Gastric cancer cell motility was detected using the wound healing assay, with bar graphs on the right quantifying their mobility. ∗∗*P* < 0.05, ∗∗∗*P* < 0.01, and ∗∗∗∗*P* < 0.001. **(D1/2)** White light and fluorescence images showed tumor metastases forming in the lungs of nude mice after tail vein injection (D1). Quantification results of the lung metastatic foci area were presented by scatter plots (D2). ∗∗∗∗*P* < 0.001. **(E1/2)** Typical pictures of the hematoxylin-eosin and immunohistochemical staining in Vector and SERHL groups of nude mice lungs (E1). The immunoreactive score (IRS) was compared between different groups (E2). ∗∗∗*P* < 0.01. Scale bars, 100 μm. **(F, G)** The analysis of AKT/mTOR signaling pathway in SERHL overexpression (F) or knockdown (G) gastric cancer cells. AKT pathway inhibitor LY294002 was used to inhibit AKT/mTOR activation.

To ascertain the biological function of SERHL in GC, we used AGS and MKN-74 cells to establish SERHL stably expressing cell lines via lentivirus infection. Using quantitative PCR, we validated the successful overexpression (OE) or dCas9/sgRNA-induced knockdown (KD) of SERHL (Fig. 2A1, A2). Transwell assays, with or without Matrigel, elucidated that SERHL OE significantly decreased migration and invasion (Fig. 2B1; Fig. S3A1), whereas its KD had the opposite effect on AGS and MKN-74 cells (Fig. 2B2; Fig. S3A2). The wound healing experiments showed that forced SERHL expression weakened the migration index (Fig. 2C1), whereas its KD produced the opposite results in GC cells (Fig. 2C2).

Additionally, a nude mouse tail vein metastasis model was employed to ascertain the functionality of SERHL *in vivo*, indicating that SERHL OE reduced the percentage of fluorescent metastasis loci area in the lung (Fig. 2D1, D2). Hematoxylin-eosin staining was utilized to confirm the presence of lung metastases, while immunohistochemical staining of MMP2 revealed a reduction in metastatic potential within SERHL-OE cells (Fig. 2E).

EdU incorporation and colony formation assays were utilized to explore the SERHL influence on cell proliferation *in vitro*. The results showed SERHL OE significantly reduced (Fig. S3B1, S3C1) and SERHL KD significantly increased cell-proliferative capability compared with the controls (Fig. S3B2, S3C2).

To further investigate the effect of SERHL on cell proliferation *in vivo*, AGS/SERHL and control cells were subcutaneously injected into nude mice (Fig. S3D). Tumor weight and volume were significantly reduced in the SERHL group compared with the control group (Fig. S3E1, S3E2). The presence of cancerous tissue sections was validated by hematoxylin-eosin staining (Fig. S3F1). Immunohistochemical analysis manifested that Ki-67 expression was lower in SERHL-OE tumors than in controls (Fig. S3F2).

Previous studies have indicated that the pseudogene-mediated deregulation of AKT/mTOR signaling is connected with the malignant phenotype of tumors.^19^, ^20^, ^21^ Consequently, we investigated the mechanism by which SERHL influences GC migration and proliferation. Our findings demonstrated that SERHL OE reduced AKT and mTOR phosphorylation without changing their total protein levels (Fig. 2F). Conversely, SERHL KD resulted in increased AKT and mTOR phosphorylation levels (Fig. 2G). Furthermore, LY294002, a broad-spectrum PI3K inhibitor, effectively inhibited AKT and mTOR phosphorylation and was capable of counteracting SERHL KD-provoked AKT/mTOR signaling activation (Fig. 2G).

In summary, SERHL demonstrated an inhibitory effect on the invasive capacity and proliferation of GC cells.

### Identification of proteins that bind to SERHL

**Figure 3:**
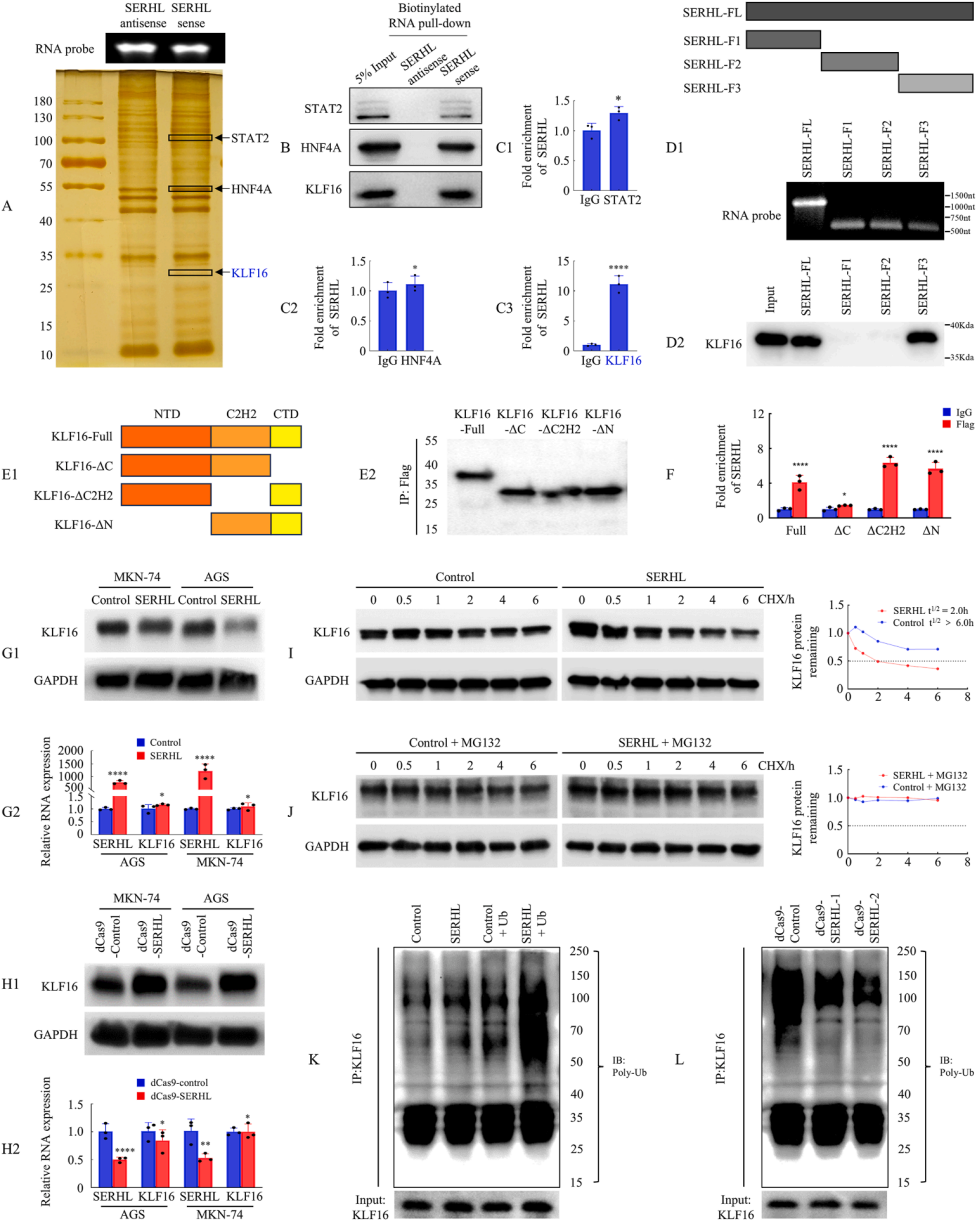
SERHL interacts with KLF16 in gastric cancer cells. **(A)** Silver staining was used to identify proteins from the RNA pulldown assay in AGS cells. **(B)** Biotin-labeled RNA pulldown and Western blotting assays showed the STAT2, HNF4A, or KLF16 protein pulled down by sense or antisense SERHL from lysates of AGS cells. **(C)** The interaction between the three transcription factor proteins and SERHL was detected using an RNA immunoprecipitation assay in AGS cells. ∗*P* > 0.05 for IgG *vs*. STAT2 or HNF4A; ∗∗∗∗*P* < 0.001 for IgG *vs*. SERHL. **(D1/2)** Analysis of KLF16 binding to SERHL truncations was performed using an RNA pulldown assay. Diagrams of SERHL truncations (D1 top) and RNA probe verification via electrophoresis (D1 bottom) are shown. **(E1/2)** With schematics of KLF16 domains provided (E1), full-length and truncated KLF16 plasmids were constructed and verified using Western blotting with an anti-FLAG antibody (E2). **(F)** RNA immunoprecipitation assay analysis of KLF16 structure domains binding to SERHL. ∗*P* > 0.05 for IgG *vs*. anti- KLF16-ΔC; ∗∗∗∗*P* < 0.001 for IgG *vs*. anti-KLF16-Full, anti-KLF16-ΔC2H2, or anti-KLF16-ΔN. **(G1/2, H1/2)** Total proteins or RNA from SERHL overexpression (OE) (G) or knockout (KD) (H) in gastric cancer cells were analyzed by Western blotting (G1, H1) or quantitative PCR (G2, H2) to assess KLF16 expression. ∗*P* > 0.05 and ∗∗∗∗*P* < 0.001 for Control *vs*. SERHL; ∗*P* > 0.05, ∗∗*P* < 0.05, and ∗∗∗∗*P* < 0.001 for dCas9-Control *vs*. dCas9-SERHL. **(I)** AGS cells with SERHL plasmid or control were treated with cycloheximide (100 μM) for the indicated periods, with KLF16 protein levels analyzed by Western blotting. **(J)** AGS cells with SERHL or control plasmid were treated with cycloheximide and MG132 (10 μM), with KLF16 protein levels analyzed by Western blotting. **(K, L)** Ubiquitin levels of KLF16 were measured in SERHL OE (K) or KD (L) AGS cells without or with ubiquitin plasmids using immunoprecipitation (IP) and Western blotting. IB, immunoblotting.

The above findings revealed that SERHL was primarily present within the nucleus (Fig. 1E and F), suggesting that SERHL might participate in governing gene transcription,^22^ variable splicing of mRNA precursor molecules, or interacting with specific proteins.^23^, ^24^, ^25^ Therefore, RNA pulldown assays were deployed, aiming at determining SERHL-related proteins in AGS cells. Following 10% SDS-PAGE and silver staining, we detected the differentially expressed bands through comparison with the antisense control and exposed them to mass spectrometry in AGS cells. The outcomes manifested 236 proteins that interacted with SERHL (Table S4), including 3 transcription factors, STAT2, HNF4A, and KLF16 (Fig. 3A; https://jaspar.elixir.no/). Transcription factors are implicated in many essential biological processes in cancer.^26^^,^^27^ Thus, we selected these three proteins for further validation. Initially, the interaction probabilities of SERHL with STAT2, HNF4A, or KLF16 were predicted by the RF classifier and SVW classifier (RPISeq database, http://pridb.gdcb.iastate.edu/RPISeq/), revealing that SERHL probably directly interacts with the three transcription factors (Fig. S4A1–S4A3). Further validation indicated that the three transcription factors were readily detected in the SERHL pulldown complex but not in control samples pulled down by SERHL antisense transcript (Fig. 3B). Additionally, RNA immunoprecipitation was carried out, aiming at verifying whether candidate proteins bound to SERHL, using antibodies against three transcription factors, setting a nonspecific antibody (IgG) as a control. Result showed that KLF16 directly interacted with SERHL while STAT2 or HNF4A could not bind to SERHL in AGS cells (Fig. 3C1–C3). The findings suggest that SERHL and KLF16 can form a complex *in vitro*.

lncRNAs have been reported to function as unstructured sequences, and the functions of these RNAs may rely more on their linear sequences than their conserved secondary structures.^28^^,^^29^ Seeking the identification of the unique binding fragments, we deployed a series of deletion mutants of SERHL to map the KLF16-binding region: FL: full-length; F1: 1–450 bp; F2: 450–900 bp; F3: 901–1357 bp (Fig. 3D1). The *in vitro* binding assay outcomes showed that KLF16 interacted with FL and F3 regions containing SERHL 3′ end (Fig. 3D2).

For further determination of the specific SERHL binding domains in KLF16, we constructed KLF16-FL (full-length), KLF16-ΔC (C-terminal deletion domain), KLF16-ΔC2H2 (C2H2 deletion domain), and KLF16-ΔN (N-terminal deletion domain) plasmids with Flag-tag (Fig. 3E). Besides KLF16-FL, SERHL could combine with the KLF16-ΔC2H2 and KLF16-ΔN but not KLF16-ΔC (Fig. 3F), confirming that SERHL specifically binds to the KLF16 C-terminal domain in GC cells.

As many lncRNAs can govern binding protein stability,^30^^,^^31^ a conjectural rationale for this phenomenon is that SERHL may impact KLF16 protein stability. To substantiate this assumption, the probable mechanisms behind the SERHL-mediated regulation of KLF16 expression were explored, indicating that SERHL OE down-regulated KLF16 protein (Fig. 3G1) and *vice versa* (Fig. 3H1). Unexpectedly, neither SERHL OE nor KD affected KLF16 mRNA levels in MKN-74 and AGS cells compared with respective controls (Fig. 3G2, 3H2). These studies suggest that SERHL may govern KLF16 protein stability. Accordingly, we treated SERHL-OE and control AGS cells with cycloheximide to study the stability of KLF16 protein, demonstrating that SERHL OE impaired KLF16 protein stability and facilitated its degradation (Fig. 3I).

The proteasome represents a crucial mechanism for protein degradation. Here, we defined whether SERHL governed KLF16 protein stability by altering its proteasomal degradation by assessing the impact of the proteasome inhibitor MG132 on SERHL-mediated modifications in KLF16 stability in AGS cells, showing abrogated SERHL-mediated suppression of KLF16 in MG132 presence (Fig. 3J). Since ubiquitination is essential for proteasomal protein degradation, we examined how SERHL affected the ubiquitination of KLF16. SERHL OE enhanced (Fig. 3K) while SERHL KD significantly suppressed KLF16 polyubiquitination (Fig. 3L).

UbiBrowser^32^ (http://ubibrowser.bio-it.cn/ubibrowser_v3/) was used to identify 27 potential ubiquitin E3 ligases for KLF16. SMURF1^33^ and RNF180^34^, with the highest scores, were chosen for further study (Fig. S4B). Co-immunoprecipitation experiments showed that KLF16 bound to SMURF1 and RNF180 (Fig. S4C). After co-transfection with SERHL and SMURF1/RNF180-siRNAp (siRNAp, siRNA pool: siRNA1/2/3) (Fig. S4D), Western blot analysis revealed that inhibiting RNF180 expression, rather than SMURF1, reversed SERHL-induced KLF16 down-regulation (Fig. S4E). Cycloheximide chase (Fig. S4F) and ubiquitination immunoprecipitation experiments (Fig. S4G) confirmed that RNF180 inhibition partially counteracted SERHL-induced KLF16 ubiquitination and degradation.

Altogether, SERHL may have a direct interaction with KLF16 and enhance its ubiquitination and degradation via E3 ligase RNF180 in GC cells.

### The SERHL–KLF16 axis regulates GC cell invasion and metastasis

**Figure 4:**
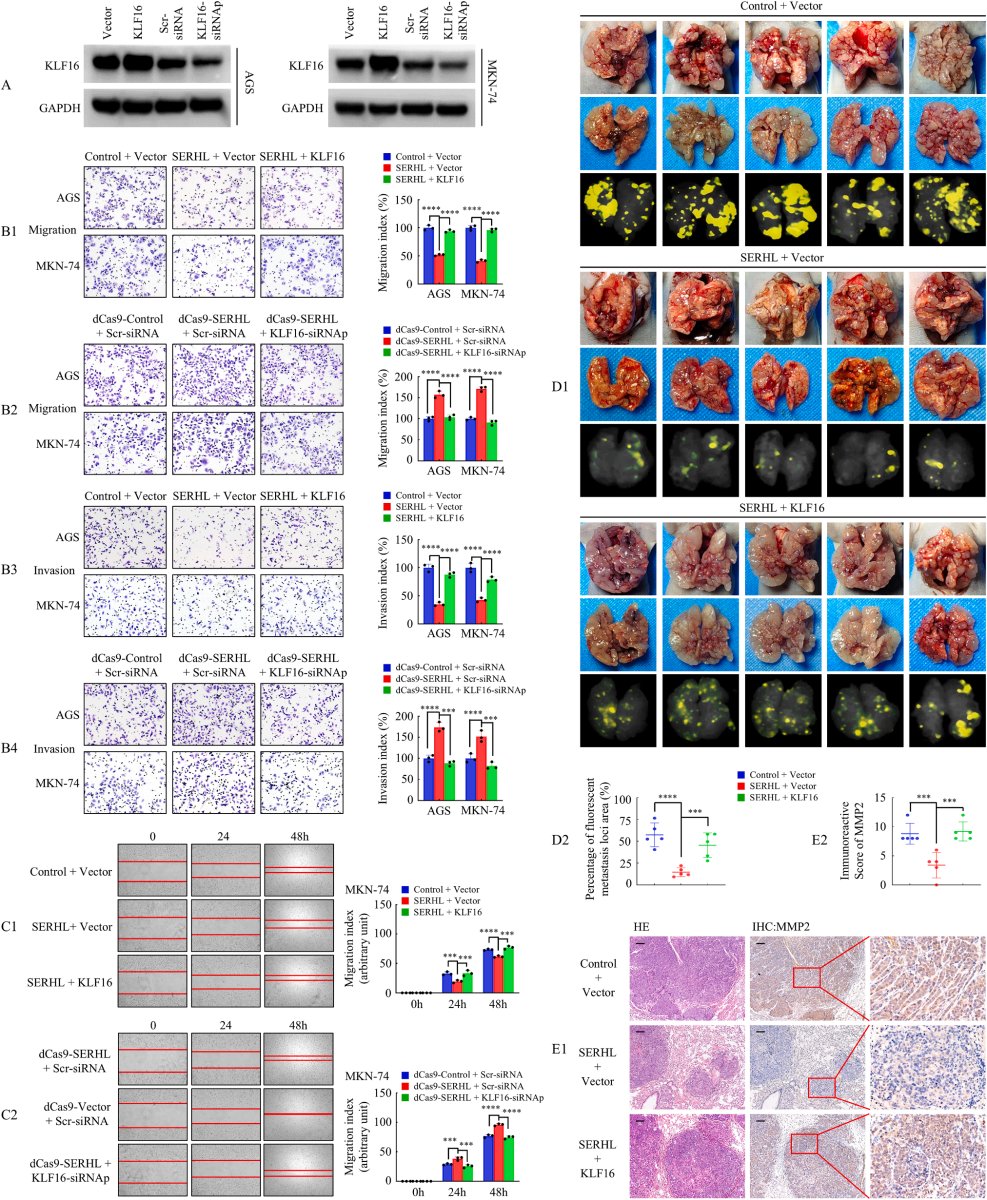
KLF16 mediates the effects of SERHL on tumor invasion in gastric cancer cells. **(A)** KLF16 plasmid and siRNA transfection efficiency were evaluated by Western blotting. **(B1–4)** Migration and invasion changes in various groups of gastric cancer cells were assessed using Transwell migration and invasion assays. ∗∗∗∗*P* < 0.001. **(C1/2)** The MKN-74 cell 2D migration was examined with a monolayer wound healing assay. ∗∗∗*P* < 0.01 and ∗∗∗∗*P* < 0.001. **(D1/2)** Mice with tail vein injection of AGS cells were divided into three groups: Control + Vector, SERHL + Vector, and SERHL + KLF16 (*n* = 5 per group). White light and fluorescence images showed lung tumor metastases (D1), and scatter plots quantified lung metastatic foci area (D2). ∗∗∗*P* < 0.01 and ∗∗∗∗*P* < 0.001. **(E1/2)** Tumor metastases in the lungs were detected by hematoxylin-eosin staining and MMP2 immunohistochemical staining (E1). The immunoreactive score (IRS) was compared between different groups (E2). ∗∗∗*P* < 0.01. Scale bars, 100 μm.

To test whether SERHL regulated GC cell biological function via KLF16, we first established KLF16 OE plasmid or small interfering RNA (siRNA) in AGS and MKN-74 cells and verified using Western blot assays (Fig. 4A). The above results suggested that SERHL suppressed GC cell invasion and metastasis (Fig. 2B1, B2; Fig. S3A1 and S3A2). Next, we co-OE or co-KD SERHL and KLF16 in GC cells. Transwell and wound healing assays indicated that co-OE enhanced cellular motility, counteracting the inhibitory effects observed with SERHL OE alone (Fig. 4B1, B3, C1; Fig. S5A1). Conversely, co-KD reversed this effect (Fig. 4B2, B4, C2; Fig. S5A2).

Previously, we showed that SERHL OE suppressed AKT/mTOR pathway activation, thereby inhibiting the metastatic ability of GC cells. Subsequently, we investigated the potential involvement of SERHL and KLF16 in AKT/mTOR signaling. Western blotting elucidated that SERHL and KLF16 plasmid co-transfection reactivated AKT/mTOR signaling, which had been inhibited by SERHL alone. In contrast, SERHL KD enhanced AKT/mTOR phosphorylation, whereas simultaneous silencing of both SERHL and KLF16 suppressed AKT/mTOR signaling (Fig. S5B1, S5B2). Notably, total AKT and mTOR protein levels remained unchanged.

To assess the KLF16 effect on SERHL-modulated tumor progression *in vivo*, we injected AGS/SERHL, AGS/SERHL/KLF16, and control cells into the tail vein of nude mice (Fig. 4D1), revealing that the metastatic nodule area was significantly diminished in mice injected with SERHL-OE cells compared with controls, while SERHL and KLF16 co-OE cells elevated the percentage of fluorescent metastasis nodules area compared with SERHL-OE cells (Fig. 4D2). Histological investigation was utilized to verify the presence of metastases from GC to the lung (Fig. 4E). Employing immunohistochemistry, we examined cell metastasis marker (MMP2) expression. SERHL-OE cells had significantly decreased MMP2 levels compared with controls, whereas SERHL and KLF16 co-OE cells had increased MMP2 levels compared with SERHL-OE cells in GC lung metastases (Fig. 4E). Therefore, SERHL–KLF16 axis regulated GC progression and metastasis both *in vitro* and *in vivo*.

### KLF16 activates PRKCA and PLCB1 transcription

**Figure 5:**
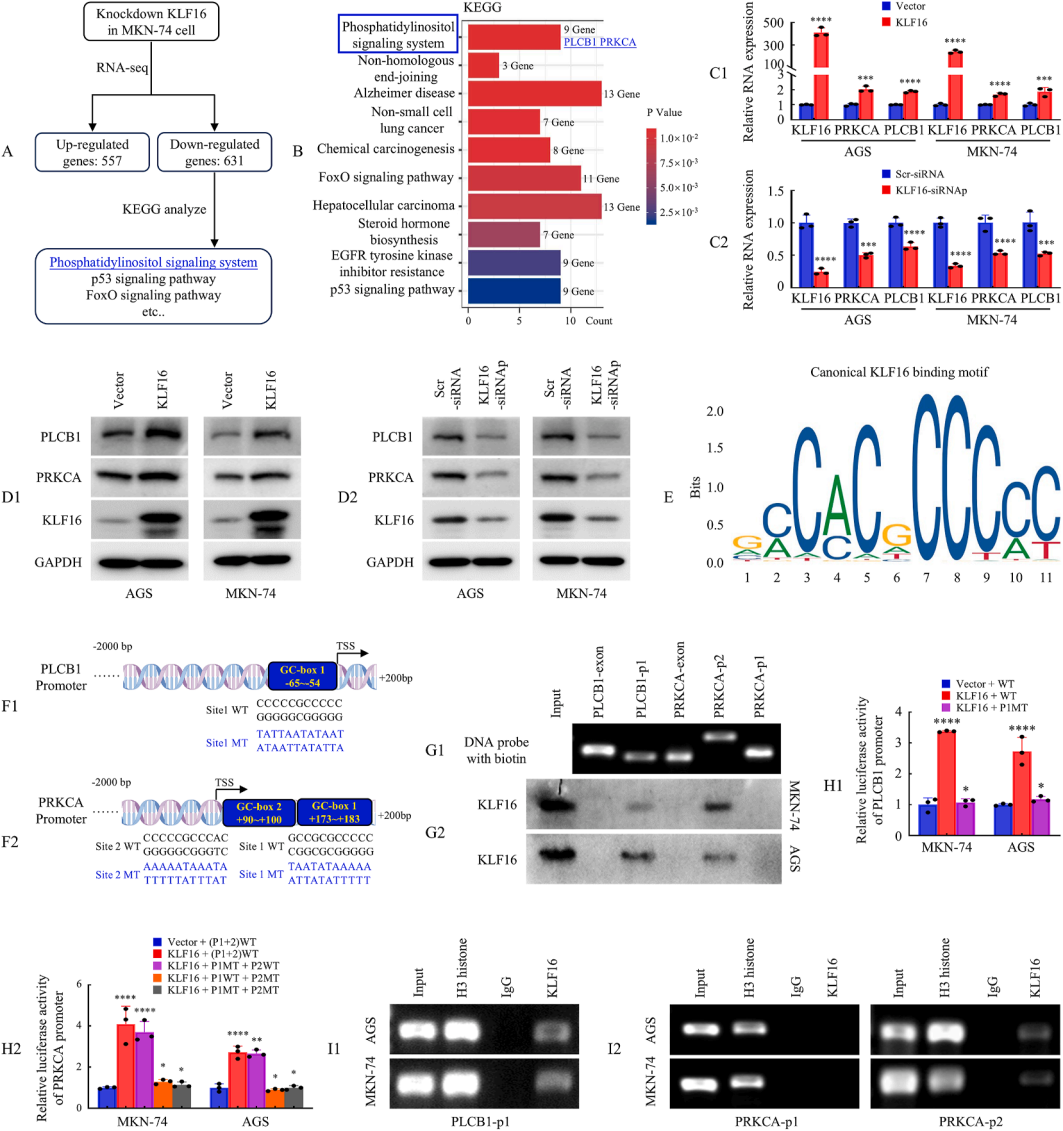
PLCB1 and PRKCA were direct transcript targets of KLF16. **(A)** The flow chart for down-regulated KLF16 in gastric cancer (GC) MKN-74 cells. **(B)** KEGG pathway analysis identified significantly enriched genes, including PLCB1 and PRKCA, in the phosphatidylinositol (PI) signaling pathway. **(C)** After KLF16 overexpression (C1) or siRNA knockdown (C2) in AGS and MKN-74 cells, KLF16, PLCB1, and PRKCA expression were assessed with quantitative PCR. ∗∗∗*P* < 0.01 and ∗∗∗∗*P* < 0.001 for Vector *vs*. KLF16 or Scr-siRNA *vs*. KLF16-siRNAp. **(D1/2)** AGS and MKN-74 cells were transfected with either a KLF16 plasmid (D1) or KLF16-siRNAp (D2), and Western blotting was used to measure the expression of KLF16, PLCB1, and PRKCA. **(E)** Informatics analysis predicted the binding motif for the transcription factor KLF16. **(F1/2)** Schematic illustration of the PLCB1 (F1) and PRKCA (F2) promoters with potential GC-box sites. TSS, transcription start site. WT, wild-type; MT, mutated. **(G1/2)** DNA probes were validated through nucleic acid electrophoresis (G1), and biotin-labeled DNA pulldown experiments were used to confirm the binding relationship between KLF16 and PLCB1 promoter or PRKCA promoter (G2). **(H1/2)** A luciferase reporter assay evaluated the impact of KLF16 on PLCB1 (H1) and PRKCA (H2) promoter sites in GC cells co-transfected for 48 h, with results shown as the ratio of promoter to control vector luciferase activity. RLU, relative luciferase units; P1, promoter site 1; P2, promoter site 2. ∗*P* > 0.05, ∗∗*P* < 0.05, and ∗∗∗∗*P* < 0.001. **(I1/2)** Chromatin immunoprecipitation-PCR confirmed the direct binding of KLF16 to the PLCB1-p1 (I1) or PRKCA-p2 (I2) in GC cells.

KLF16 is a transcription factor that works by regulating its downstream gene expression; accordingly, we performed bulk RNA sequencing of KLF16-shRNA or Scr-shRNA-transfected MKN-74 cells. RNAi-mediated repression of KLF16 in MKN-74 cells significantly altered the expression of thousands of protein-coding genes, which included the down-regulation of 631 genes and up-regulation of 557 genes above the threshold level (1.2-FC; Tables S6 and S7). As KLF16 acts as an oncogene, we selected 631 genes that were suppressed in KLF16-shRNA MKN-74 cells for further study (Fig. 5A). These target genes were subsequently subjected to KEGG analysis via the WebGestalt database (http://www.webgestalt.org/). The results revealed ten significantly enriched KEGG pathways, including the phosphatidylinositol signaling system (Fig. 5B; Fig. S6). Reports showed that nine phosphatidylinositol signaling genes containing CALM1, IMPA1, PI4K2A, PIK3C2A, PIP5K1B, PIK3R3, PIKFYVE, PLCB1, and PRKCA modulate many cellular functions, including cancer cell proliferation, invasion, and motility.^35^ Among these, four phosphatidylinositol signaling genes, including PIK3R3, PIKFYVE, PLCB1, and PRKCA, were previously found to be abnormally expressed in GC. Interestingly, PLCB1 may interact with PRKCA proteins based on HPRD databases (https://www.hprd.org/). Thus, we hypothesize that the expression of PLCB1 and PRKCA is regulated by KLF16 and contributes to GC progression.

Next, we estimated whether PLCB1 and PRKCA expression was affected by KLF16 OE or KD in GC cells, indicating that KLF16 OE significantly overexpressed PLCB1 and PRKCA (Fig. 5C1, D1), while KLF16 KD decreased PLCB1 and PRKCA in AGS and MKN-74 cells (Fig. 5C2, D2) via quantitative PCR and Western blotting.

KLF family members, functioning as transcription factors, have been shown to recognize GC-rich elements (Fig. 5E; GC-boxes: 5′-CACCC-3′).^36^^,^^37^ Subsequently, the JASPAR website was utilized (transcription factor score cutoff > 90%), aiming at predicting potential KLF16 binding regions on the PLCB1 (PLCB1p) and PRKCA (PRKCAp) promoters. Sequence analysis revealed one putative GC-box (−65 ∼ −54 bp, PLCB1-GC-box) in the PLCB1 promoter (Fig. 5F1) and two putative sites named GC-box 1 (+173 ∼ +183 bp, PRKCA-GC-box 1) and GC-box 2 (+90 ∼ +100 bp, PRKCA-GC-box 2) in the PRKCA promoter (Fig. 5F2). To assess whether KLF16 directly bound to the promoter of PLCB1 or PRKCA, we first performed DNA pulldown assays on the cell lysates. We synthesized biotin-labeled DNA probes via PCR and validated them through nucleic acid electrophoresis (Fig. 5G1). The DNA probes derived from the last exon (1000 bp) of PLCB1 and PRKCA were employed as negative controls. Results demonstrated that the promoter sequence of PLCB1-GC-box or PRKCA-GC-box 2 exhibited a binding affinity for KLF16 (Fig. 5G2). Moreover, KLF16 overexpression significantly increased the activity of PLCB1-GC-box and PRKCA-GC-box 2, but not mutant counterparts, as determined by luciferase reporter analysis (Fig. 5H1, H2). A chromatin immunoprecipitation assay further confirmed the direct interaction between KLF16 and the PLCB1-GC-box as well as between KLF16 and the PRKCA-GC-box 2 in GC cells (Fig. 5I1, I2).

Taken together, PLCB1 or PRKCA is a direct KLF16 transcriptional target in GC cells.

### KLF16 enhances GC invasion and metastasis by transactivating PLCB1 or PRKCA expression

**Figure 6:**
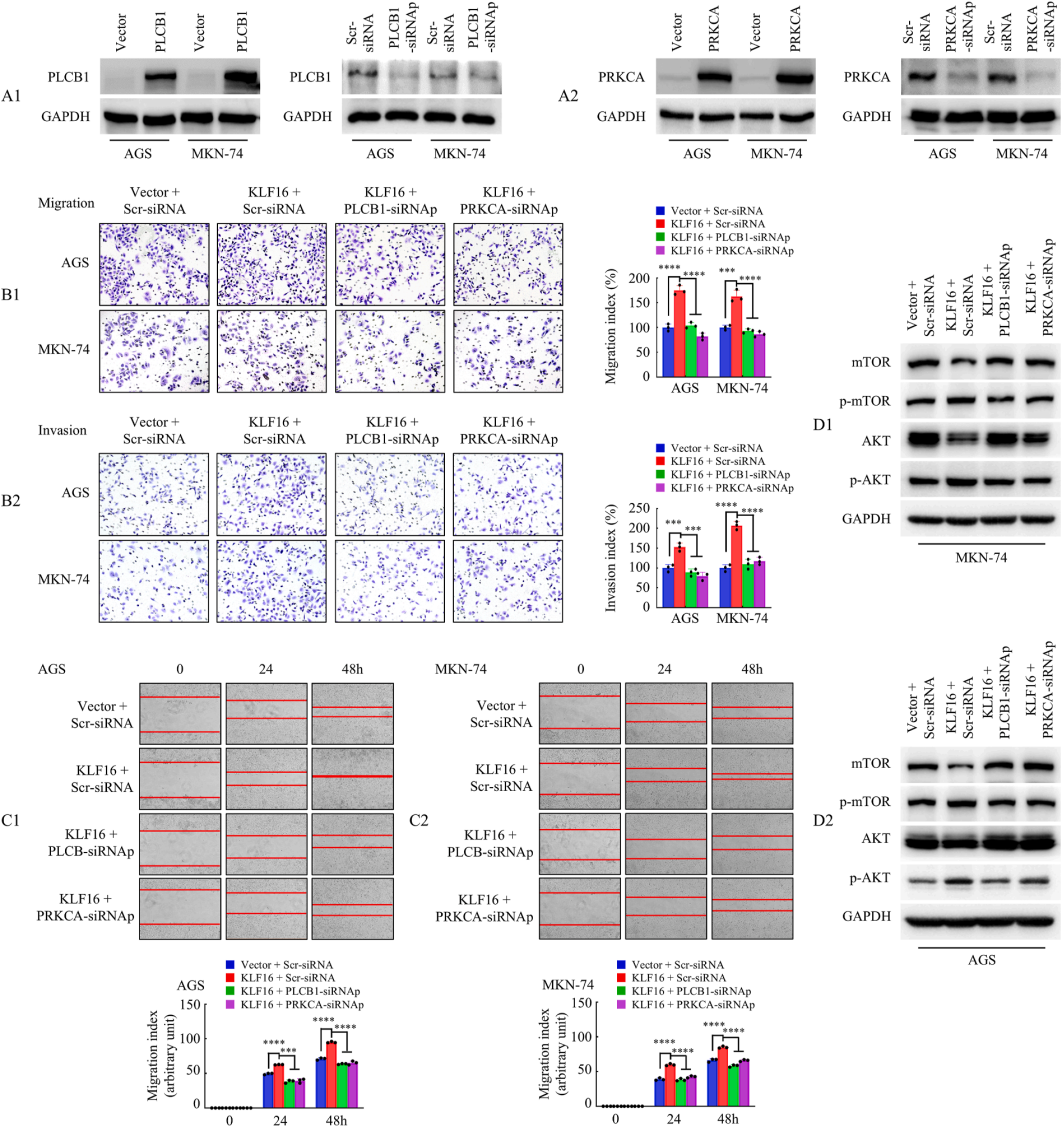
KLF16–PLCB1/PRKCA pathway promotes the invasion of gastric cancer (GC) cells *in vitro*. **(A1/2)** PLCB1 and PRKCA protein expression levels were analyzed in GC cells 48 h post-transfection. **(B1/2)** GC cell migration and invasion were assessed using a Transwell assay. ∗∗∗*P* < 0.01 and ∗∗∗∗*P* < 0.001. **(C1/2)** 2D cell migration was evaluated with a wound healing assay. ∗∗∗*P* < 0.01 and ∗∗∗∗*P* < 0.001. **(D1/2)** The analysis of AKT/mTOR signaling pathway among co-transfected KLF16 and PLCB1/PRKCA-siRNA groups of GC cells by Western blotting.

Next, we investigated whether PLCB1 and/or PRKCA mediated the effect of KLF16 on GC cell migration and invasion by constructing plasmids and designing siRNAs targeting PLCB1 or PRKCA with different sequences. Their efficacy was verified through Western blot analysis (Fig. 6A1, A2). Next, we transfected cells with Control + Scr-siRNA, KLF16 + Scr-siRNA, KLF16 + PLCB1-siRNAp, and KLF16 + PRKCA-siRNAp groups. Moreover, we showed that KLF16 OE increased AGS and MKN-74 cell invasion and migration compared with the Vector cells; however, KD of PLCB1 or PRKCA in KLF16 presence decreased GC cell invasion and migration compared with the KLF16 + Scr-siRNA group (Fig. 6B1, B2). Similarly, wound healing assays demonstrated that the KLF16 OE-induced metastatic phenotypes were abolished by the repression of PLCB1 or PRKCA (Fig. 6C1, C2).

Previous results have confirmed that the SERHL–KLF16 axis regulates AKT/mTOR signaling. We then explored the impact of the KLF16-PLCB1/PRKCA axis on this pathway. Western blot analysis revealed that KLF16 OE, while interfering with PLCB1/PRKCA, suppressed the KLF16-induced activation of the AKT/mTOR pathway (Fig. 6D1, D2).

Overall, KLF16 activated the AKT/mTOR pathway and enhanced tumor invasion through PLCB1/PRKCA.

### SERHL suppressed invasion and metastasis through KLF16-PLCB1/PRKCA signaling in GC cells

**Figure 7:**
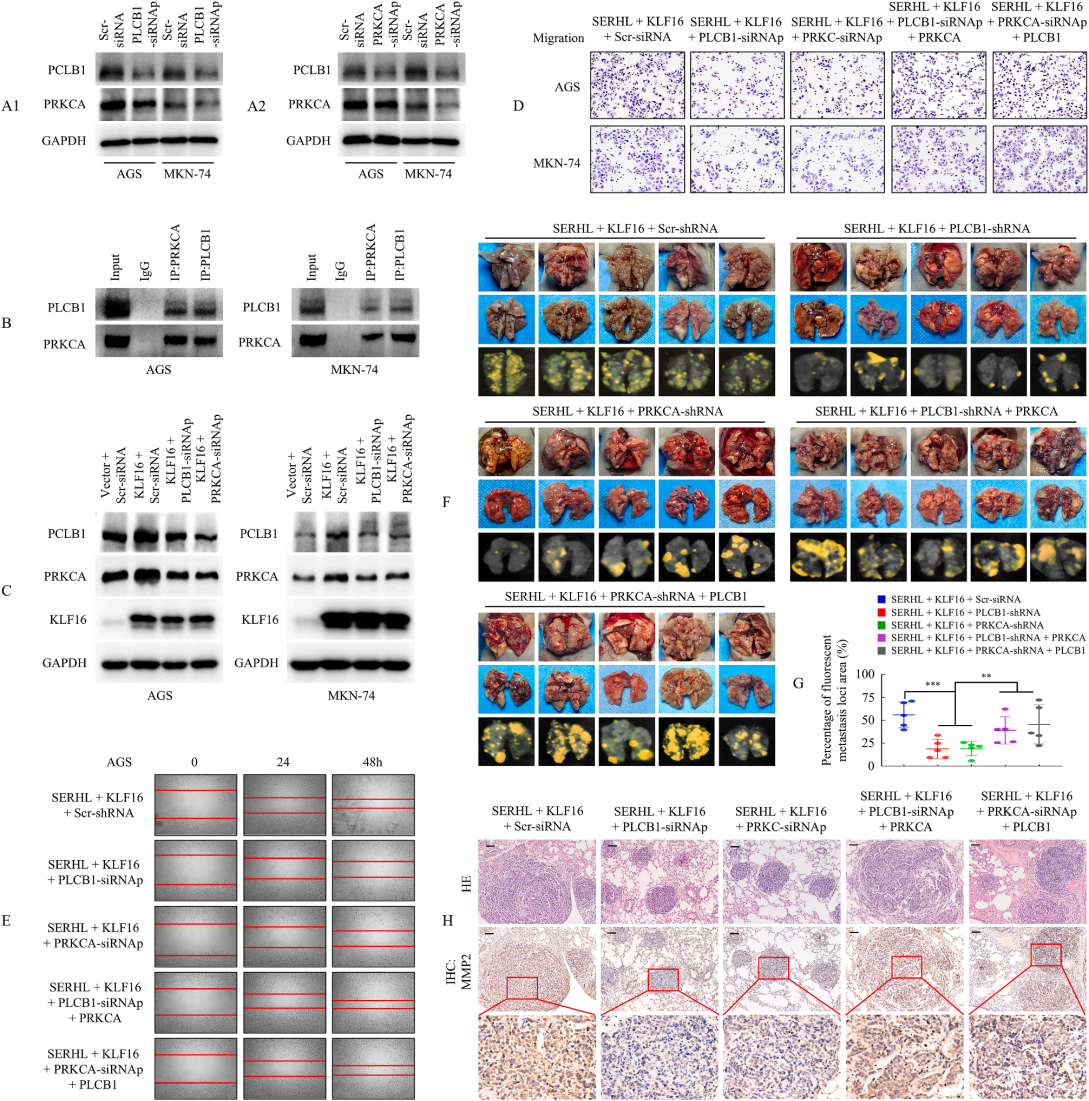
SERHL9 modulates invasion and metastasis through KLF16–PLCB1/PRKCA signaling in gastric cancer (GC) cells. **(A1/2)** AGS and MKN-74 cells were transfected with Scr-siRNA and either PRKCA-siRNAp or PLCB1-siRNAp for 48 h, and PLCB1 and PRKCA levels were analyzed by Western blotting. **(B)** Co-immunoprecipitation assays evaluated the endogenous interaction between PLCB1 and PRKCA in AGS and MKN-74 cells. **(C)** GC cells were co-transfected with Vector + Src-siRNA, KLF16 + Src-siRNA, KLF16 + PLCB1-siRNAp, and KLF16 + PRKCA-siRNAp. The KLF16, PLCB1, and PRKCA expression were measured by Western blotting. **(D)** Transwell migration assays were performed on GC cells. **(E)***In vitro* scratch wound healing assay in AGS cells. **(F, G)** Lung samples were collected from mice injected with AGS cells via the tail vein. The image shows lung metastatic nodules (F) and the percentage of fluorescent metastatic areas (G). ∗∗*P* < 0.05 and ∗∗∗*P* < 0.01. **(H)** Hematoxylin-eosin staining and MMP2 immunohistochemical staining were conducted on lung samples from these mice. Scale bar, 100 μm.

According to HPRD databases, PLCB1 may bind to PRKCA proteins. First, we showed that PLCB1 depletion significantly down-regulated PRKCA expression, whereas PRKCA depletion significantly reduced PLCB1 expression in AGS and MKN-74 cells by Western blot analysis (Fig. 7A1, A2). Then, we verified the interaction between endogenous PLCB1 and PRKCA protein *in vivo*. Whole-cell lysates from AGS and MKN-74 cells were prepared for immunoprecipitation. Indeed, PLCB1 and PRKCA could bind to each other, as confirmed by reciprocal co-immunoprecipitation experiments (Fig. 7B).

Third, we determined whether PLCB1 or PRKCA protein was involved in KLF16-mediated expression, observing that exogenous KLF16 expression up-regulated PLCB1 and PRKCA compared with the Vector cell group, while co-transfection KLF16 and PLCB1-shRNAp or PRKCA-shRNAp reversed the increasing trend compared with KLF16 in GC cells using Western blotting assay (Fig. 7C), suggesting that the relation between PLCB1 and PRKCA could influence KLF16-driven expression changes in these genes.

Fourth, we hypothesized that SERHL9 regulated PLCB1/PRKCA expression via KLF16 in GC cells. Accordingly, we designed five groups: group A, SERHL-KLF16-Scr-siRNA; group B, SERHL-KLF16-PLCB1-siRNAp; group C, SERHL-KLF16-PRKCA-siRNAp; group D, SERHL-KLF16-PLCB1-siRNAp-PRKCA; and group E, SERHL-KLF16-PRKCA-siRNAp-PLCB1. After co-transfection with GC cells for 48 h, group B or group C led to suppressed migration/invasion capacity compared with group A in GC cells. Nevertheless, the reintroduction of PRKCA (group D) or PLCB1 (group E) incompletely reversed the suppression compared with group B or group C in cells via Transwell and wound healing assays (Fig. 7D and E; Fig. S7A–S7D).

Next, we examined the function of the SERHL–KLF16–PLCB1/PRKCA pathway on tumor progression *in vivo* by constructing tail-vein metastasis using AGS cells in nude mice, thereby causing lung metastases (Fig. 7F). Notably, group B (Lv-SERHL-KLF16-PLCB1-shRNA) or group C (Lv-SERHL-KLF16-PRKCA-shRNA) transduction in AGS cells significantly decreased the percentage of observable metastatic tumor area compared with group A (Lv-SERHL-KLF16-Scr-shRNA) (Fig. 7G). In contrast, group D (Lv-SERHL-KLF16-PLCB1-shRNA-PRKCA) or group E (Lv-SERHL-KLF16-PRKCA-shRNA-PLCB1) in tumors derived from GC cells formed a more metastatic nodule area than group B or group C (Fig. 7G). The GC metastases in the lungs of nude mice were further confirmed through hematoxylin-eosin staining**,** and the difference in cell motility ability was detected by MMP2 immunohistochemical staining (Fig. 7H; Fig. S7E).

Moreover, the results demonstrated that the inhibitory effect of SERHL on the AKT/mTOR pathway could be reversed by overexpressing PLCB1/PRKCA in GC cells (Fig. S7F).

Therefore, SERHL inhibited KLF16, thereby modulating PLCB1/PRKCA signaling in GC cells, suppressing the AKT/mTOR pathway, and suppressing invasion and metastasis.

### SERHL was negatively related to KLF16, PLCB1, and PRKCA expression in GC specimens

**Figure 8:**
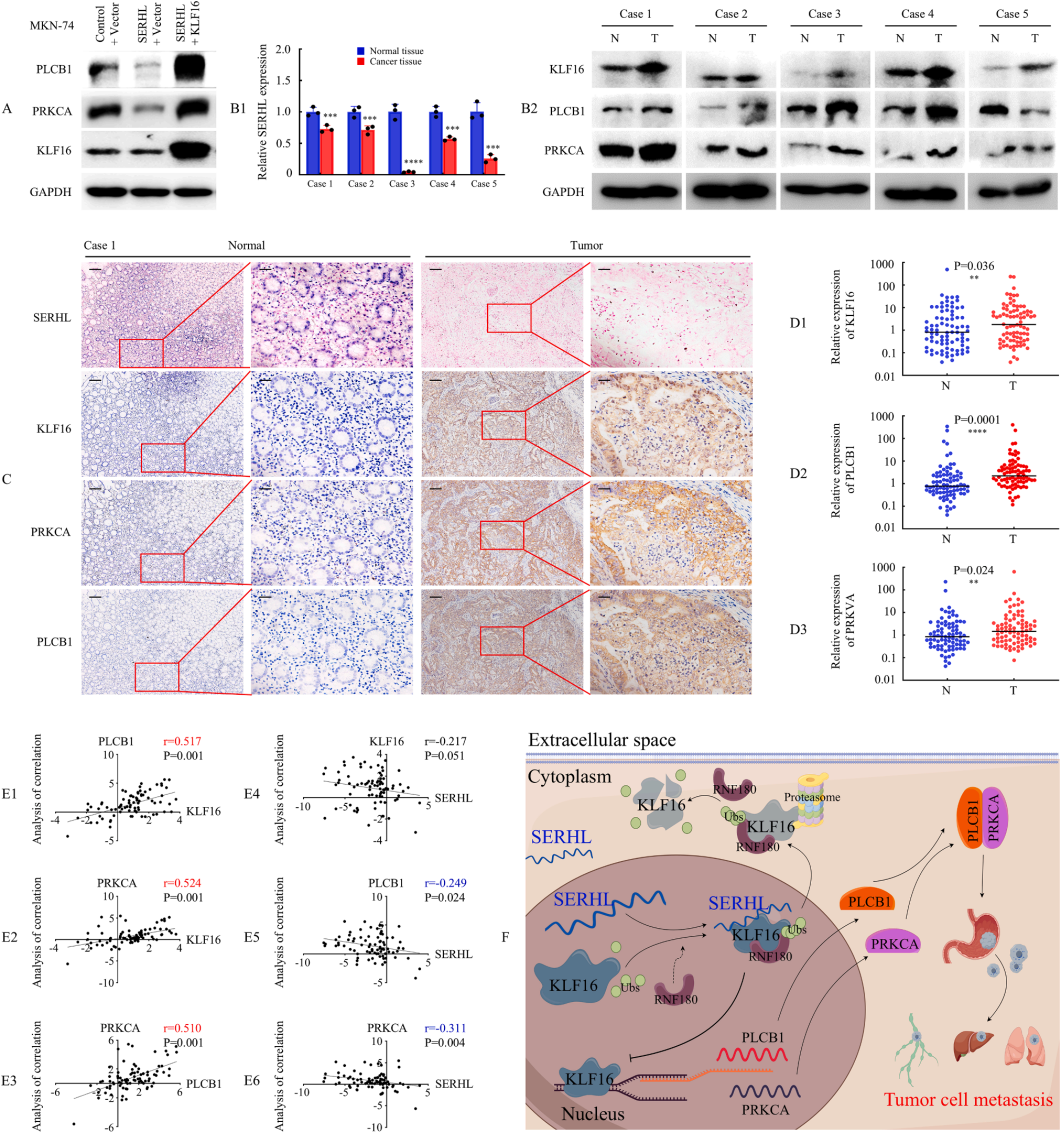
Inverse correlations of SERHL with KLF16, PLCB1, and PRKCA expression in gastric cancer (GC) tissues. **(A)** KLF16, PLCB1, and PRKCA expression were analyzed by Western blotting in MKN-74 cells co-transfected with Control/Vector, SERHL/Vector, and SERHL/KLF16. **(B1/2)** In five freshly obtained GC tissue samples, the expression levels of SERHL (B1), KLF16, PLCB1, and PRKCA (B2) were assessed by quantitative PCR (B1) or Western blotting (B2), normalized to B2M or GAPDH. ∗∗∗*P* < 0.01 and ∗∗∗∗*P* < 0.001 for normal *vs*. cancer. **(C)***In situ* hybridization and immunohistochemistry were performed to determine SERHL, KLF16, PLCB1, and PRKCA expression patterns in ten GC tissues and corresponding normal tissues. Scale bars, 100 μm. **(D)** In 82 GC tissue samples, the levels of KLF16 (D1), PLCB1 (B2), and PRKCA (D3) were measured using quantitative PCR and displayed in a scatter plot. ∗∗*P* < 0.05 and ∗∗∗∗*P* < 0.001 for Tumor (T) *vs*. Normal (N). **(E)** Correlations between KLF16 and PLCB1 expression (E1), between KLF16 and PRKCA expression (E2), between PLCB1 and PRKCA expression (E3), between SERHL and KLF16 expression (E4), between SERHL and PLCB1 expression (E5), or between SERHL and PRKCA expression (E6) in 82 GC tissues by quantitative PCR. **(F)** Mechanistic diagram for the effect of SERHL–KLF16–PLCB1/PRKCA pathway on GC cell invasion.

Finally, we determined whether SERHL regulated KLF16-mediated PLCB1 or PRKCA expression in AGS and MKN-74 cells, indicating that SERHL OE significantly down-regulated KLF16, PLCB1, and PRKCA, while SERHL and KLF16 OE reversed the suppression compared with control (Control + Vector) in GC cells (Fig. 8A; Fig. S8A). Moreover, SERHL KD markedly increased KLF16 and PLCB1/PRKCA expression, whereas SERHL and KLF16 KD reversed the enhancement compared with controls (Fig. S8B).

Then, we explored whether SERHL expression was associated with KLF16, PLCB1, or PRKCA expression in GC specimens obtained from GC patients to ascertain its clinical relevance. Both quantitative PCR and Western blot analyses showed that SERHL (9/10) was significantly down-regulated, while KLF16 (9/10), PLCB1 (8/10), and PRKCA (9/10) expression were significantly up-regulated (Fig. 8B; Fig. S8C). Furthermore, we conducted *in situ* hybridization and immunohistochemical assays and discovered that SERHL expression levels were frequently down-regulated, whereas KLF16, PLCB1, and PRKCA expression were usually up-regulated in the 10 tumor samples in contrast to the paired paracancerous tissues from the same patient (Fig. 8C; Fig. S8D; Table S8).

In addition, we investigated KLF16, PLCB1, or PRKCA expression in 82 pairs of human GC tissues and paired normal tissues via quantitative PCR, showing significantly increased KLF16 (Fig. 8D1), PLCB1 (Fig. 8D2), or PRKCA (Fig. 8D3) in GC tissues. Spearman's correlation analysis manifested positive correlations between KLF16, PLCB1, and PRKCA expression (Fig. 8E1–3) and a negative correlation of SERHL expression with PLCB1 (Fig. 8E5) or PRKCA (Fig. 8E6) expression. A negative trend between SERHL and KLF16 at the RNA level was observed (Fig. 8E4), although the difference was not significant (*P* = 0.051), There was an interplay between GC clinicopathological parameters and SERHL expression (Table S5). To explore whether SERHL–KLF16–PLCB1/PRKCA signaling was involved, we evaluated the relationship among KLF16 (Table S9), PLCB1 (Table S10), or PRKCA expression (Table S11) and the same clinicopathological parameters, revealing that they were significantly connected with tumor size, local invasion, TNM stage, and lymph node metastasis.

To separately discuss the impact of four molecules in signaling, a survival analysis was conducted in a cohort of GC patients. The results revealed no significant difference in survival between low and high SERHL expression levels, although low SERHL expression was associated with a trend toward reduced overall survival (Fig. S8E1). High expression of KLF16, PLCB1, and PRKCA was all associated with poor prognosis in the GSE15459 cohort (Fig. S8E2–S8E4).

Overall, the data indicate that SERHL expression levels are negatively correlated with the expression of KLF16, PLCB1, and PRKCA in GC patients.

## Discussion

The present study first identified SERHL as a tumor suppressor gene in GC. Additionally, down-regulated SERHL was related to GC malignant progression. Furthermore, SERHL OE significantly hampered GC proliferation and migration. Subsequently, SERHL interacted with KLF16 and promoted its degradation via the E3 ligase RNF180 to inhibit GC invasion and metastasis. In addition, KLF16 facilitated GC invasion and metastasis by transactivating PLCB1 or PRKCA expression. These data suggest that SERHL is frequently repressed in human GC, serving as a tumor suppressor via KLF16–PLCB1/PRKCA signaling.

Studies have shown that pseudogene-derived lncRNA dysregulation is linked to the development of multiple diseases.^38^ For instance, guanylate binding protein 1 pseudogene 1 (GBP1P1) suppresses influenza A virus replication by acting as a protein decoy for DExH-box helicase 9 (DHX9).^39^ Some pseudogene-derived lncRNAs contribute to tumor progression, serving as proto-oncogenes^40^ or tumor suppressor genes.^41^ For instance, the SUMO1P3 is up-regulated in GC and highlights poor prognosis for GC patients,^10^ and KRT19P3 impairs GC proliferation and metastasis via COPS7A-modulated NF-κB signaling.^11^ Nonetheless, the functionality of pseudogene-derived SERHL in human GC occurrence and development remains unclear. Here, SERHL was impeded in GC cells and tissues and exhibited a main nuclear localization. Moreover, its expression was negatively linked with tumor size, TNM stage, local invasion, and lymph node metastasis in patients with GC. Using gain and loss of SERHL function experiments, we elucidated that SERHL OE inhibited the aggressive behavior of GC proliferation, invasion, and metastasis, while its KD exerted opposite effects on these phenotypes. This is in line with prior results, wherein it is reported that pseudogene-derived surfactant-associated 1 (SFTA1P) suppresses cell growth, invasion, and motility in GC. Our present study revealed that SERHL may act as a tumor suppressor gene to suppress GC development and progression.

KLF16, a transcription factor containing a conserved zinc finger domain and a Krüppel-like factor (KLF) family member, contributes to various cancer progression. For instance, KLF16 is significantly overexpressed in GC tissues in contrast to adjacent normal tissues.^42^ In addition, it can govern transcription by binding to GC-rich DNA elements in a promoter-dependent manner. For example, KLF16 up-regulates magnesium transporter 1 (MAGT1) to govern breast cancer tumorigenesis and progression.^43^ Moreover, KLF16 transcriptionally represses B-cell lymphoma 2-like 15 (BCL2L15) by binding to its promoter in retinoblastoma cells.^44^ The protein-coding gene SCL/TAL1 interrupting locus (STIL) interacts with KLF16 proteins, thereby facilitating BC development.^45^ In addition, KLF16 interacts with the protein-coding genes, nucleophosmin 1 (NPM1) and fibrillarin (FBL), to inhibit colorectal cancer proliferation.^46^ Nevertheless, whether lncRNAs interact with KLF16 to modulate GC development and progression is undetermined. In this study, pseudogene-derived lncRNA-SERHL might interact with KLF16, verified via mass spectrometry, RNA pulldown, Western blot, and RNA immunoprecipitation assays, and regulate the expression of KLF16 protein. Moreover, SERHL promoted KLF16 protein degradation. Specifically, functional assays manifested that SERHL significantly impaired GC invasive ability, while KLF16 reversed SERHL-mediated GC motility suppression. This implies that the function of lncRNA-SERHL in GC is partly fulfilled by regulating downstream target proteins, a reported acting mode of lncRNAs. For example, WAP four-disulfide core domain 21, pseudogene (WFDC21P) directly binds to GTPase Ras-related nuclear protein and modulates GC invasion and motility.^47^ RNF180 functions as an E3 ubiquitin ligase and facilitates the ubiquitination of various substrate proteins.^34^^,^^48^ Therefore, our results suggest not only that SERHL could interact with KLF16 and promote its ubiquitination and degradation via E3 ligase RNF180 in GC but also SERHL–KLF16 axis governs GC progression both *in vitro* and *in vivo*.

The PLCB1 gene governs cell proliferation and migration in addition to having a cancer-promoting function in diverse tumors, including breast cancer, cholangiocarcinoma, and GC. Protein kinase C-α (PKCα), also termed PRKCA, is a protein kinase C family member. PKCα overexpression has been reported in various cancers, such as GC,^49^ breast cancer,^50^ and bladder cancer.^51^ Intriguingly, the regulation of PLCB1 and PRKCA transcription potentially involves transcription factors. For example, PLCB1 is a direct target of transcriptional activation by transcription factor early growth response-1 (Egr-1) and positively regulates Egr-1 expression in mammalian cells.^52^ Then, PKCα is a direct transcriptional target of transcription factor forkhead box C2 (FOXC2) and thereby facilitates migratory and invasive capabilities of breast cancer cells.^53^ However, the mechanism by which PLCB1 and PKCα are direct targets of transcriptional regulation by KLF16 to promote cell invasiveness needs further investigation. Herein, a positive interplay between PLCB1/PRKCA and KLF16 expression in GC cells was observed via RNA sequencing, quantitative PCR, and Western blot assays. Based on these assays, we analyzed the PLCB1 or PRKCA promoter via JASPAR software and identified GC-boxes, which comprise the nucleotide sequence 5′-CACCC-3′. More importantly, luciferase reporter, chromatin immunoprecipitation, and DNA pulldown assays showed the binding between KLF16 and PLCB1 or PRKCA promoter. Consistent with our results, KLF16 transcriptionally regulates mitochondrial transcription factor A (TFAM) expression in glioma cells.^54^ To validate whether PLCB1 or PRKCA contributed to the KLF16 oncogenic function in GC, we further knocked down PLCB1 or PRKCA in GC cells, revealing that PLCB1 KD or PRKCA KD in KLF16 overexpressing cells decreased their migratory and invasion potential *in vitro*. This suggests that KLF16 promotes GC invasion and metastasis by transactivating PLCB1 or PRKCA expression.

Prior research has indicated that the PLC/PKC signaling pathway, primarily involving PLCβ and PKCα, plays a crucial role in diverse biological processes, including tumorigenesis.^55^ For instance, treatment with thrombospondin-2 has been shown to activate PLCβ and PKCα, thereby overexpressing MMP-9 and enhancing migratory activity in human osteosarcoma cells.^56^ Additionally, PLCE1 has been found to trigger NF-κB signaling by up-regulating PKCα, thereby facilitating esophageal cancer cell invasion and migration.^57^ Our Western blotting results revealed a positive regulatory interaction between PLCB1 and PRKCA, and the co-immunoprecipitation experiment demonstrated their mutual binding. This relationship may represent a novel mechanism for PLC/PKC to regulate tumor metastasis. Subsequent functional assays further substantiated the role of the SERHL–KLF16–PLCB1/PRKCA pathway in the metastasis of GC cells. Furthermore, our investigation into the AKT/mTOR signaling pathway revealed that the SERHL–KLF16–PLCB1/PRKCA signaling may influence the metastasis of GC cells through its regulatory effects on AKT/mTOR signaling activation. These data indicate that the KLF16/PLCB1/PRKCA axis is a key downstream mechanism mediating the function of SERHL in GC.

To conclude, we identified a new SERHL pseudogene that can inhibit GC development both *in vivo* and *in vitro*. In-depth studies on the molecular mechanism showed that SERHL bound to KLF16 and heightened its degradation, thereby inhibiting the transcription of PLCB1/PRKCA facilitated by KLF16. Consequently, SERHL may have a therapeutic potential to repress GC metastasis by inhibiting KLF16-related PLCB1/PRKCA signaling.

## CRediT authorship contribution statement

**Linjie Hong:** Writing – original draft, Visualization, Validation, Methodology, Investigation, Formal analysis, Data curation. **Siyang Peng:** Methodology, Investigation. **Yidong Chen:** Methodology, Investigation. **Yanci Xie:** Methodology, Investigation. **Yuting Lei:** Methodology, Investigation. **Jieke Wu:** Resources, Methodology, Investigation. **Xiaodong Huang:** Resources, Methodology, Investigation. **Xiangyang Wei:** Methodology, Investigation, Formal analysis. **Ping Yang:** Validation, Methodology, Investigation. **Jieming Zhang:** Validation, Methodology, Investigation. **Qiong Yang:** Supervision, Project administration. **Weimei Tang:** Supervision, Project administration. **Xiaosheng Wu:** Supervision, Project administration. **Ying Peng:** Supervision, Project administration. **Zhizhao Lin:** Supervision, Project administration. **Aimin Li:** Supervision, Project administration. **Side Liu:** Supervision, Project administration, Funding acquisition. **Jiaying Li:** Visualization, Validation, Supervision, Funding acquisition, Formal analysis, Conceptualization. **Weiyu Dai:** Writing – review & editing, Writing – original draft, Visualization, Supervision, Funding acquisition, Conceptualization. **Jide Wang:** Writing – review & editing, Writing – original draft, Supervision, Funding acquisition, Conceptualization.

## Ethics declaration

The animal experimental protocols used in this study were approved by the Animal Ethics Committee of Nanfang Hospital, Southern Medical University (IACUC-LAC-20231008-005). Additionally, the research involving human tissues received ethical approval from the Ethics Committee of Nanfang Hospital (NFEC-2017-062).

## Date availability

All data are available within the article and Supplementary files, or from the authors upon reasonable request.

## Funding

This work was supported by the 10.13039/501100001809National Natural Science Foundation of China (No. 82273354, 82372955, 82073066, 82302891, 82403543), 10.13039/501100021171Guangdong Basic and Applied Basic Research Foundation (China) (No. 2024A1515012891), Science and Technology Program of Guangzhou, China (No. 2024A04J5133, 2025A04J3637), and the 10.13039/501100012245Science and Technology Planning Project of Guangdong Province, China (No. 2017B030314037).

## Conflict of interests

The authors declared no conflict of interests.
