# Supplementary material for: SERHL regulates invasion and metastasis through KLF16-PLCB1/PRKCA signaling in gastric cancer cells
Source: Genes Dis. 2025 Oct 20;13(6):101884. doi: 10.1016/j.gendis.2025.101884 (PMC13486994; doi:10.1016/j.gendis.2025.101884)

Fig.2F
Gel:8%

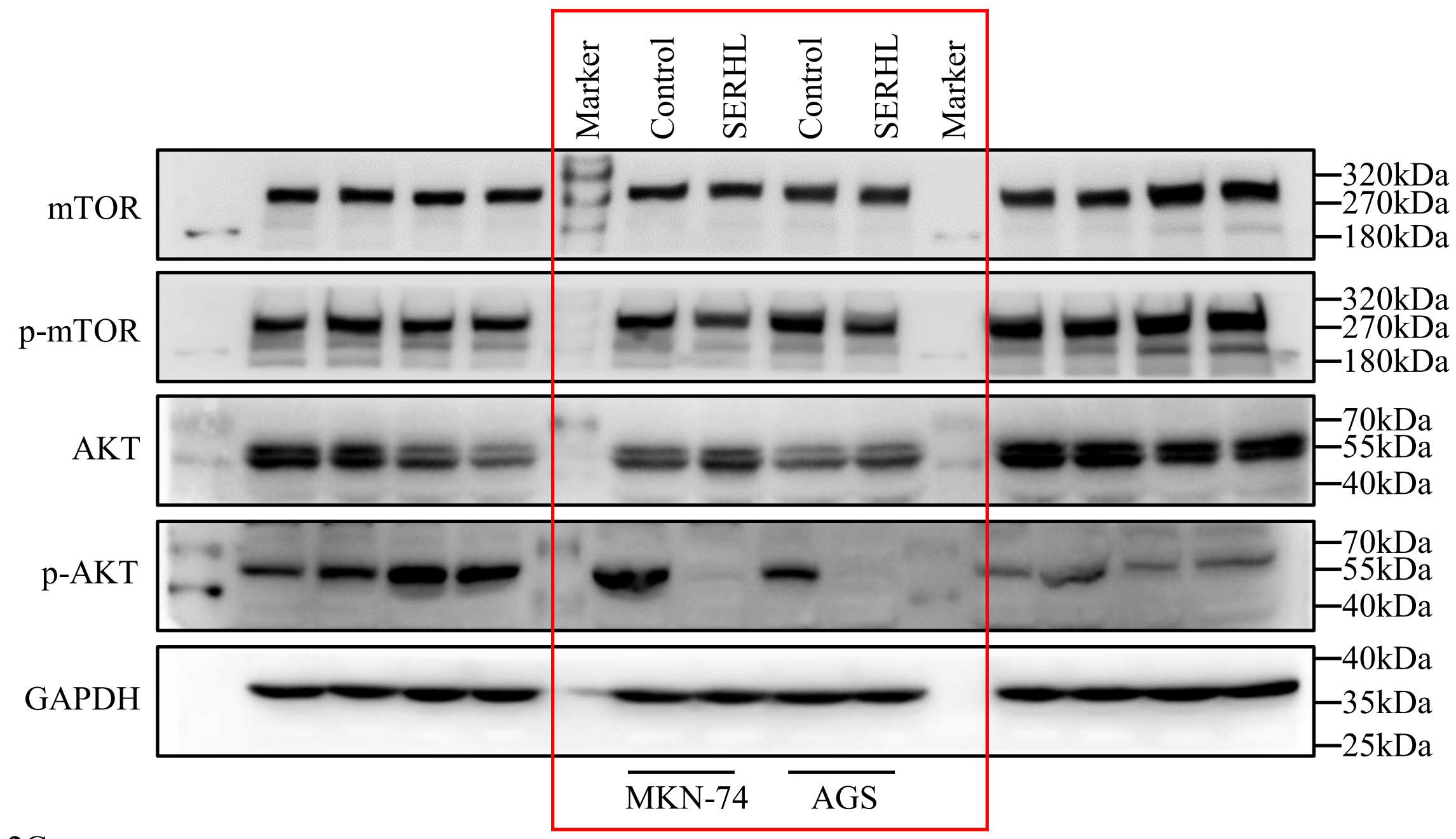

Fig.2G
Gel:8%

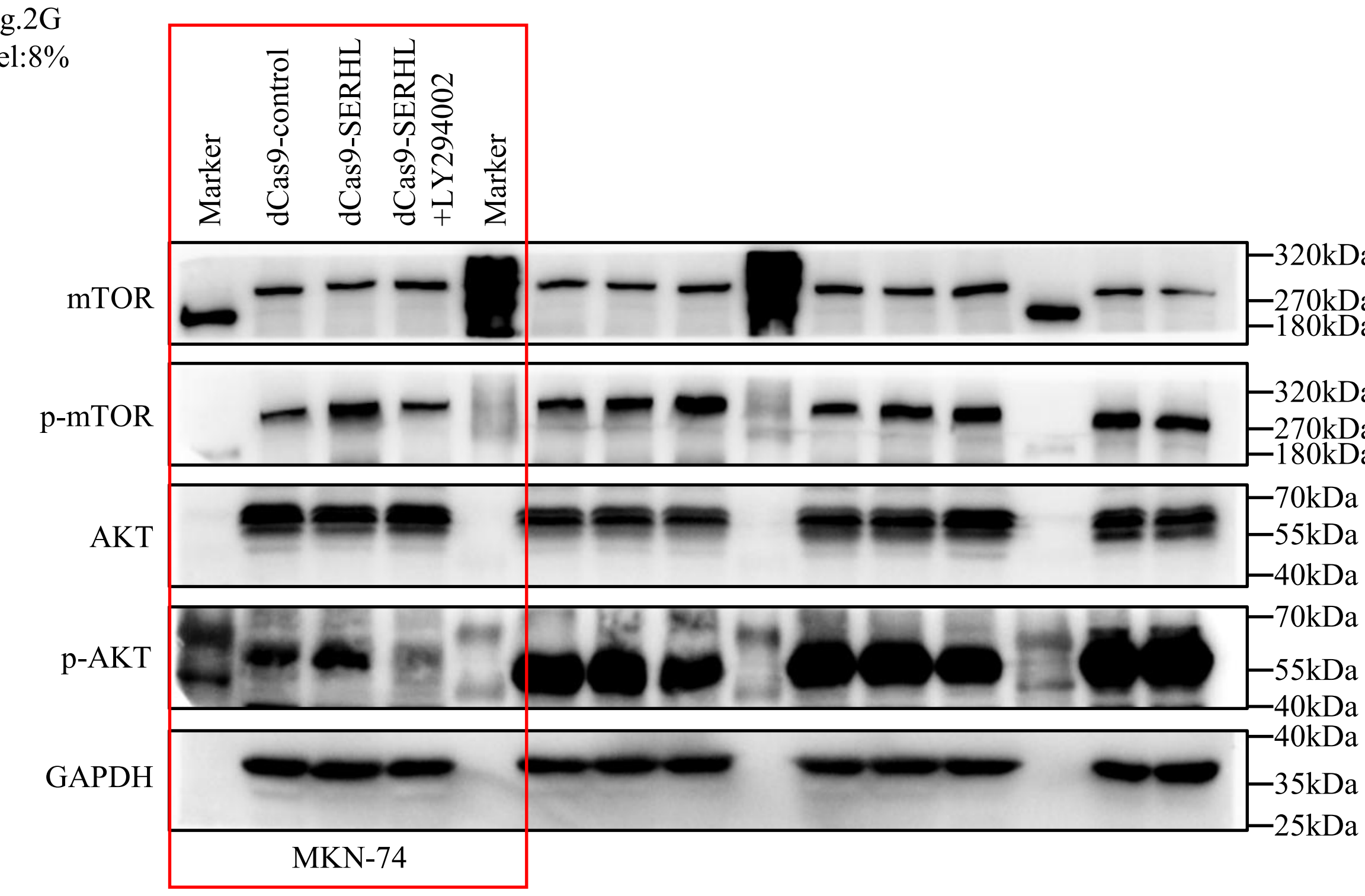

Fig.2G
Gel:8%

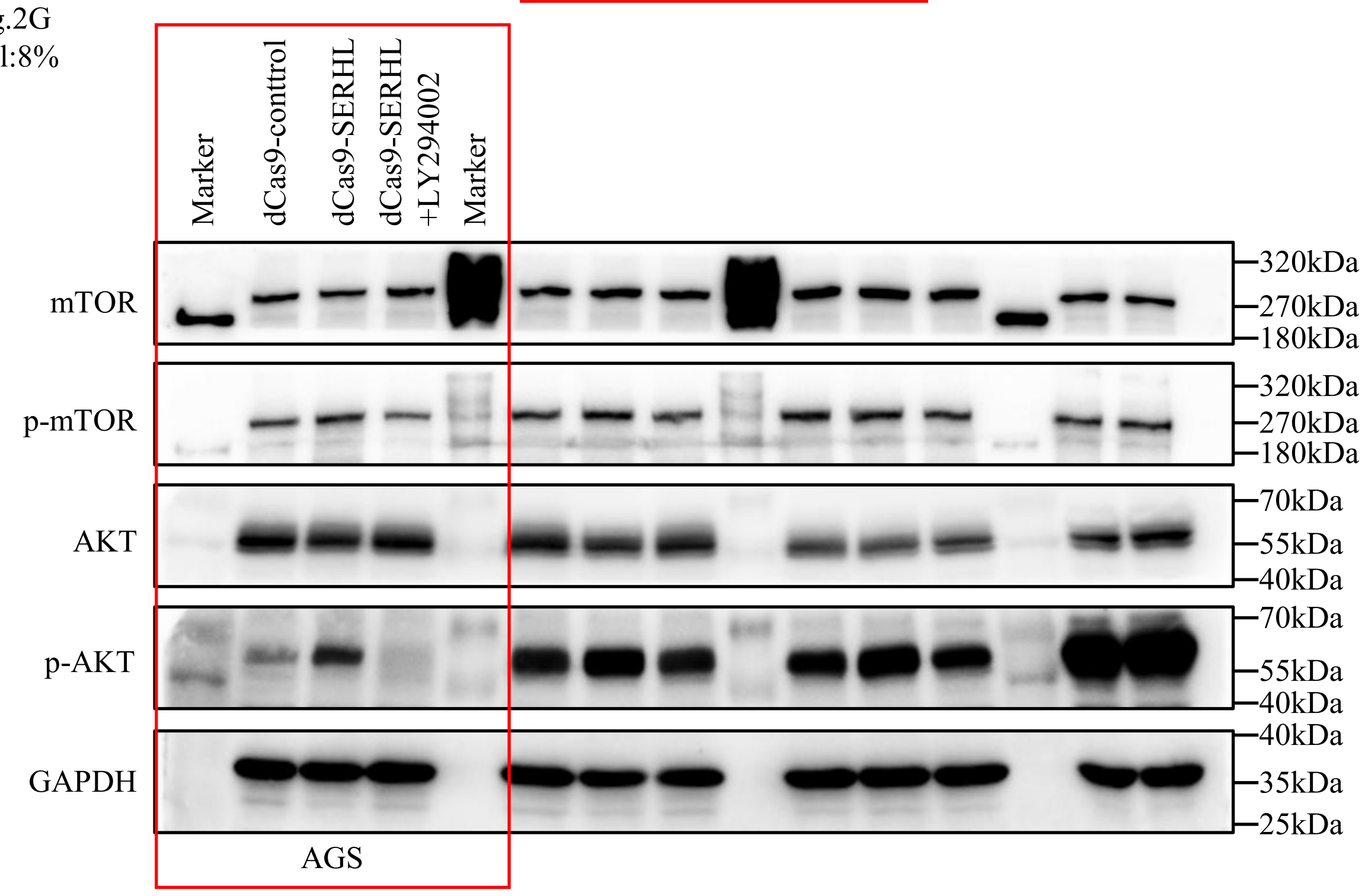

Fig.3G1&H1
Gel:10%

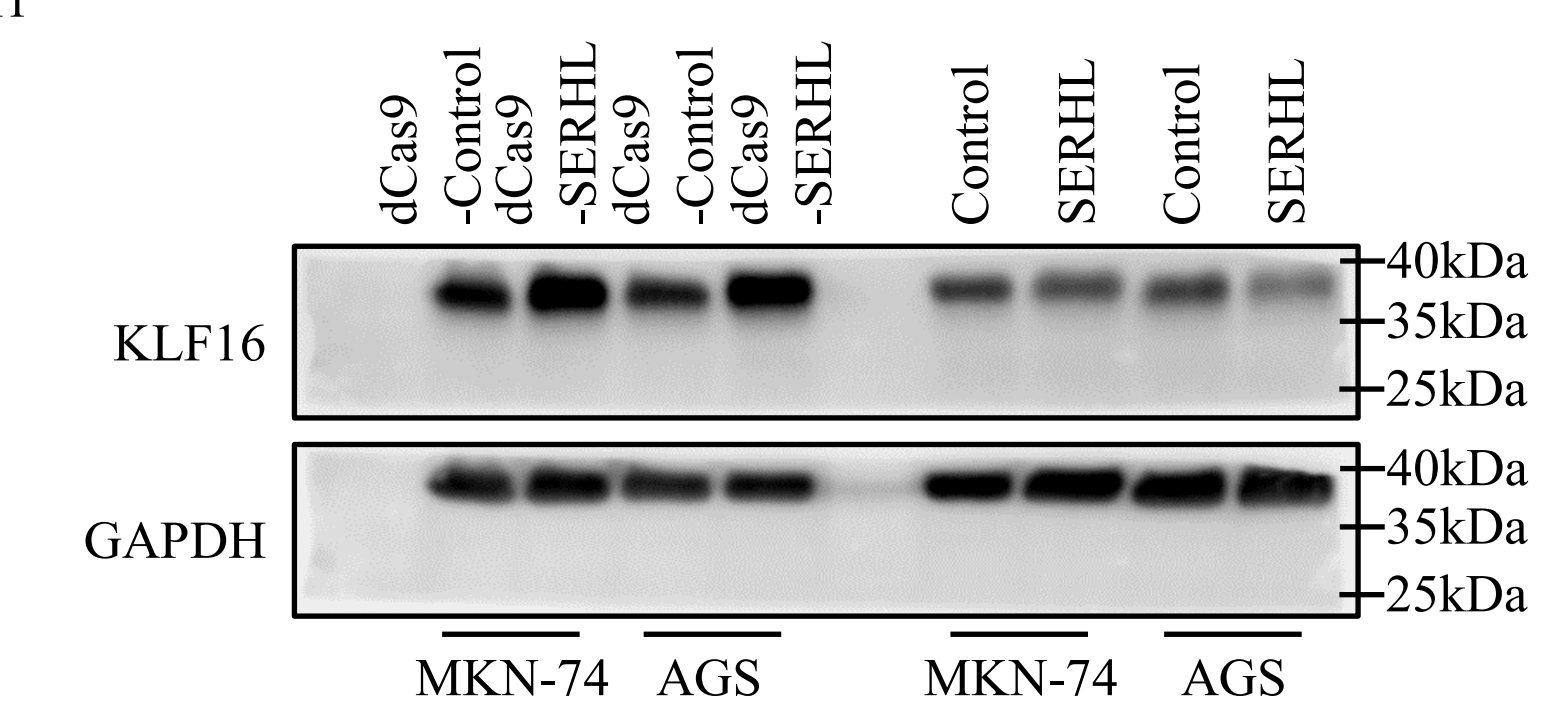

Fig.3I
Gel:10%

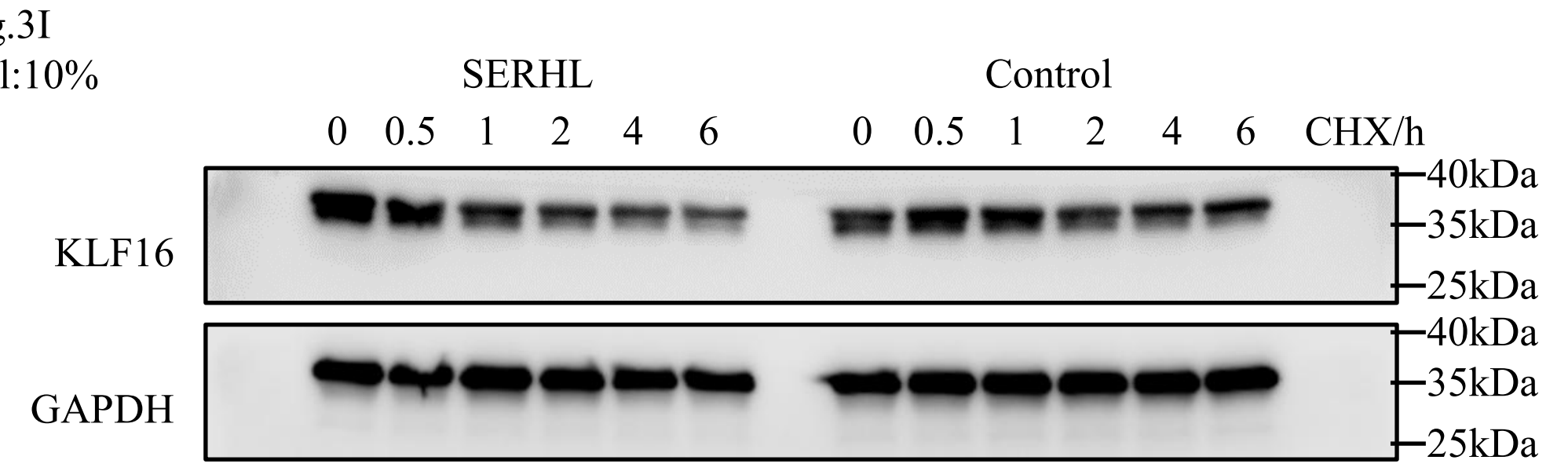

Fig.3A
Gel:1%

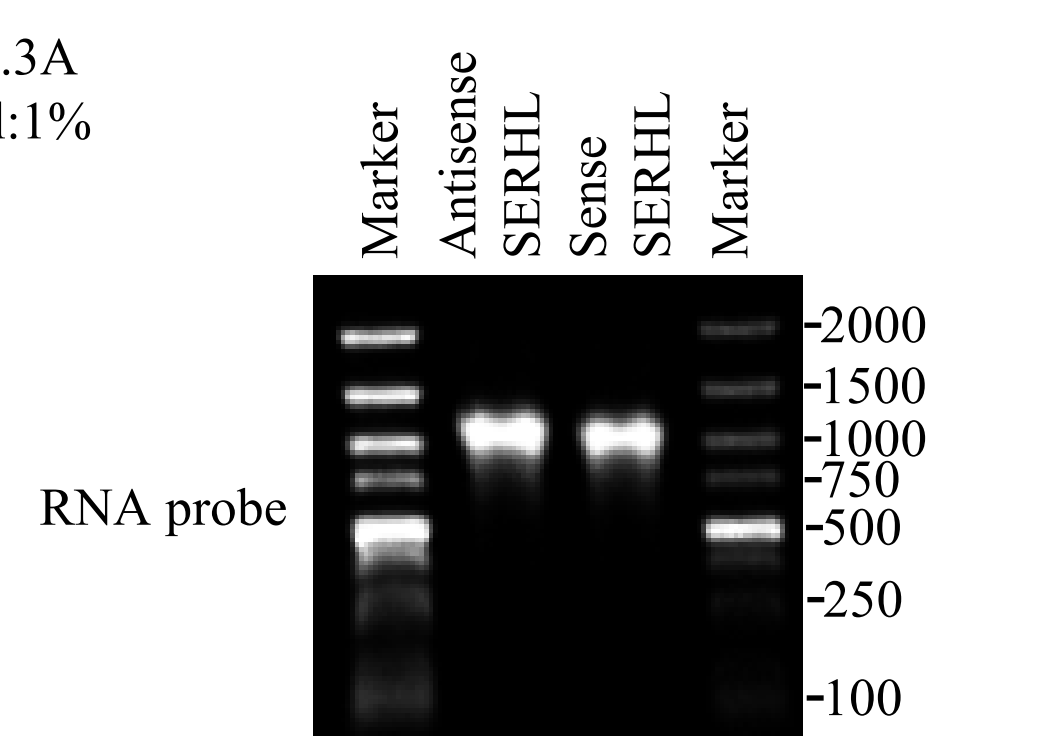

Fig.3B
Gel:10%

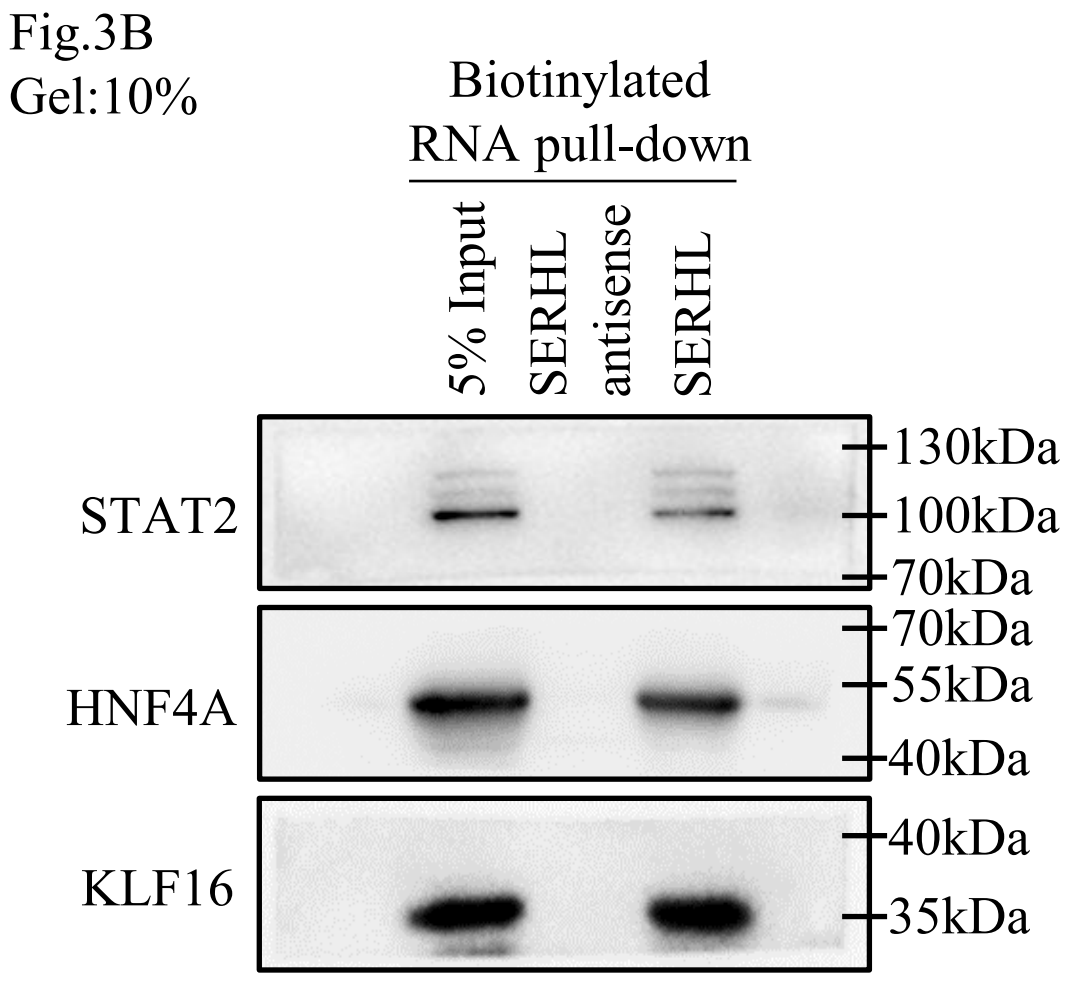

Fig.3J
Gel:10%

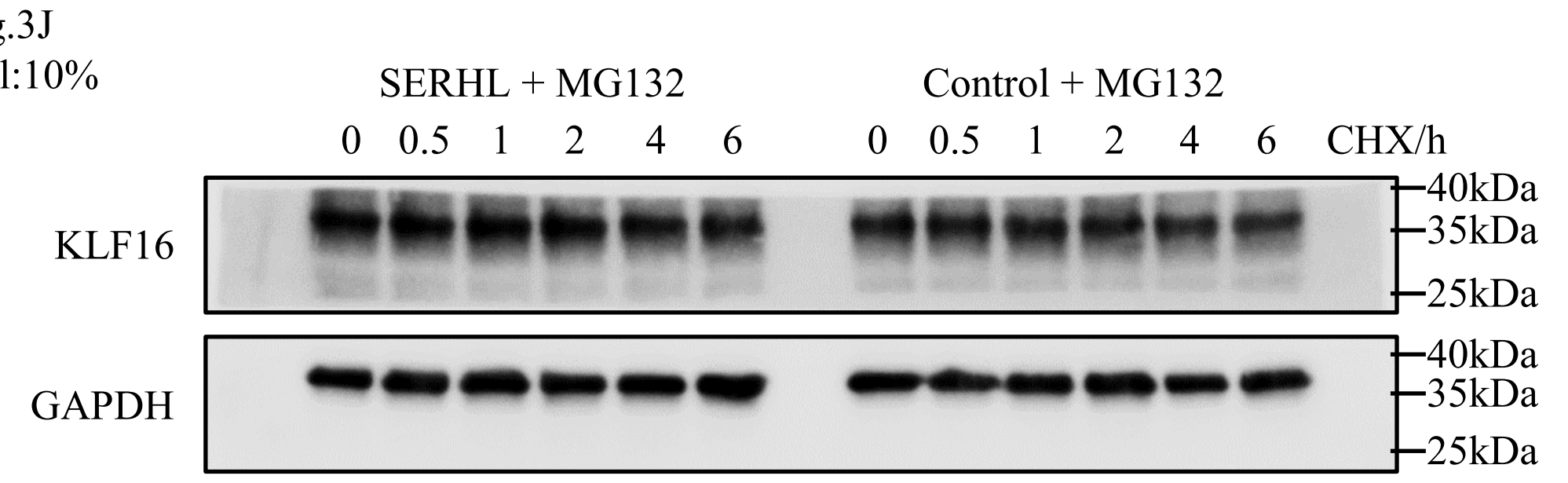

Fig.3D1
Gel:1%

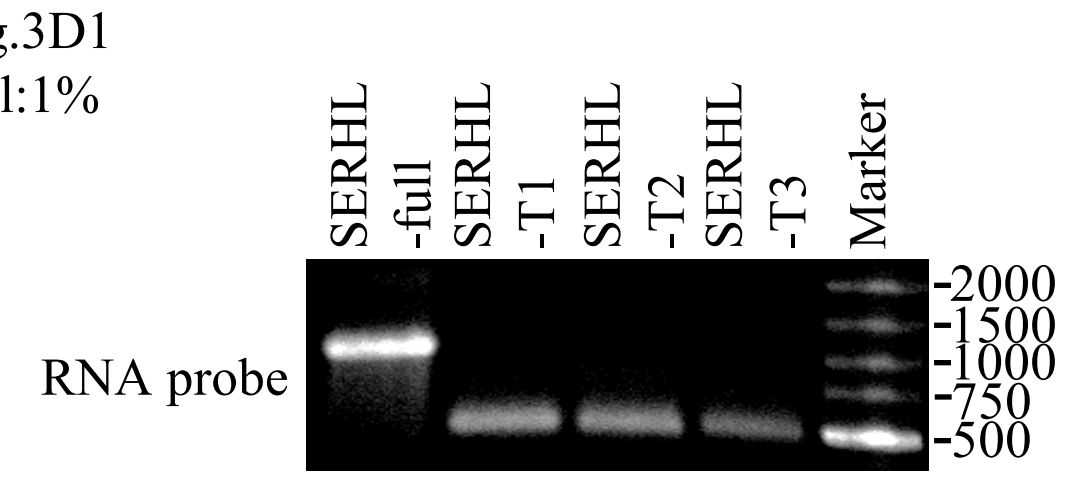

Fig.3K&L
Gel:10%

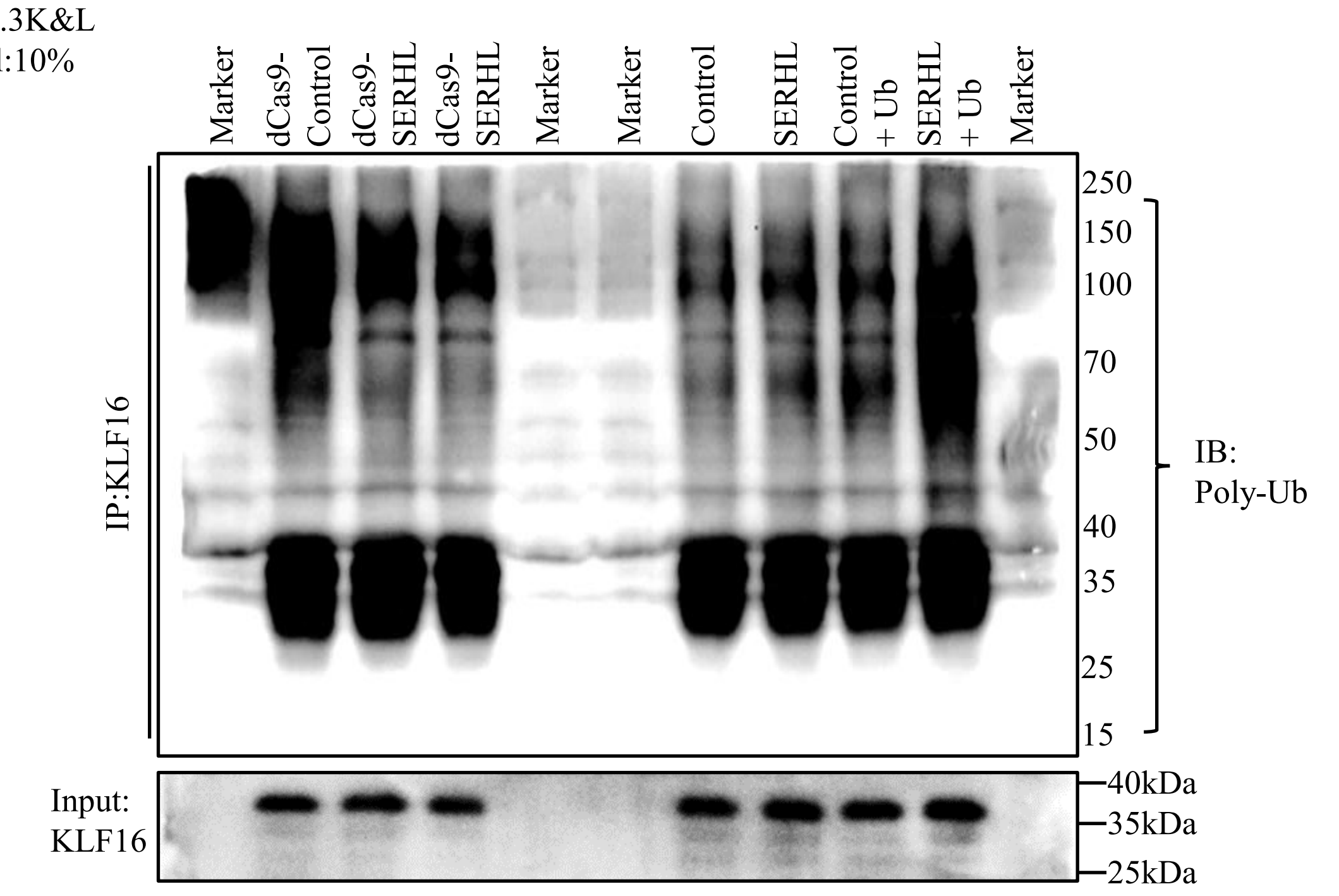

Fig.3D2
Gel:10%

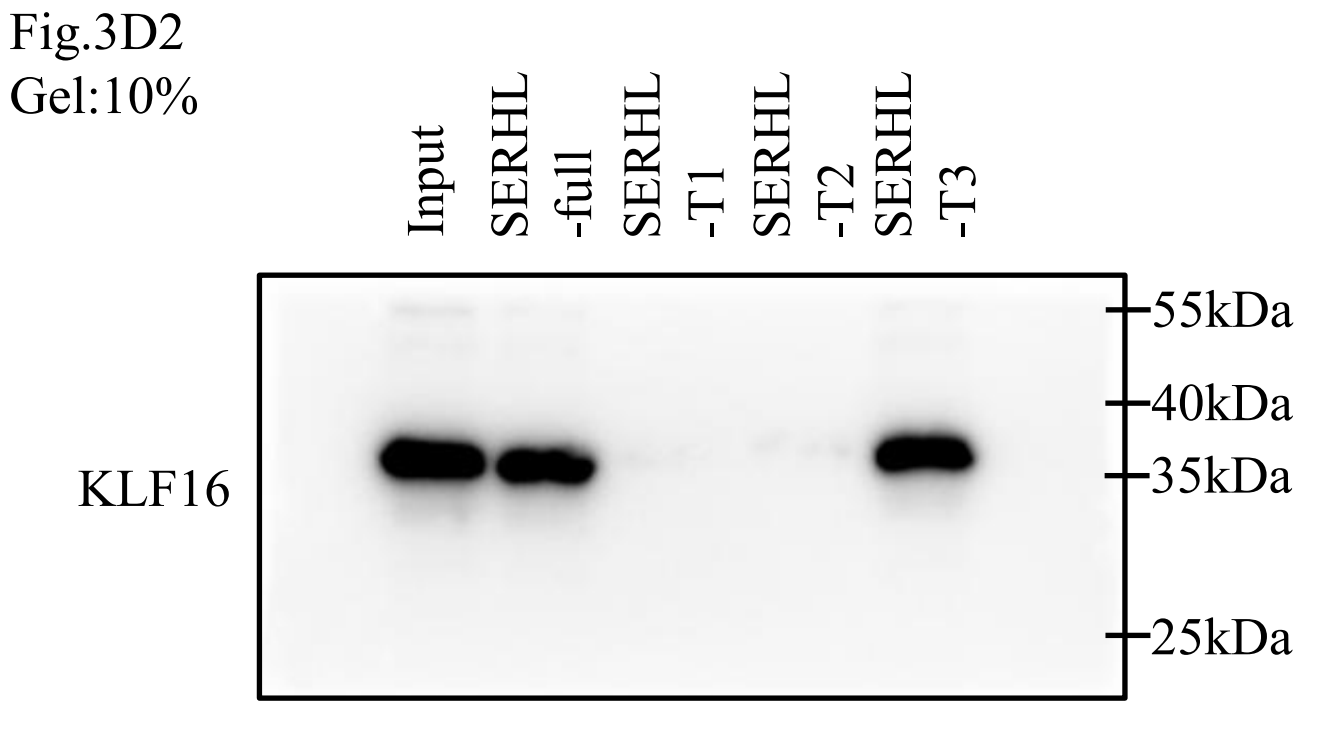

Fig.3E
Gel:12%

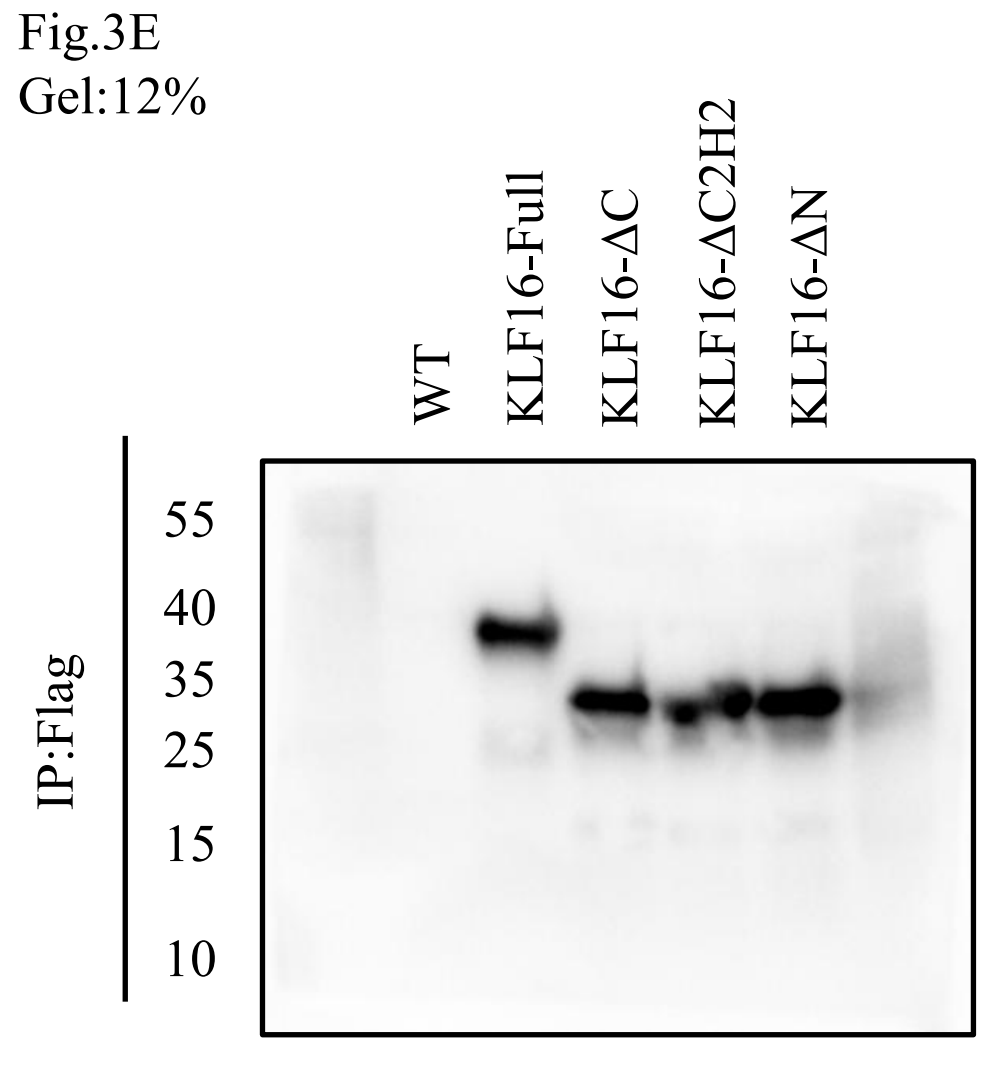

Supplementary Fig.4C1 Gel:10%

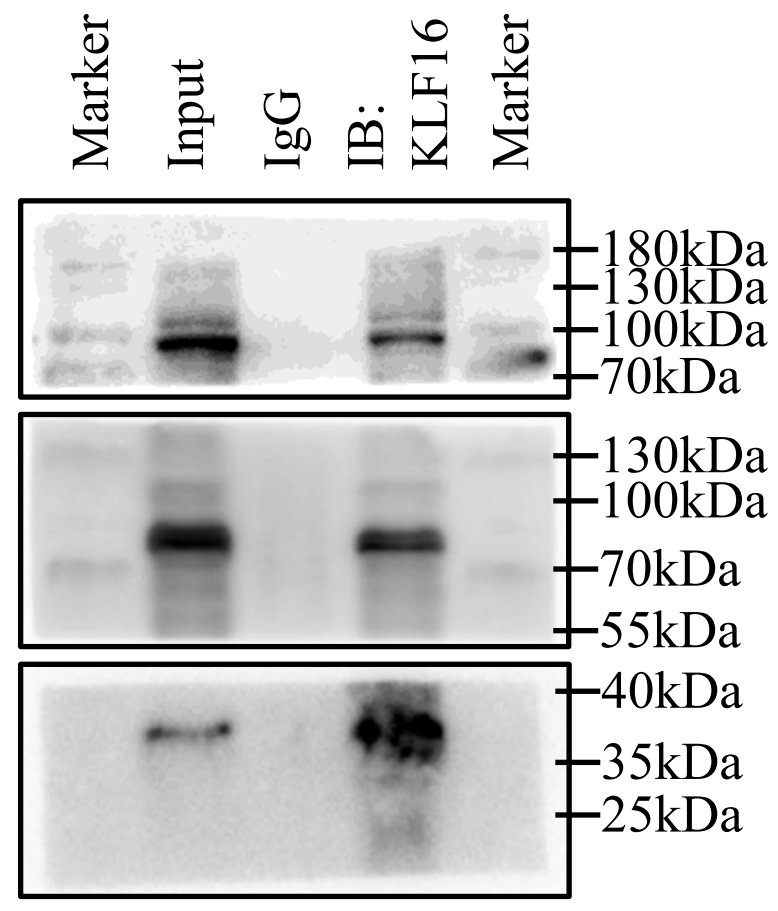

Supplementary Fig.4D Gel:10%

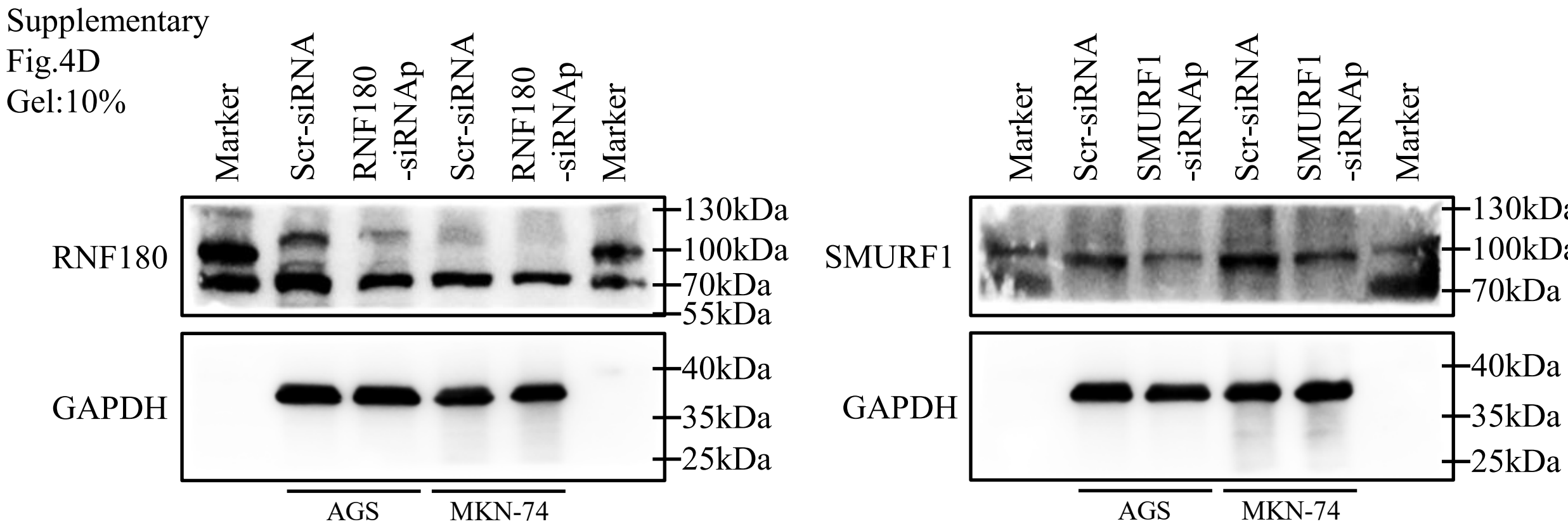

Supplementary Fig.4G Gel:10%

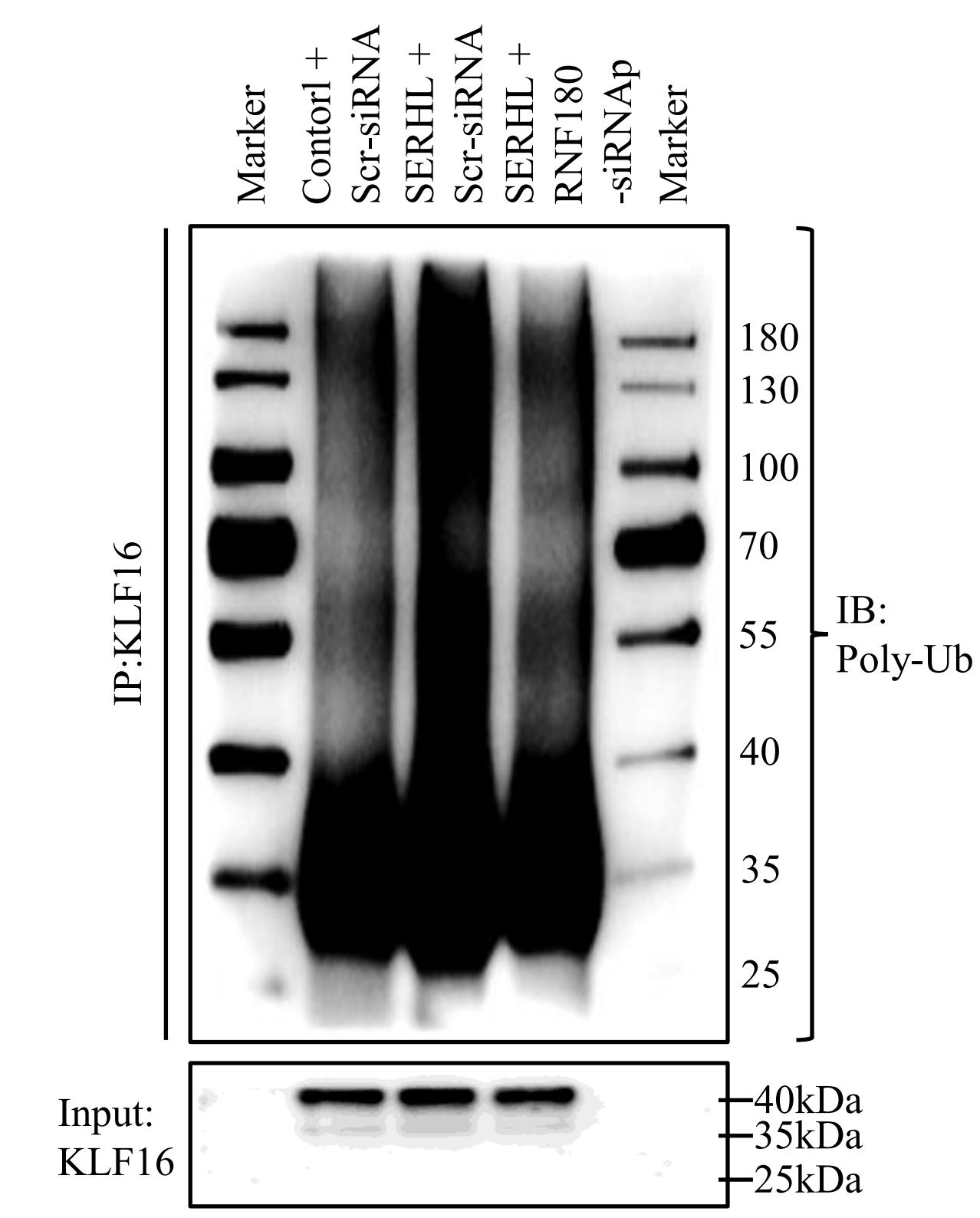

Supplementary Fig.4C2 Gel:10%

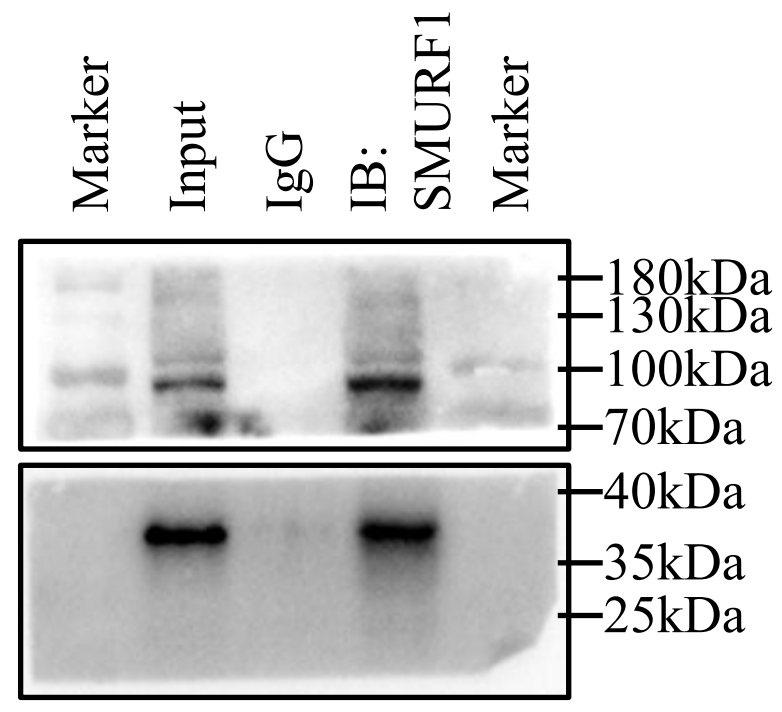

Supplementary Fig.4E Gel:10%

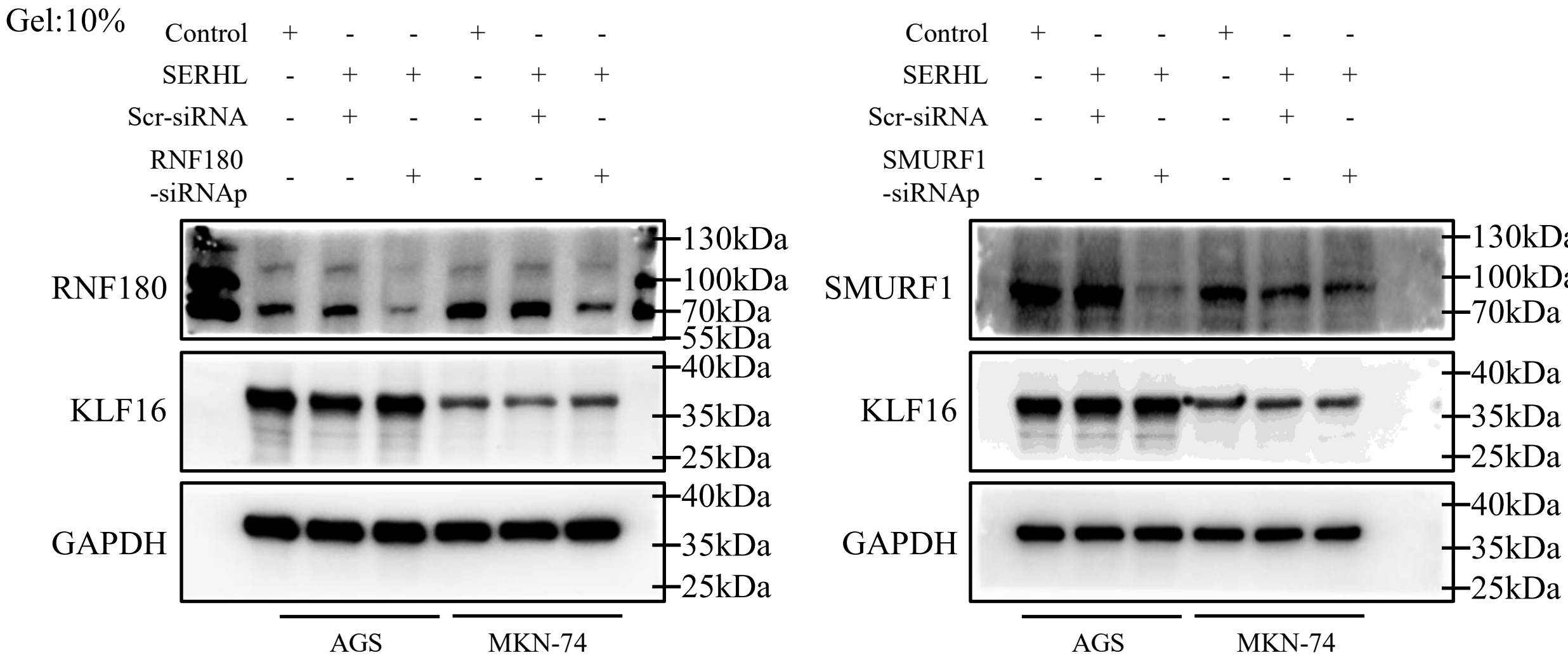

Supplementary Fig.4C3 Gel:10%

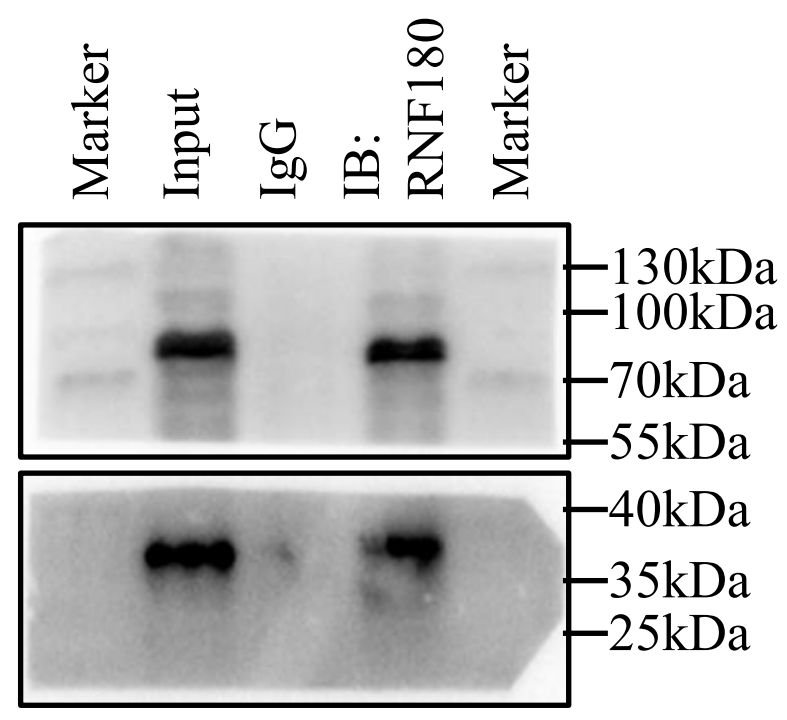

Supplementary Fig.4F Gel:10%

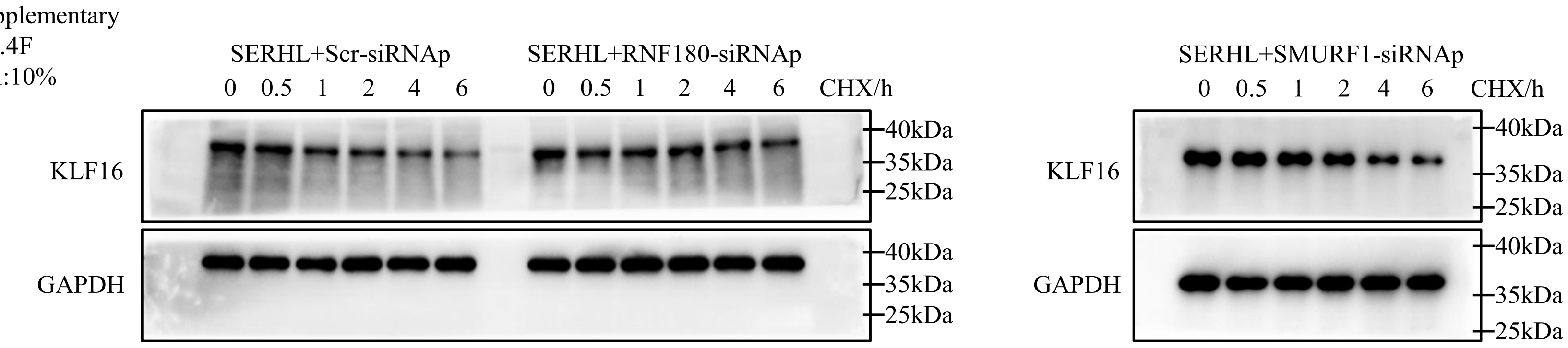

Fig.4A Gel:10%

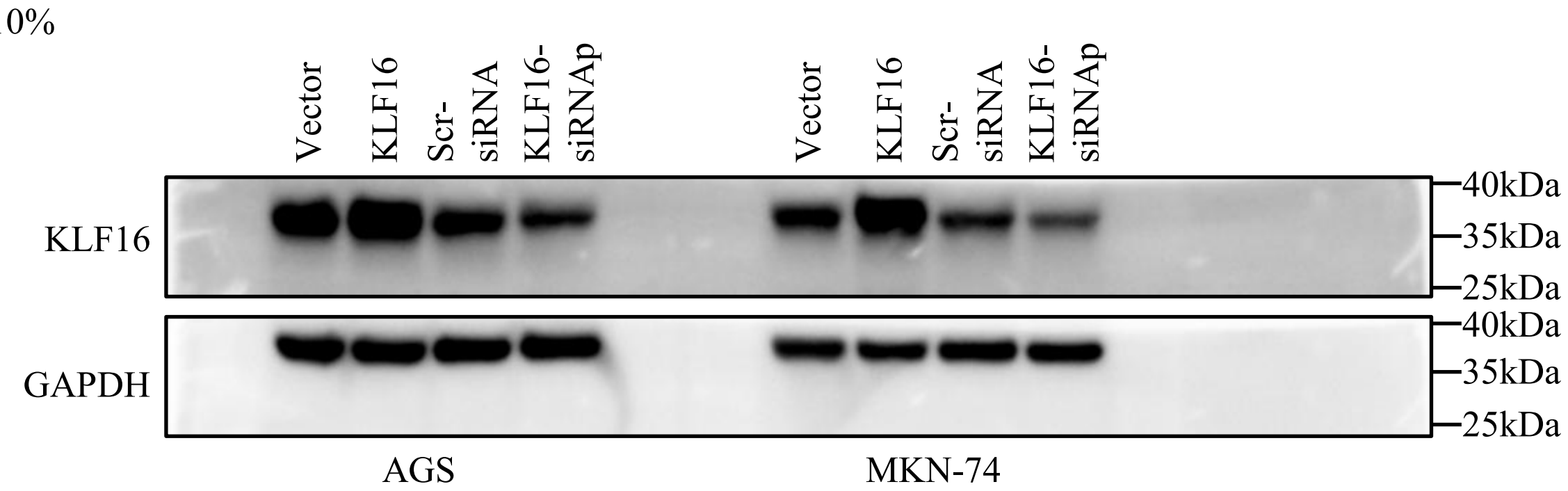

Fig.5D Gel:10%

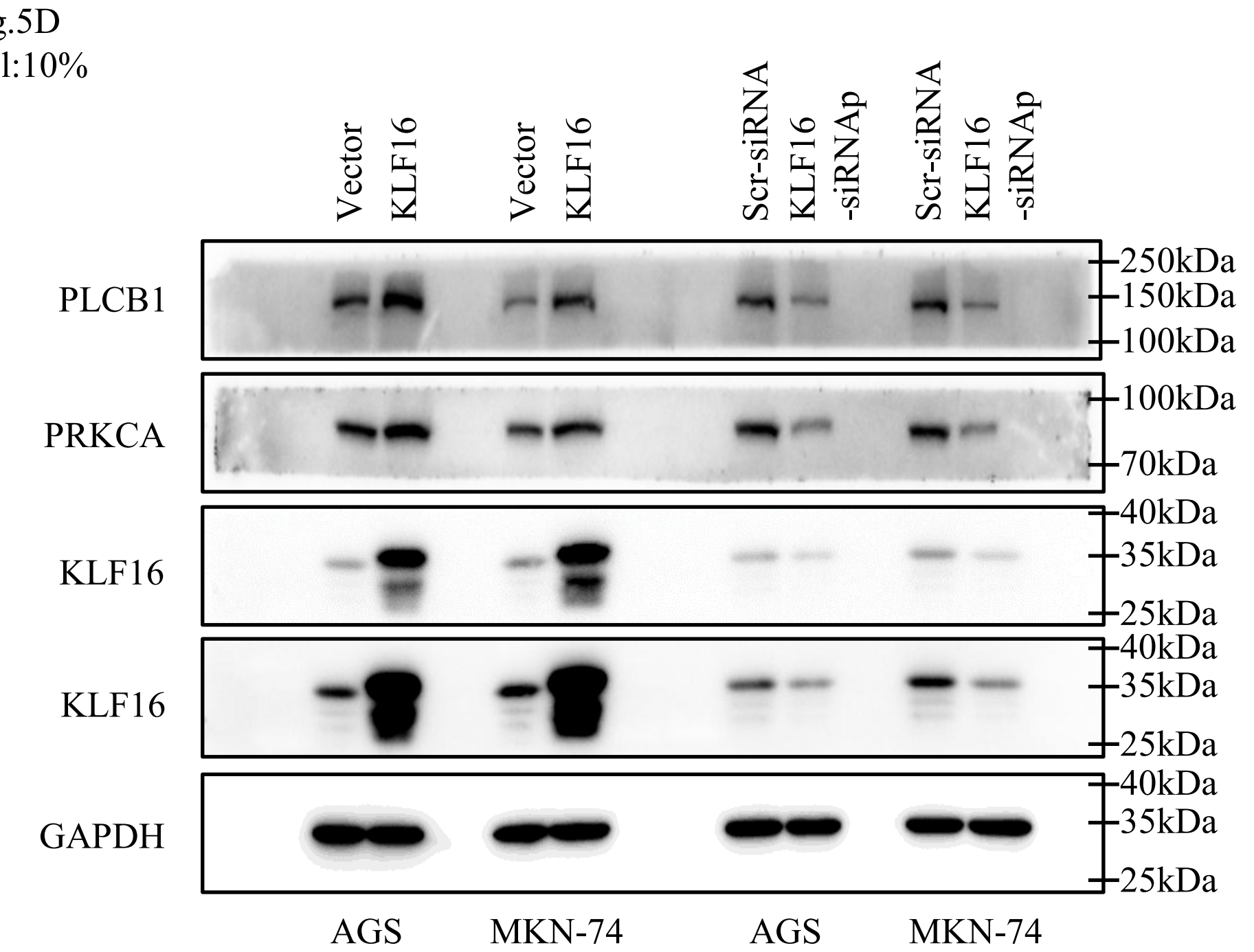

Supplementary Fig.5B Gel:8%

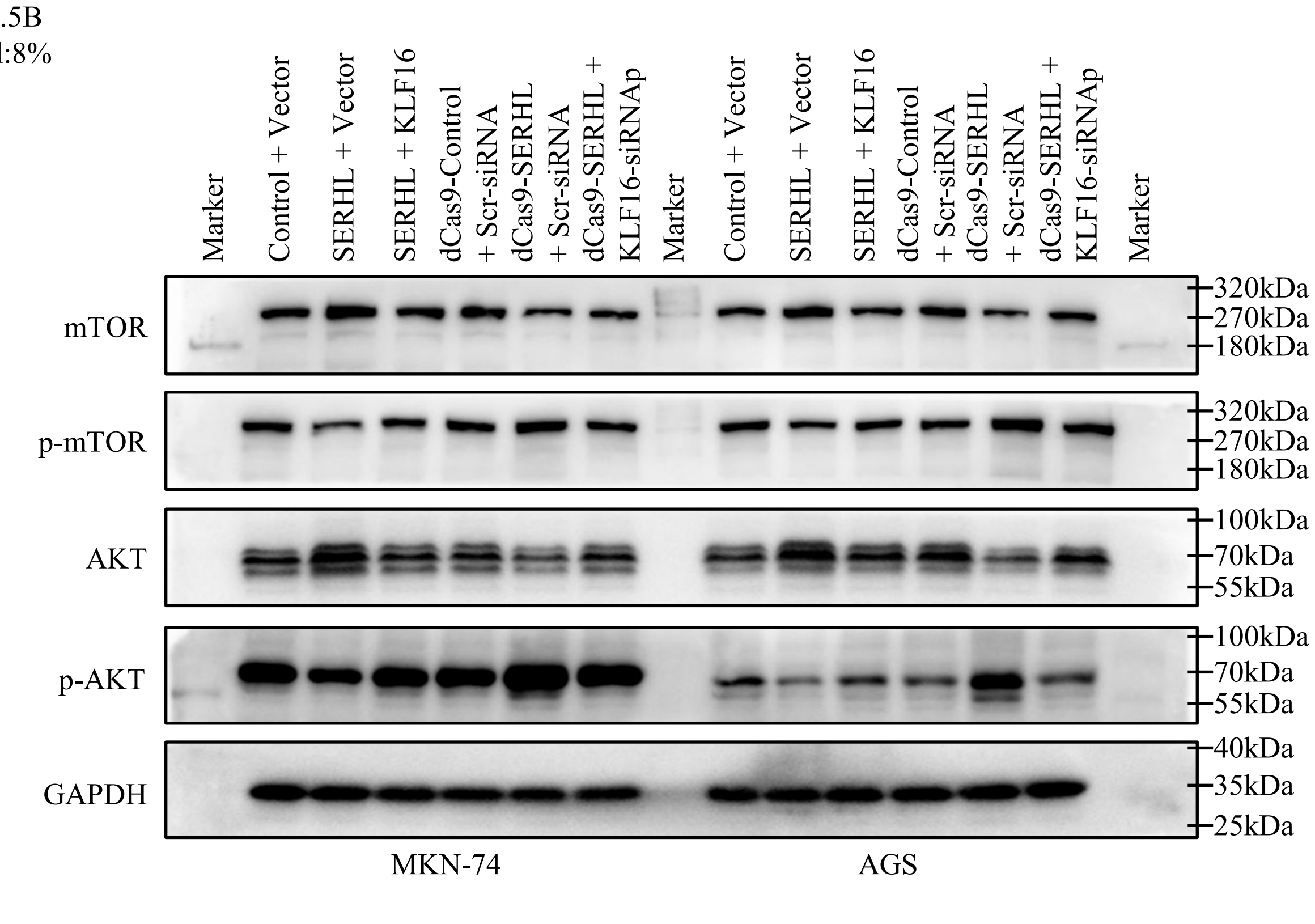

Fig.5G1 Gel:1.5%

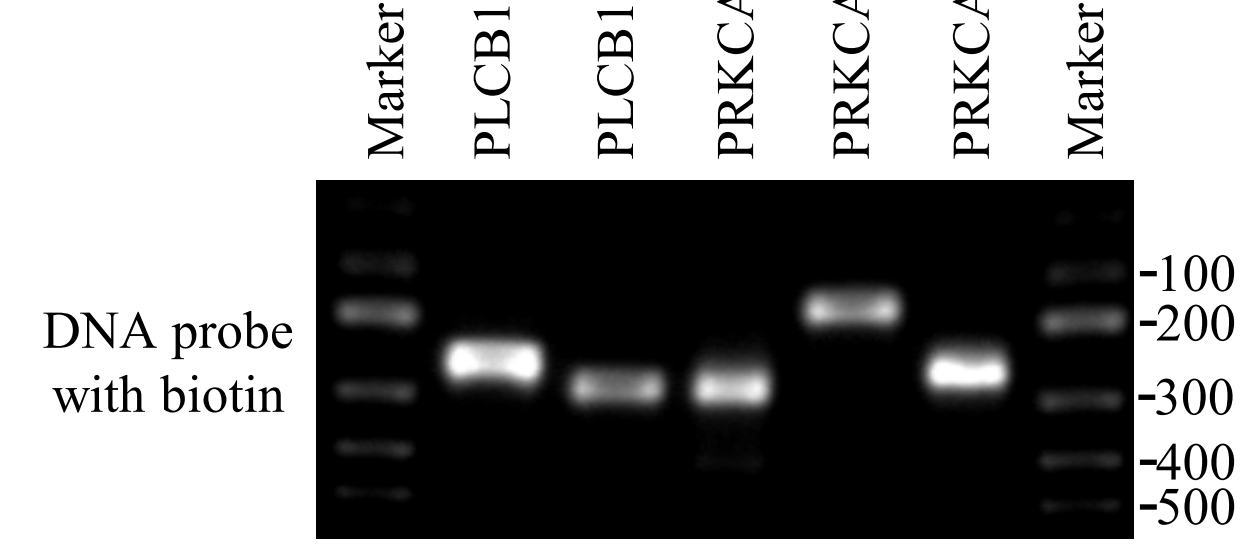

Fig.6A1 Gel:10%

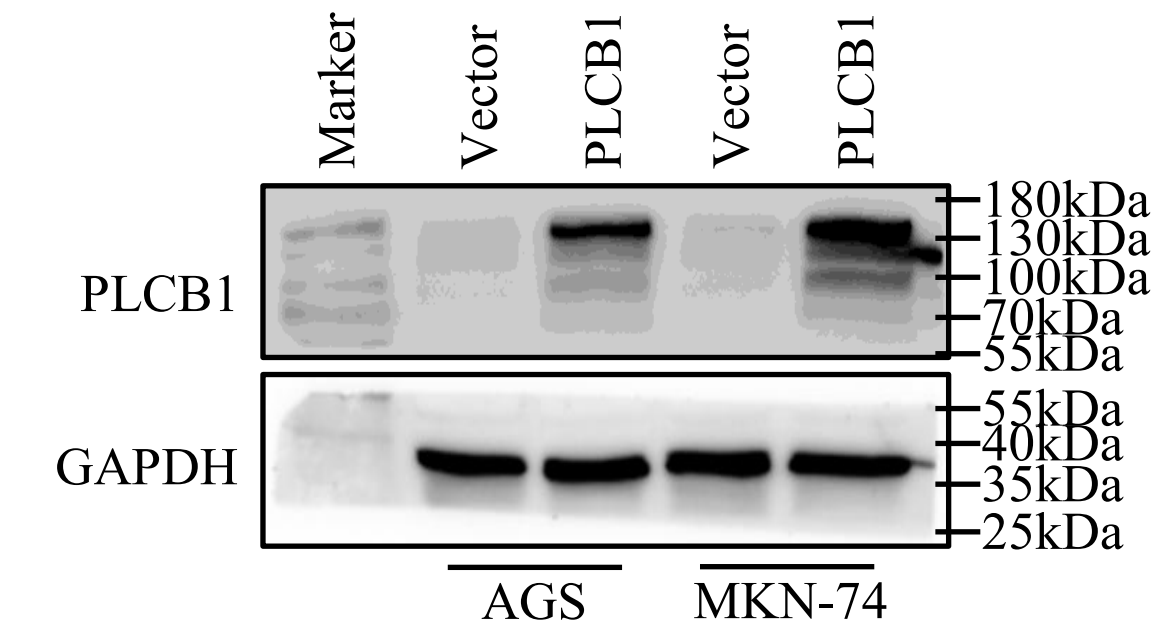

Fig.5G2
Gel:10%

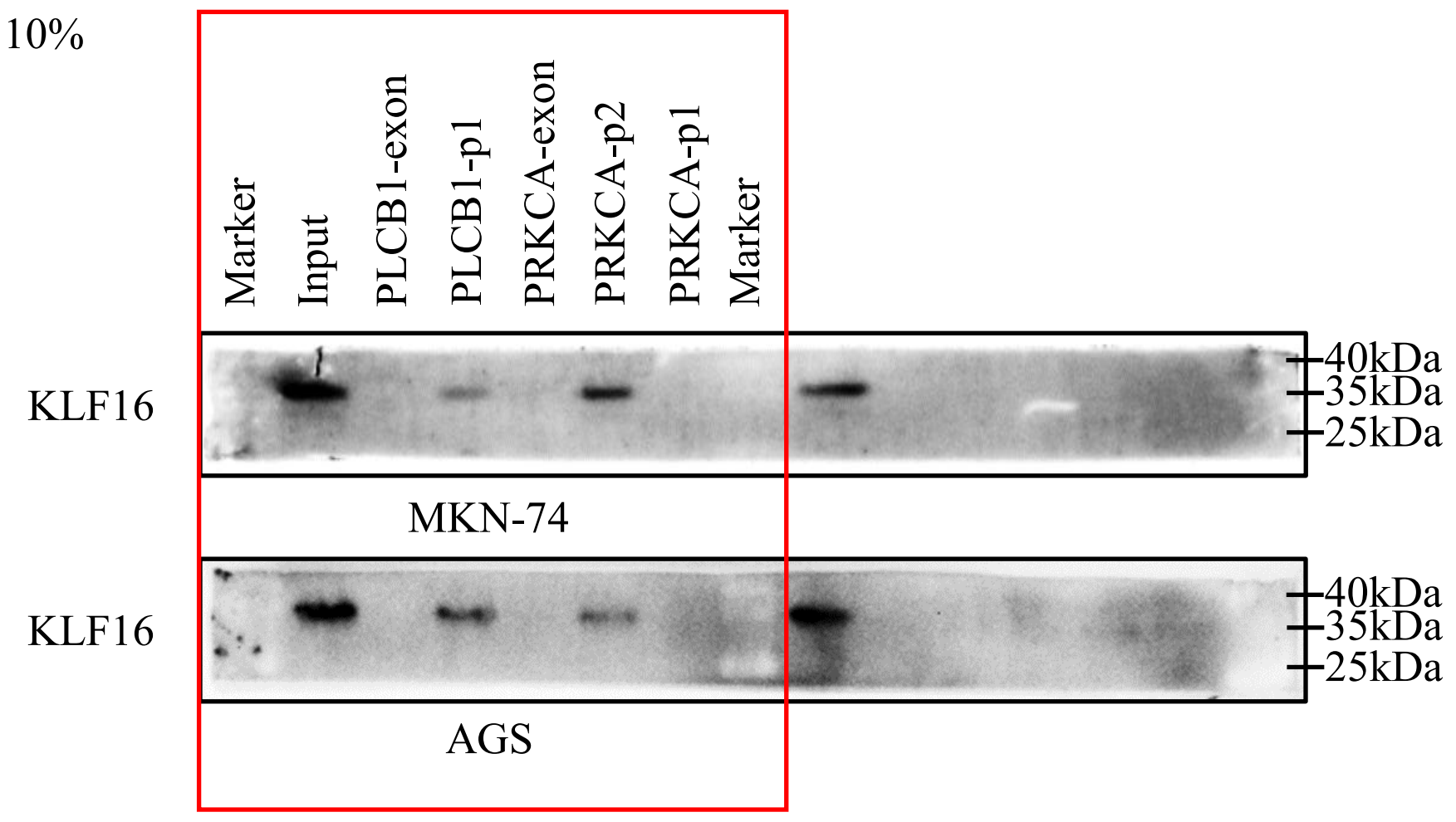

Fig.5I1
Gel:1.5%

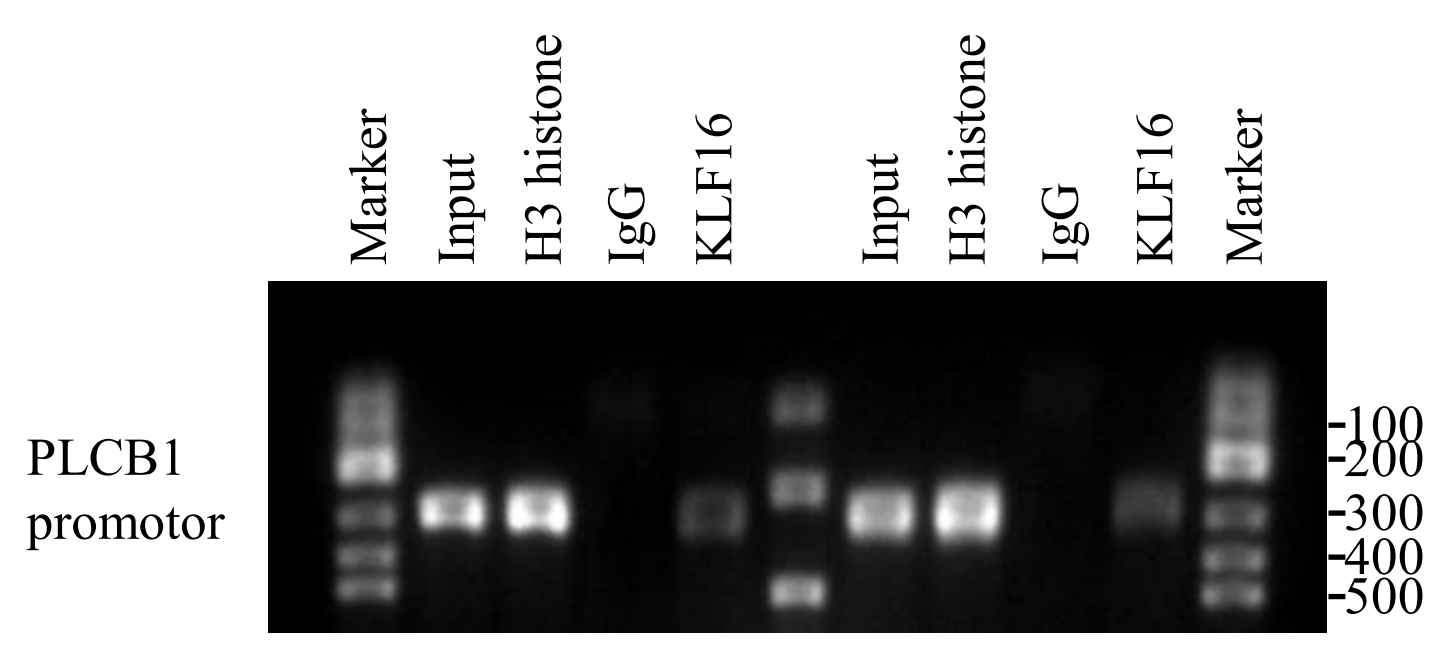

Fig.5I2
Gel:1.5%

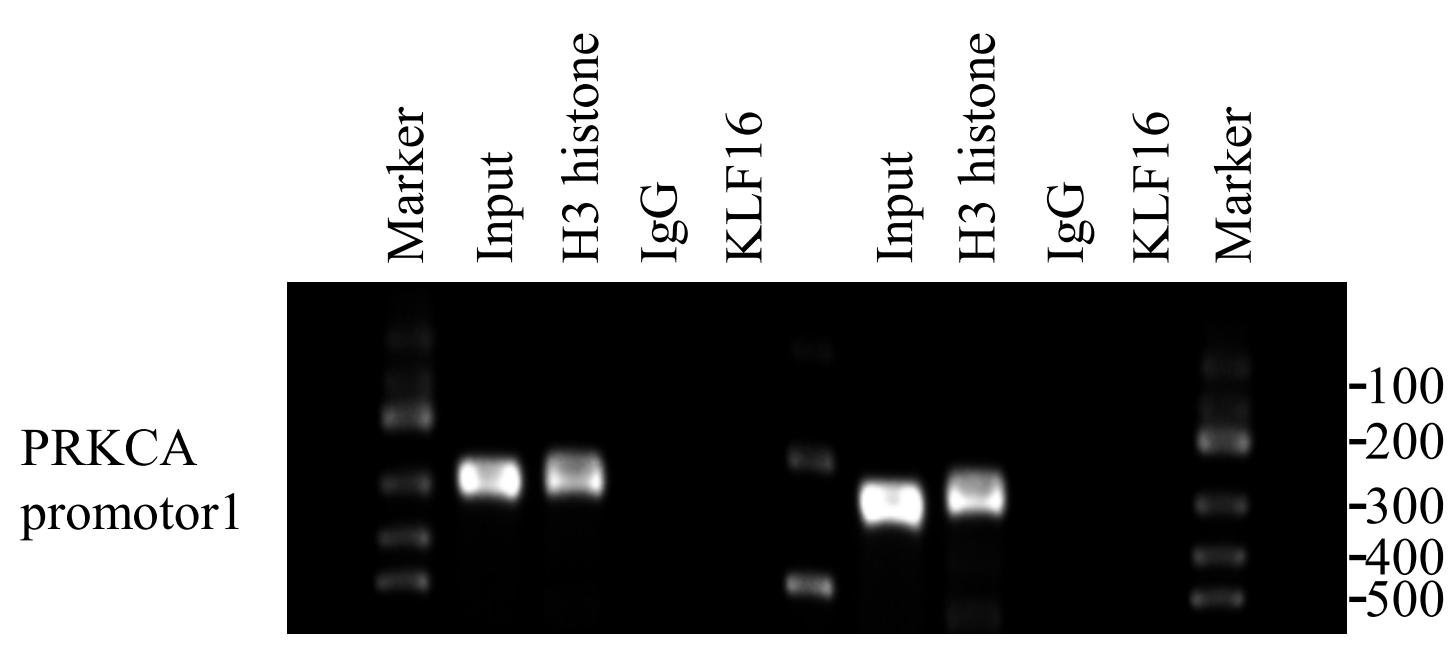

Fig.5I2
Gel:1.5%

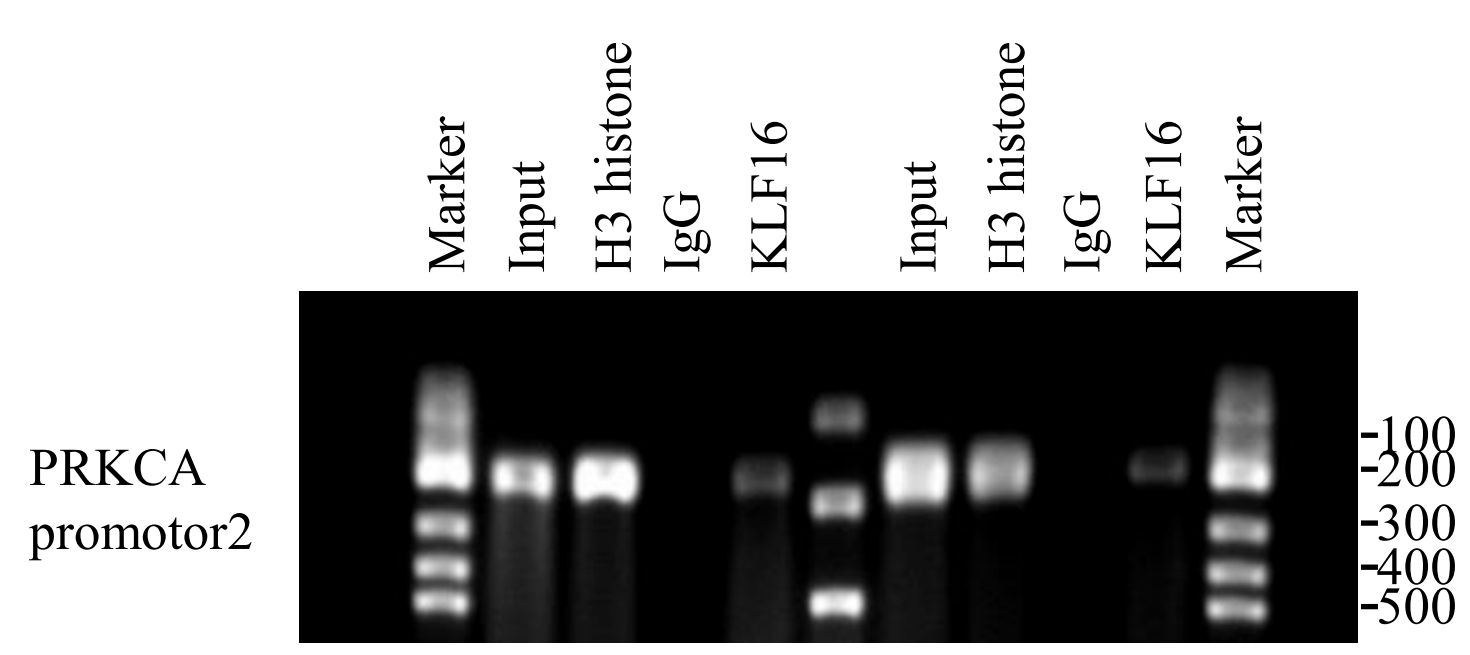

Fig.6A1
Gel:10%

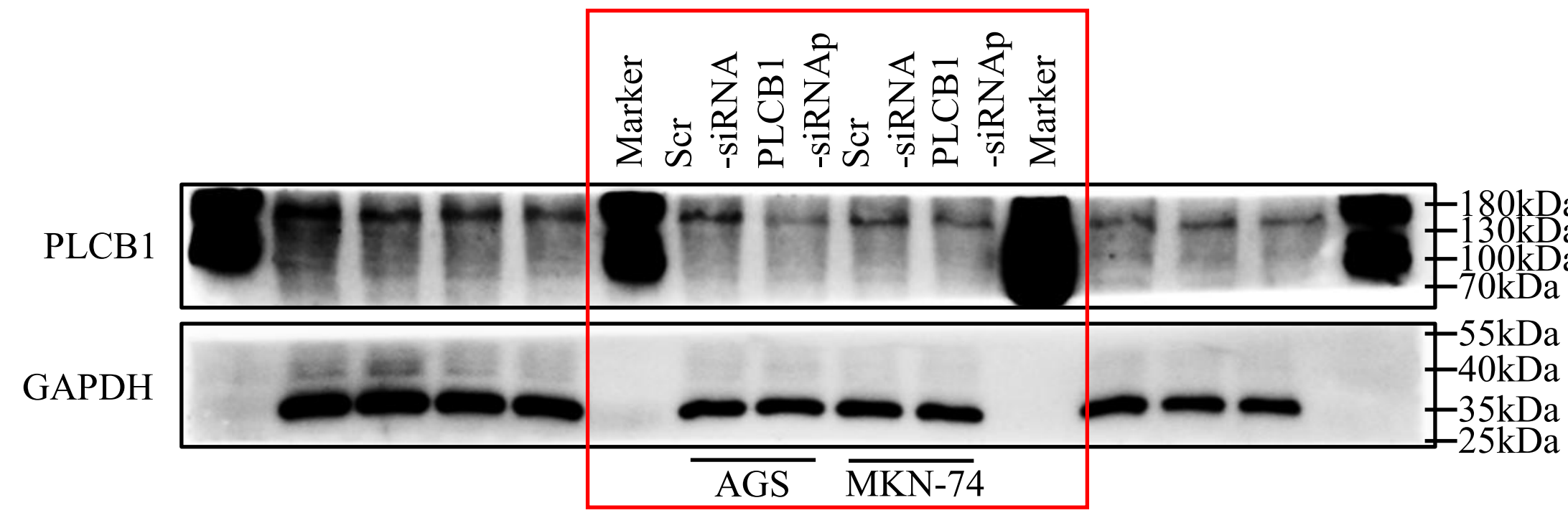

Fig.6A2
Gel:10%

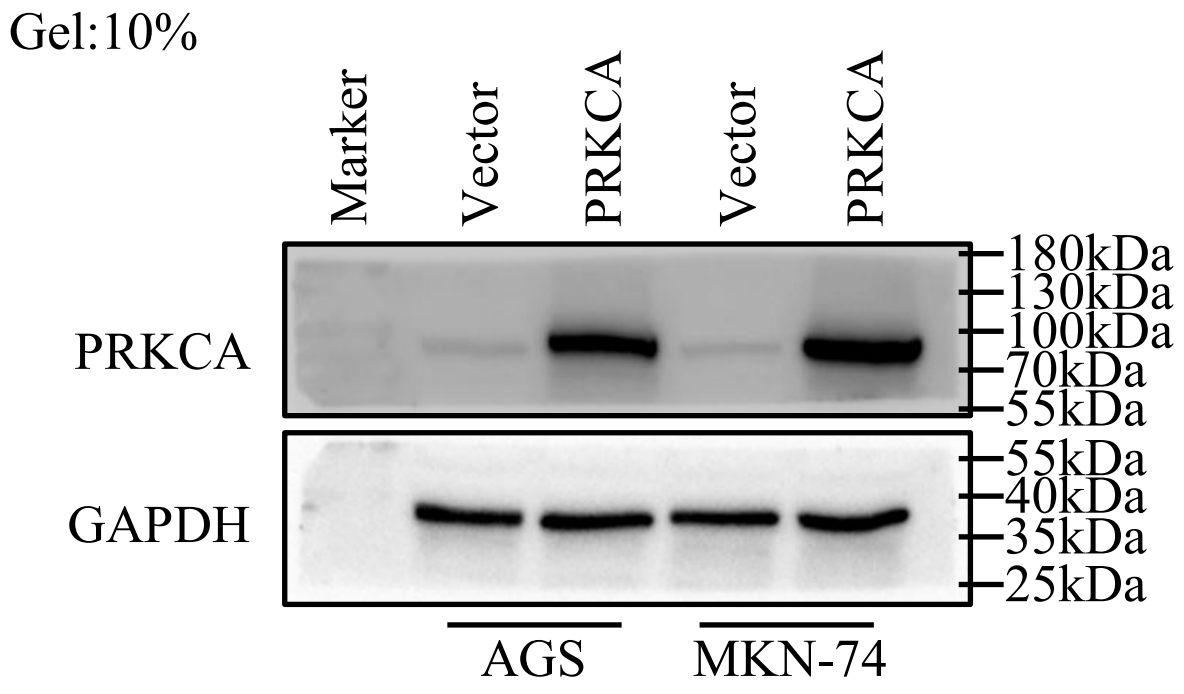

Fig.6A2
Gel:10%

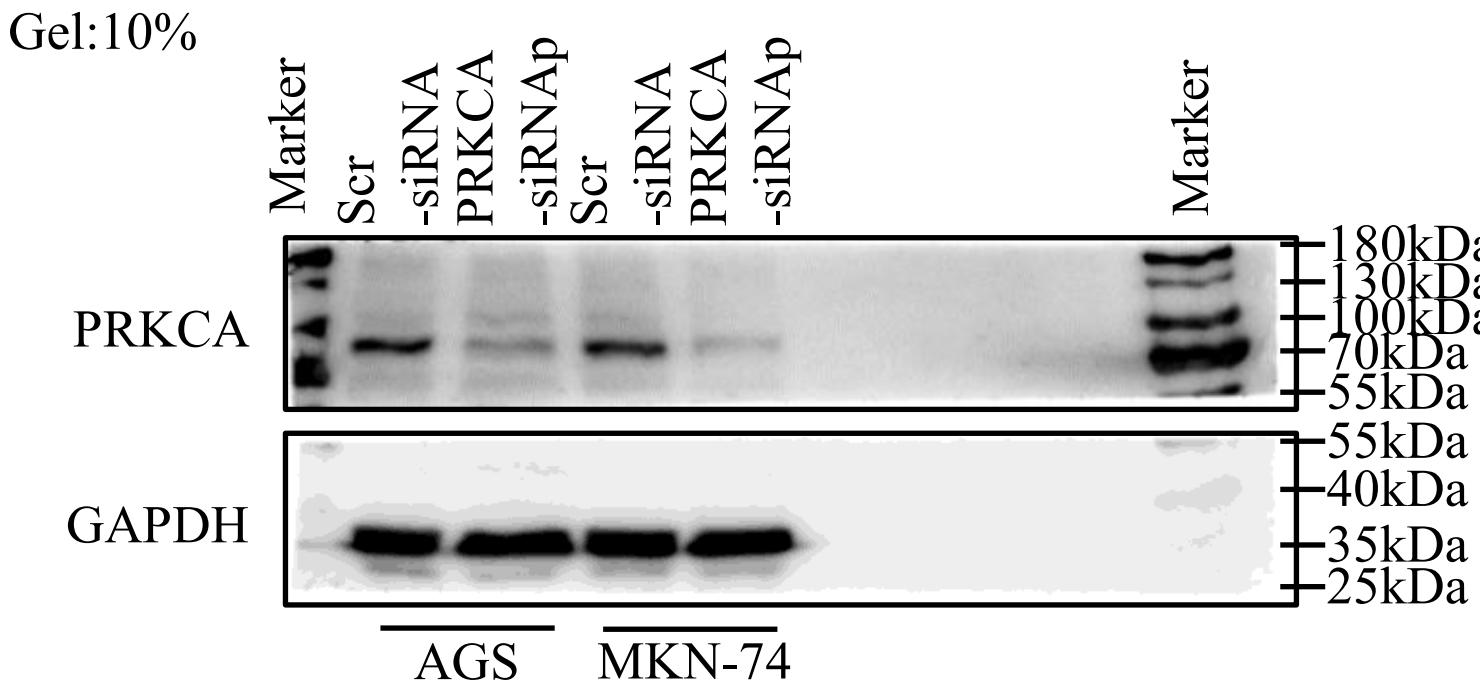

Fig.6D
Gel:8%

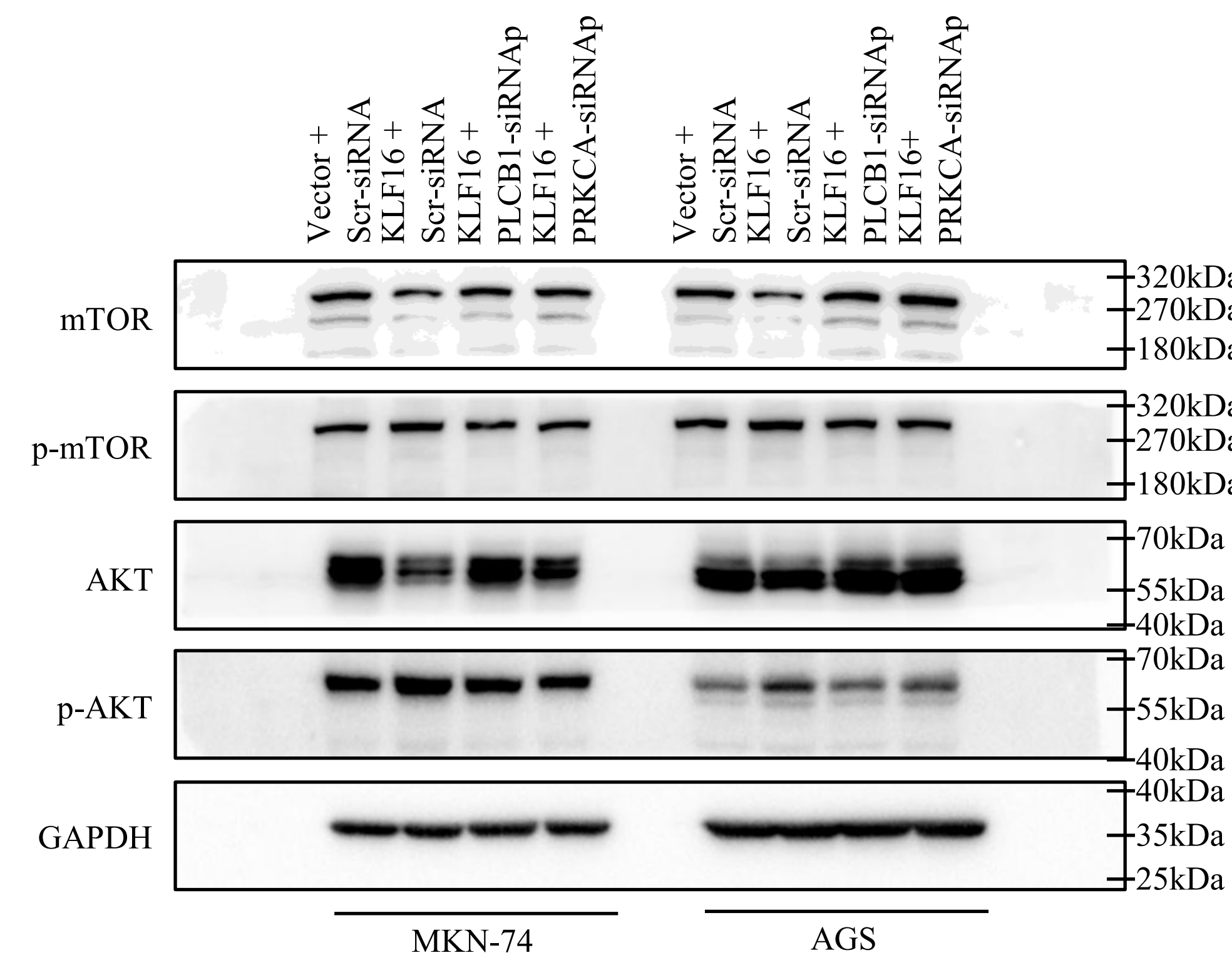

Fig.7A
Gel:10%

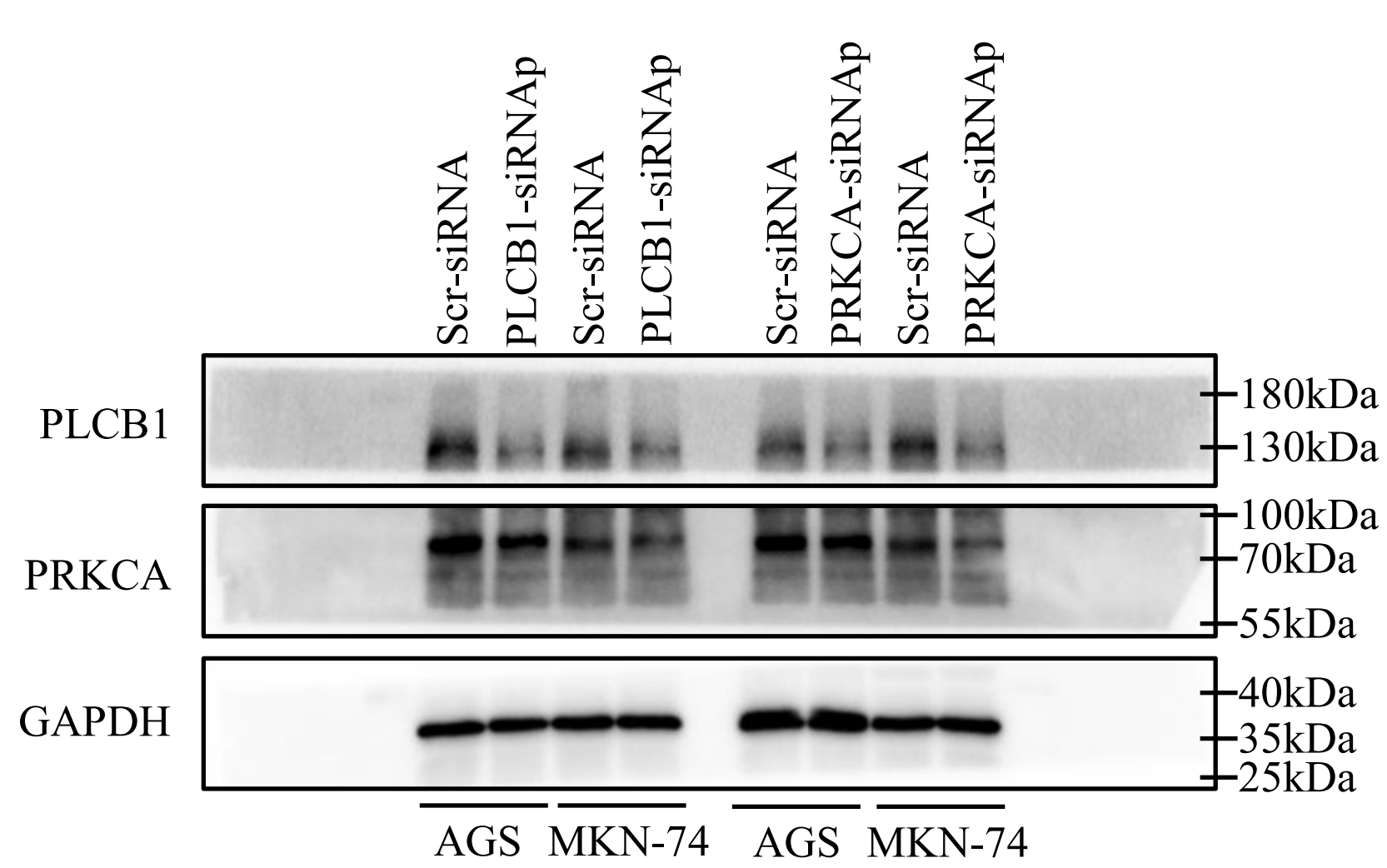

Fig.7B
Gel:10%

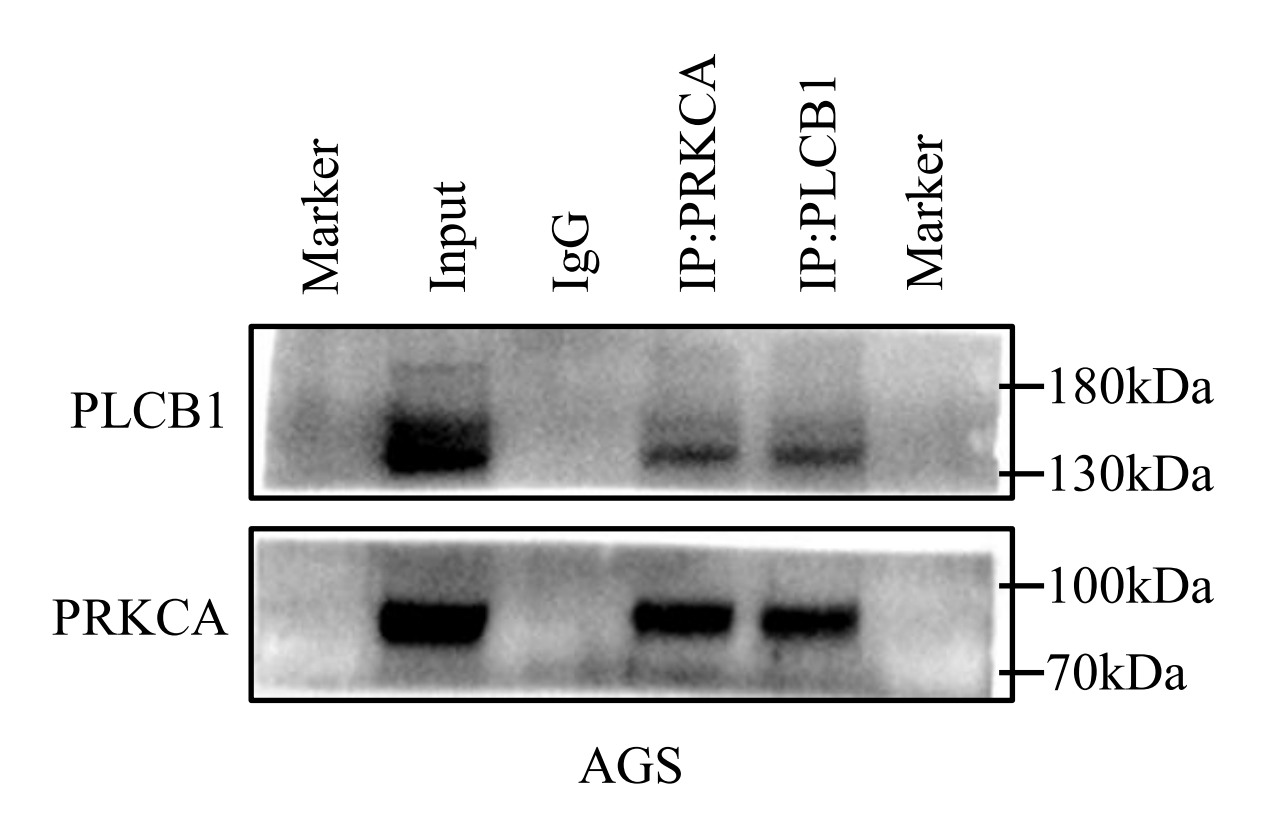

Fig.7B
Gel:10%

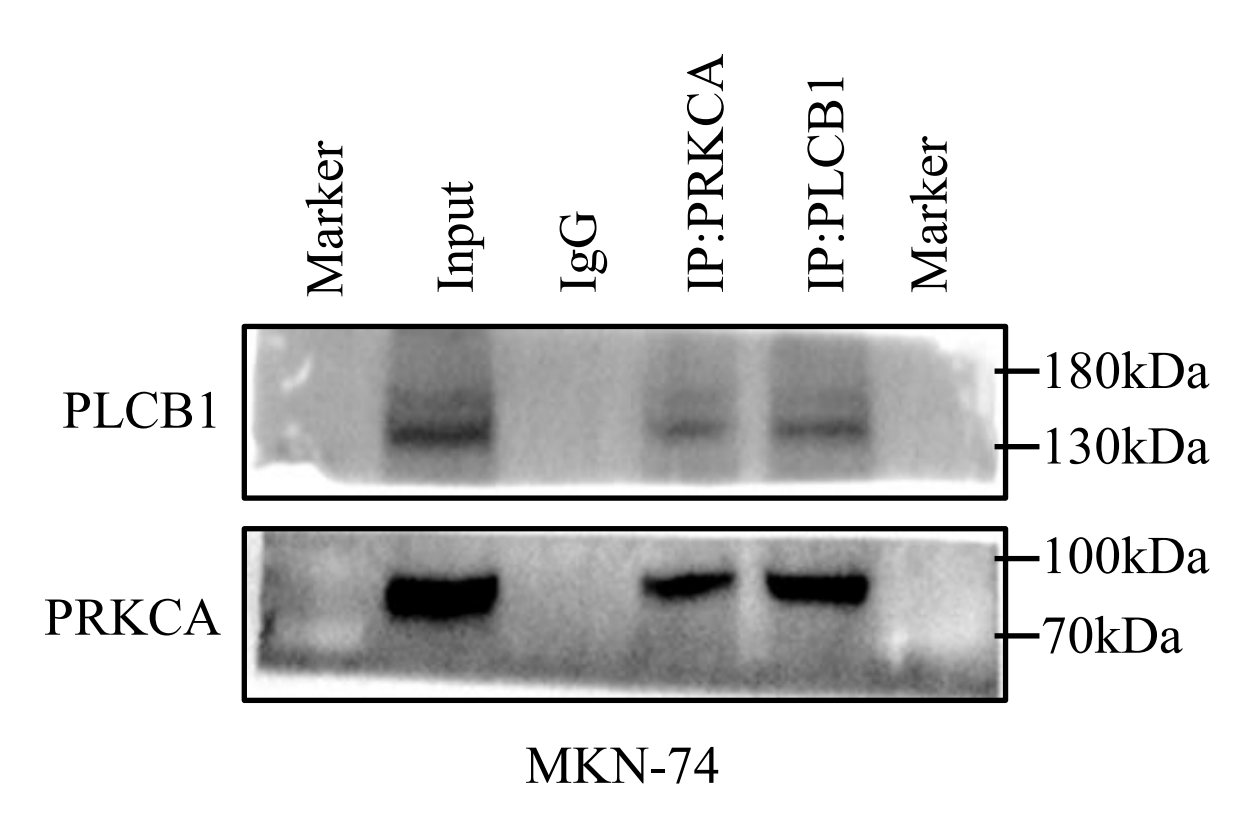

Fig.7C
Gel:10%

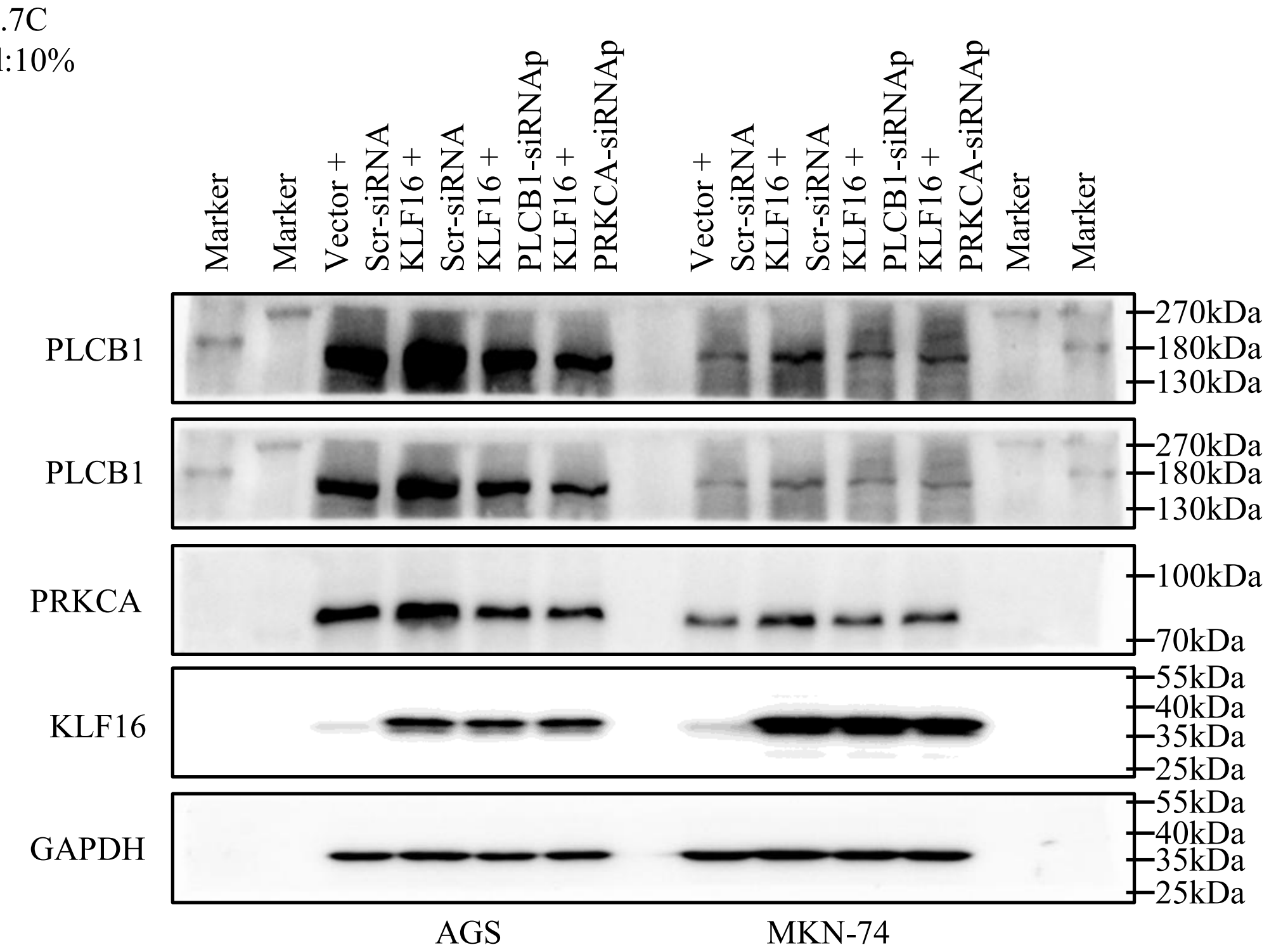

Supplementary
Fig.7F
Gel:8%

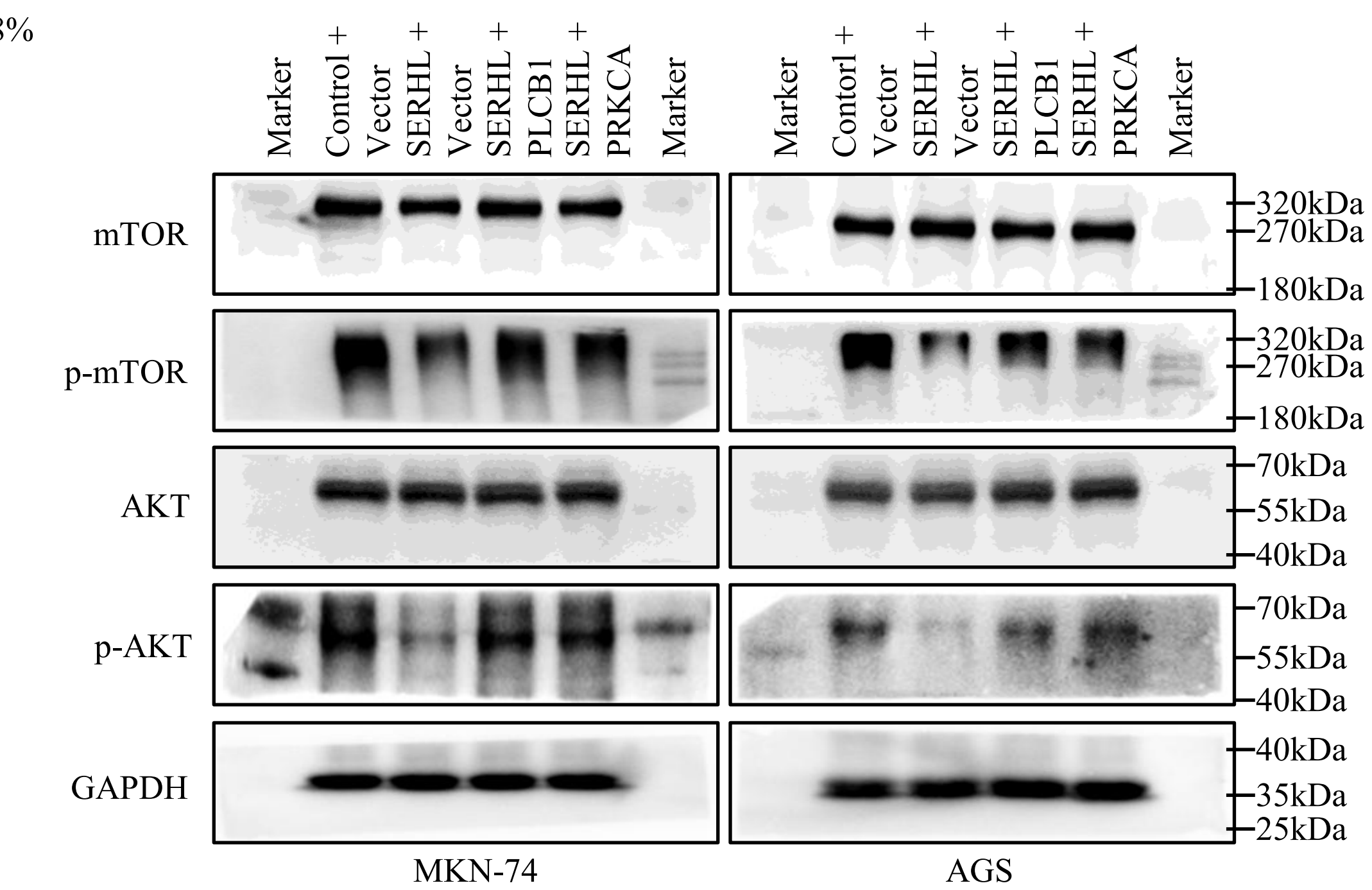

Fig.8A
Supplementary Fig.8A&B
Gel:8%

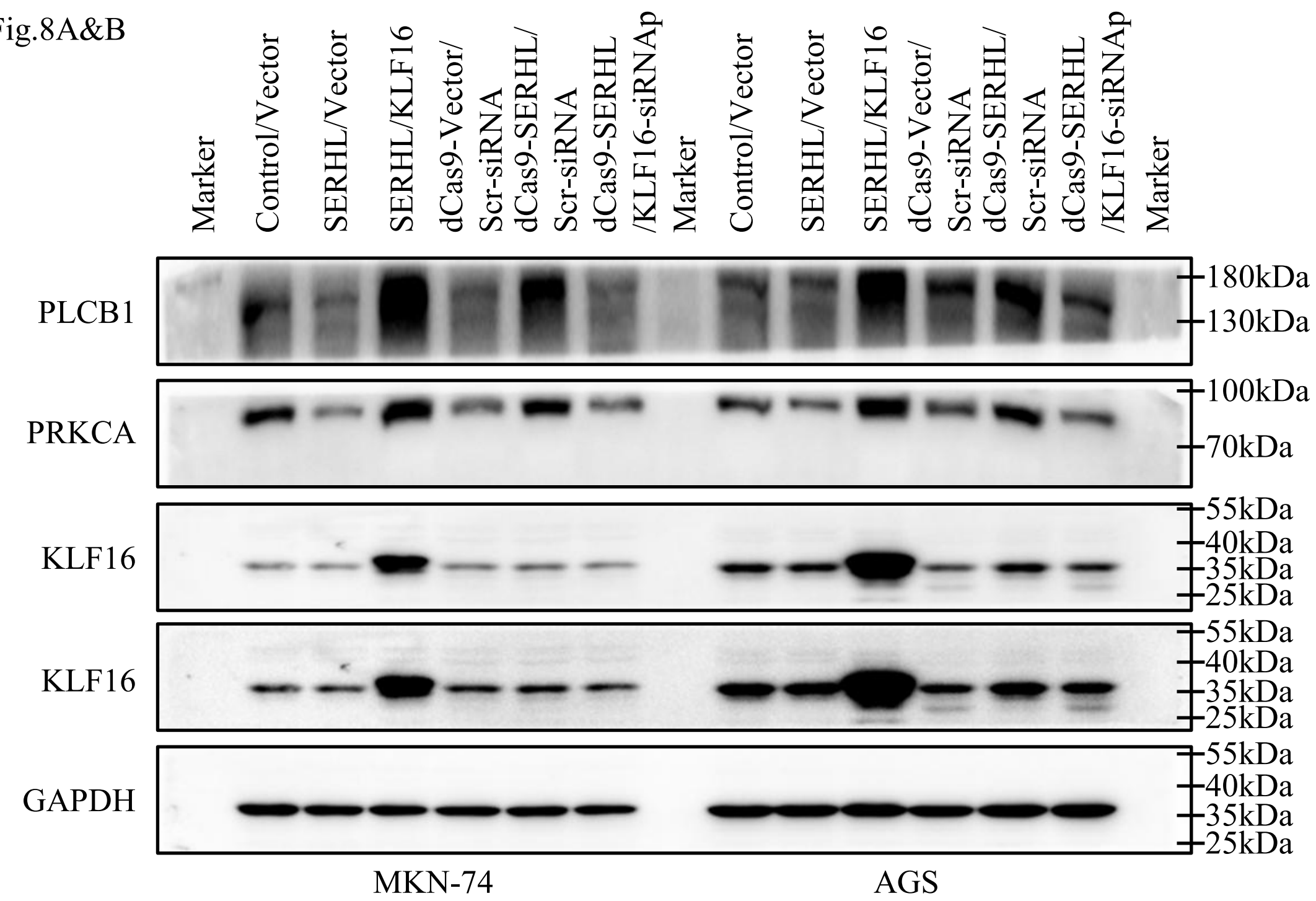

Fig.8B2
Gel:10%

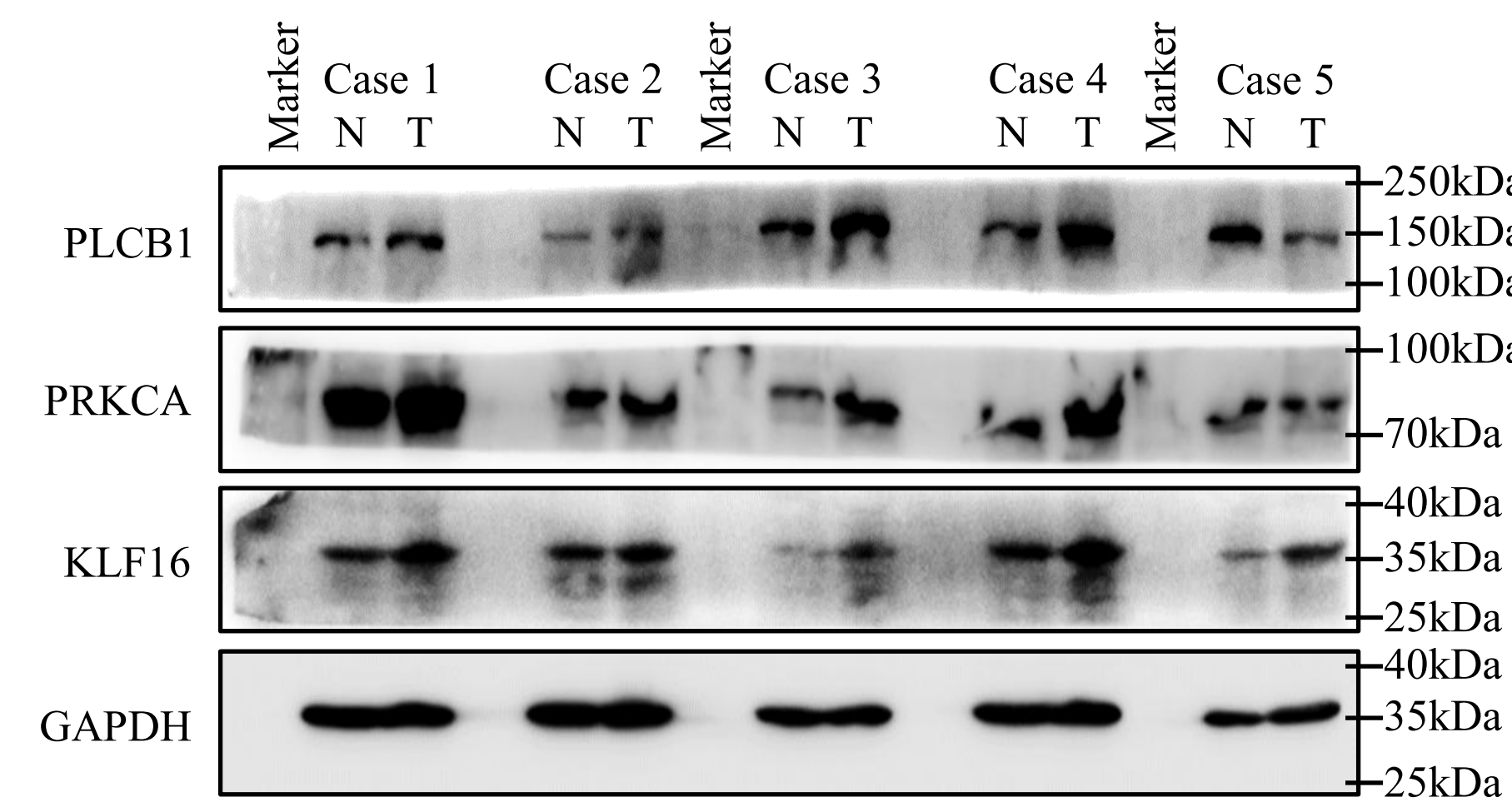

Supplementary
Fig.8C2
Gel:10%

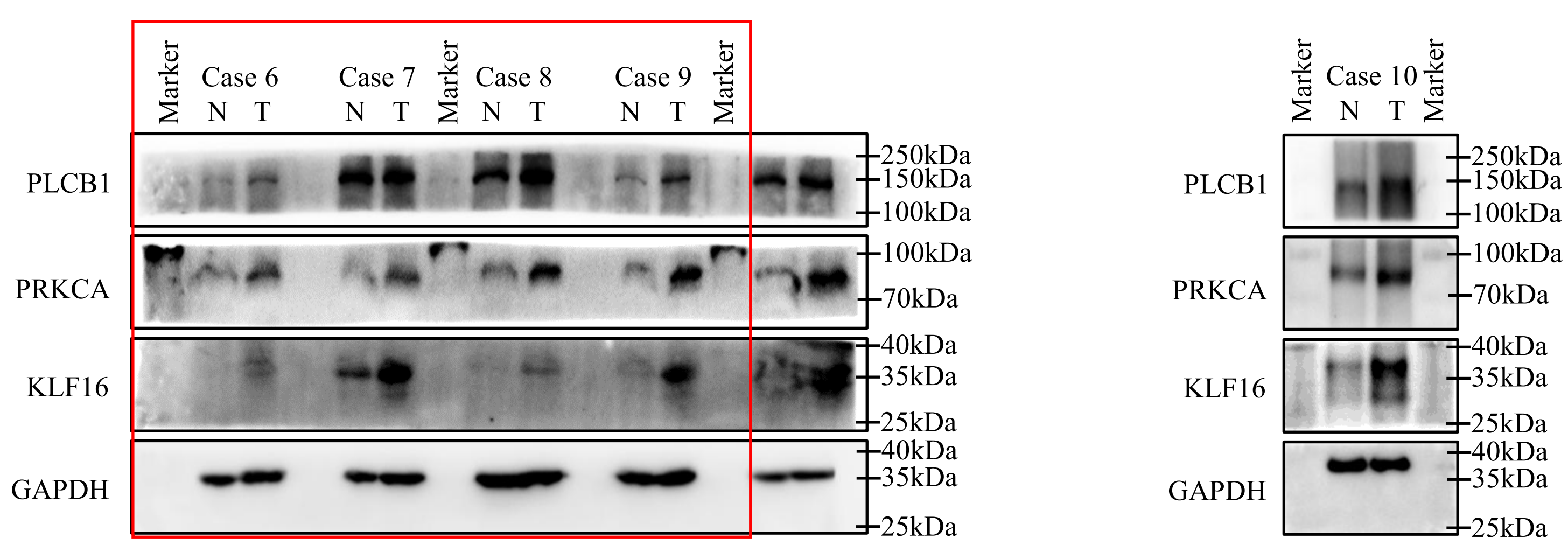

Supplement: Supplementary file 1 [file mmc3.pdf]
