# Supplementary material for: SERHL regulates invasion and metastasis through KLF16-PLCB1/PRKCA signaling in gastric cancer cells
Source: Genes Dis. 2025 Oct 20;13(6):101884. doi: 10.1016/j.gendis.2025.101884 (PMC13486994; doi:10.1016/j.gendis.2025.101884)
Supplement: Supplementary file 2 [file mmc1.docx]

**Supplementary Figure legends**

**Supplementary Fig. 1. Sequence homologies of the human SERHL and mouse Serhl RNA.** **(A)** A schematic diagram of between Human SERHL and mouse Serhl molecules. X, Breakpoint in protein-coding process. **(B)** Sequence alignment of Human SERHL and mouse Serhl nucleotides. Human SERHL and mouse Serhl RNA sequence results showed 81.32% (Per. Ident) using the human genome databank, NCBI BLAST. Dots indicate identities.

**Supplementary Fig. 2. SERHL is downregulated in GC tissues. (A)&(B)** The SERHL RNA expression pattern in normal and tumor tissues from various cancers generated by the GEPIA (http://gepia.cancer-pku.cn/, A) and Firebrowse (http://firebrowse.org/, B) databases. The expression in GC and normal tissue is highlighted by blue box.

**Supplementary Fig. 3. Ectopic expression of SERHL inhibited GC cell invasion and metastasis, proliferation. (A)** Invasion assays were conducted on GC cells transfected with either Control and SERHL (A1) or dCas9-control and dCas9-SERHL (A2). ***, P < 0.01 and ****, P < 0.001. **(B)** DNA synthesis was assessed 48 hours post-transfection using an EdU incorporation assay, where green fluorescence indicates EdU-positive cells and blue fluorescence from Hoechst stain indicates total cells. ****, p<0.001. Control vs. SERHL; ****, p<0.001, dCas9-control vs. dCas9-SERHL. **(C)** Colony formation assay to evaluate SERHL's influence on GC cell proliferation. ****, p<0.001. Control vs. SERHL; ****, p<0.001, dCas9-control vs. dCas9-SERHL. (**D**) Injection of AGS/control and AGS/SERHL cells into nude mice resulted in subcutaneous tumors. (**E1/2**) Tumor nodule weight (E1) and volume (E2) were recorded. **(F1/2)** Subcutaneous tumors in nude mice were stained with H&E and Ki-67 IHC (F1). The rate of Ki-67 positive expression was assessed (F2). Scale bars, 100 μm in (F1).

**Supplementary Fig. 4. SERHL interacts with KLF16 protein and mediates its ubiquitination and degradation via E3 ligase RNF180. (A)** Random Forest (RF) and Support Vector Ma­chine (SVM) predicted the RNA-protein interac­tions of SERHL-STAT2 (A1), SERHL-HNF4A (A2) and SERHL-KLF16 (A3) by RPISeq database. The probability threshold used for "positive" interactions was 0.50. **(B)** Potential ubiquitin E3 ligases for KLF16 were analyzed by the UbiBrowser platform. **(C)** Co-IP assays evaluated the endogenous interaction between KLF16 and SMURF1 or RNF180 in GC cells. **(D)** SMURF1 or RNF180 protein expression levels were analyzed in GC cells 48 h post-transfection. **(E)** Western blot analysis of SMURF1/RNF180 and KLF16 expression in GC cells co-transfected with SERHL and SMURF1/RNF180-siRNAp (siRNAp, siRNApool). **(F)** AGS cells with different groups were treated with CHX (100 μM) for the indicated periods to analyze KLF16 protein levels by western blot. **(G)** Ubiquitin levels of KLF16 were measured in Vector+ Scr-siRNA, SERHL+Scr-siRNA, or SERHL+ RNF180-siRNAp AGS cells using IP and western blot. Poly-Ub, Poly-ubiquitin.

**Supplementary Fig. 5. KLF16 mediate the effects of SERHL on tumor invasion** **via AKT/mTOR pathway** **in GC cells. (A)** The wound healing assay was employed to assess the migration capacity among AGS cells that received different treatments. ******, p < 0.05, ***, P < 0.01, and ****, P < 0.001. **(B)** The analysis of AKT/mTOR signaling pathway among different treatment groups of GC cells by western blot.

**Supplementary Fig. 6.** KEGG analysis base on downregulated genes in KLF16-shRNA transfected AGS cells according to RNA sequencing.

**Supplementary Fig. 7. SERHL modulate invasion and metastasis through KLF16-PLCB1/PRKCA signaling in GC cells. (A)&(B)** Transwell assays were used to evaluate migration and invasion changes in different GC cells. ***, p<0.01; ****, p<0.001. **(C)&(D)** To measure GC cell motility, the wound healing assay was utilized. ****, P < 0.001. **(E)** Comparison of MMP2 IRS between difference groups. **, p<0.05. **(F)** The analysis of AKT/mTOR signaling pathway among co-transfected SERHL and PLCB1/PRKCA groups of GC cells by western blot.

**Supplementary Fig. 8. Inverse correlations of SERHL with KLF16, PLCB1, and PRKCA expression in GC tissues. (A)&(B1/2)** KLF16, PLCB1, and PRKCA expression were analyzed via western blot in GC cells co-transfected with Control/Vector, SERHL/Vector, and SERHL/KLF16 (A) or dCas9-control/Scr-siRNA, dCas9-SERHL/Scr-siRNA and dCas9-SERHL+KLF16-siRNAp (B1/2). **(C)** In another five GC tissue samples, the expression levels of SERHL (C1), KLF16, PLCB1, and PRKCA (C2) were assessed by qPCR (C1) or western blot (C2), normalized to B2M or GAPDH. *, p > 0.05; **, p < 0.05, ****, p < 0.001, T vs. N. T, cancer; N, normal. **(D)** ISH and IHC were performed to determine SERHL, KLF16, PLCB1 and PRKCA expression patterns in ten GC tissues and corresponding normal tissues. Scale bars, 100 μm in (D). **(E)** Survival analysis in the GSE15459 cohort was performed to examine the overall survival associated with each of the four molecules separately.
